# Two-ring chirality generated by the alignment of two achiral phenylacetylene macrocycles†

**DOI:** 10.1039/d3ra01780j

**Published:** 2023-04-14

**Authors:** Ryo Katoono, Kohei Arisawa

**Affiliations:** a Department of Chemistry, Faculty of Science, Hokkaido University Sapporo 060-0810 Japan katoono@sci.hokudai.ac.jp +81-11-706-4616

## Abstract

When two achiral rings are bound mechanically, a chiral source is generated in the assembly. The chiroptical properties could be modulated according to the relative occupation of each ring in the assembly. In fact, we have found that two isomeric assemblies (1 and 2) show unique properties in each assembly with two achiral rings of phenylacetylene macrocycle (PAM). When considering the difference in the chiroptical properties of these two isomeric assemblies (6PAM × 2), no comparison was available based on no activity of the achiral component element itself (6PAM). In this work, we synthesized a two-ring chiral analog (4) by the ring-fusion of two 6PAMs to an 11PAM, and examined the chiroptical properties of 4, since the single helix was imparted as a chiral source. By comparison of the chiroptical properties (molar circular dichroism and molar optical rotation) of 1 and 2 to those of 4, we demonstrated that the disparity was related to the alignment of the two achiral rings.

## Abstract 2

Chiroptical properties vary with the alignment of components based solely on the identical achiral ring.

## Graphical abstract

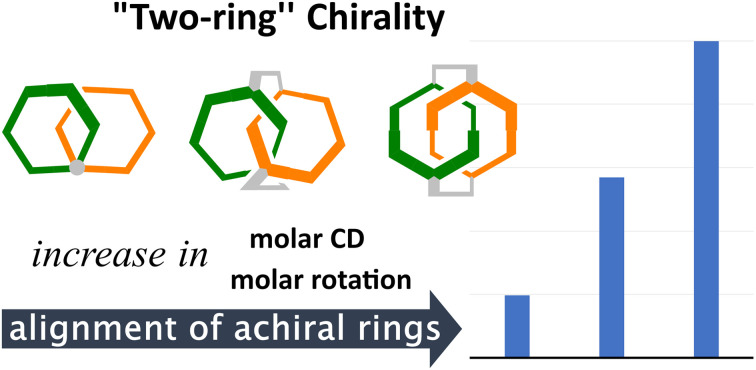

## Introduction

**Scheme 1:**
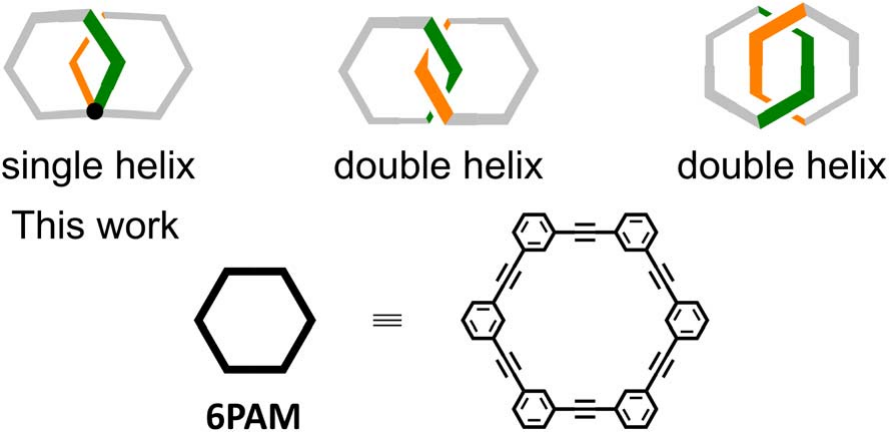
Two-ring chirality generated by the alignment of two rings of 6PAM.

For a chiral molecule^1,2^ that is composed of two or more optically-active molecular units (*e.g.*, amino acids,^3^ binaphthyls,^4^ paracyclophanes,^5^ helicenes,^6,7^ and so on), it is not settled how the overall chiroptical activity is related to the original activity of the molecular unit itself. The properties have often been described in terms of a multiple of those of smaller optically-active units. However, such a comparison would not be valid for a class of chiral molecules that is composed of only achiral elements.^8–15^ In this regard, we have been interested in chiral molecules based on the assembly of achiral rings of phenylacetylene macrocycle (PAM).^16–21^ Two achiral rings were helically-stacked one above the other (6PAM × 2), or bound to each other mechanically (6PAM × 2). As an alternative, a single enlarged ring of 12PAM was also assumed to be a doubled form of 6PAM. Unique Cotton effects were observed for each of these diverse assemblies based solely on the identical achiral component.^22^ Especially, we found a remarkable disparity in chiroptical activity for two isomeric species with the two rings bound mechanically (6PAM × 2). To discuss this disparity, we were interested in a double-helix structure generated in the assembly of the two rings (Scheme 1). Since no comparison was available for these molecules based on no activity of the achiral ring itself (6PAM), here we designed a two-ring analog,^8^ where the double helix in 6PAM × 2 is replaced by the single helix as a chiral source (Scheme 1).

**Fig. 1:**
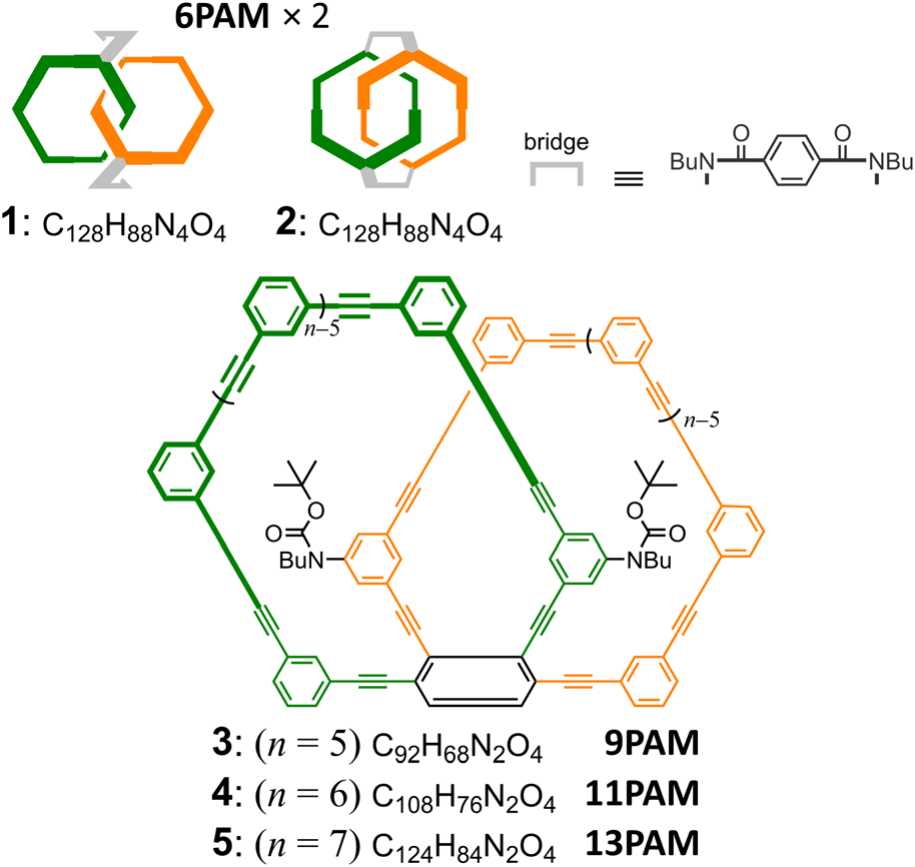
Chemical structures of 1–2 (6PAM × 2),^22^3 (9PAM), 4 (11PAM) and 5 (13PAM). Only one enantiomer is depicted for two-ring chiral 1–5.

The two rings bound mechanically in the assemblies 1 and 2 were doubly bridged to exert chirality, and the absolute configuration was assured^23^ (Fig. 1). The two-ring chiral 3–5 did not need a bridge, since the relative occupation of the two rings is invariable by sharing one benzene ring in each PAM. Thus, the ring fusion assured the configuration of the single helix.

We describe below the synthesis of two-ring chiral 4 by ring fusion of 6PAMs to 11PAM, and consider the disparity in the chiroptical properties between two double-helix structures generated in 1 and 2 (6PAM × 2) through comparisons of the molar circular dichroism (CD) and molar optical rotation to those of the single-helix structure in 4 (11PAM). Two-ring chiral analogs, 3 (9PAM) and 5 (13PAM), were systematically synthesized as an aid to characterization.

## Results and discussion

### Synthesis and assignment

**Fig. 2:**
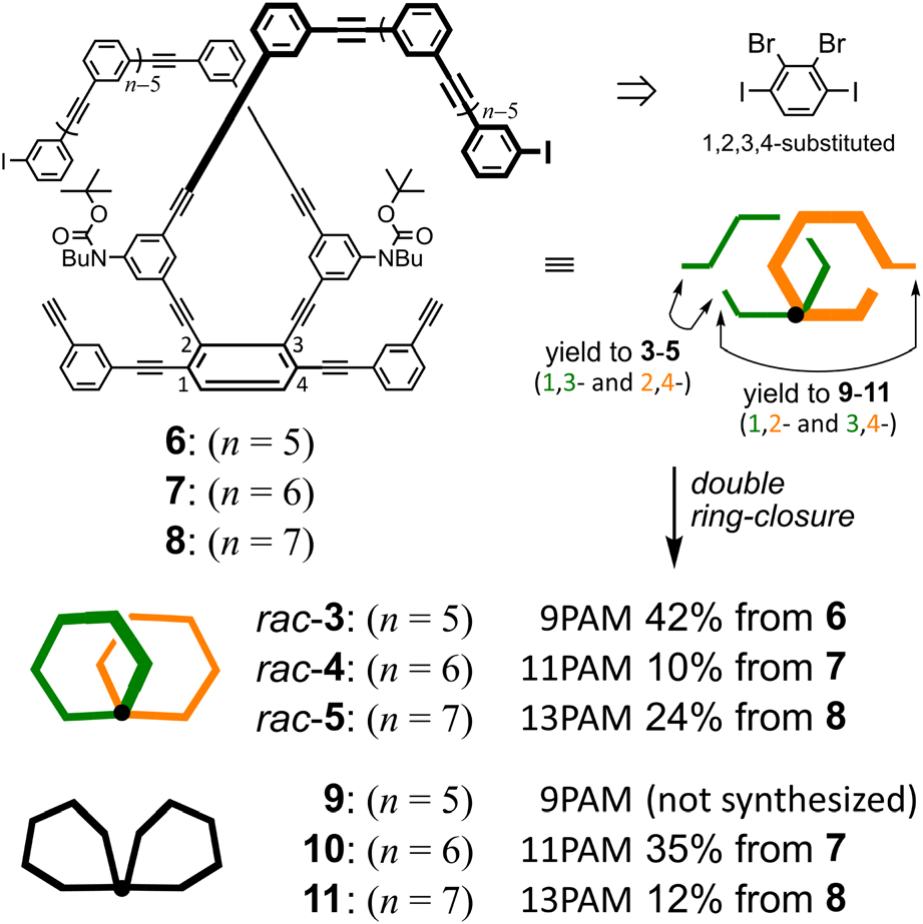
Synthetic results of the double ring-closing reaction. Conditions: Pd(PPh_3_)_4_, CuI, THF, Et_3_N, 75 °C.

Two-ring chiral 3 (9PAM), 4 (11PAM) and 5 (13PAM) were synthesized by double ring-closing reactions of α-iodo-ω-ethynyl sequences^17^ in the corresponding precursors 6 (*n* = 5), 7 (*n* = 6) and 8 (*n* = 7), respectively, through Sonogashira coupling (Fig. 2 and Schemes S1 and S2†). To prepare the precursors based on 1,2,3,4-tetrakis(phenylethynyl)benzene,^24^ we started with 2,3-dibromo-1,4-diiodobenzene,^25^ which could be sufficiently available under modified conditions (Scheme S1†). Through the double ring-closure, two isomers would be produced with a difference in the ring-closing positions (1-ethynyl-3-iodo and 2-iodo-4-ethynyl give 3–5, and 1-ethynyl-2-iodo and 3-iodo-4-ethynyl give 9–11; the number attached to α-iodo-ω-ethynyl sequences denotes the position with respect to the fused point for descriptive purposes). These two-ring isomers could be separable based on the difference in molecular polarity, which is due to the carbamate attached to the hydrocarbon. Two-ring chiral 3–5 are composed of a two-fold all-*meta* PAM, and would be obtained as racemates. Two-ring 9–11 are composed of a two-fold partially-*ortho* PAM, and would be obtained as an optically-inactive species, even though it could adopt some chiral conformation, since the relative occupation of each ring would be conformationally interconvertible.

**Fig. 3:**
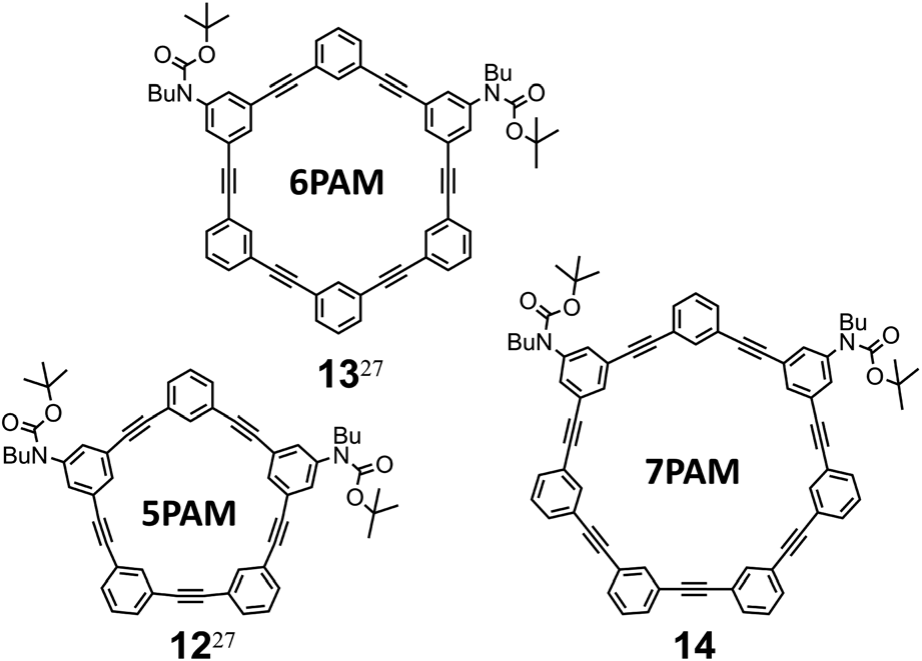
Chemical structures of all-*meta* single ring of PAMs.

Through the formation of a two-fold 6PAM with 7 (*n* = 6), as expected, two products were obtained and separated due to the difference in the *R*_f_ value on SiO_2_ (ref. 26) as well as the retention time in GPC. The mass spectra of the two isolated products showed an identical value (*m*/*z* = 1464.6). The assignment of these two isomeric products 4 and 10 was not based solely on differences in NMR and UV spectra (described later); the result regarding its passage through a chiral column was a determining factor ([*α*]_D_ = +448 for (+)-4 and −439 for (–)-4). Similar synthetic results were obtained with 8 (*n* = 7). As with *rac*-4 and 10, two isomeric products *rac*-5 and 11 (*m*/*z* = 1664.6) were separated and assigned due to the differences in their *R*_f_ values,^26^ retention time in GPC, and the result regarding its passage through a chiral column ([*α*]_D_ = +391 for (+)-5 and −373 for (–)-5). Alternatively, the double ring-closing reaction of 6 (*n* = 5) yielded a single product of either two-ring structure (*m*/*z* = 1264.5). Based on similarities in its NMR and UV spectra, compared to *rac*-4 (11PAM) or *rac*-5 (13PAM), the single product was assigned to *rac*-3, not to 9, although unfortunately we could not yet find any condition suitable for optical resolution of the racemate. The molecular structure and spectroscopic properties of a series of all-*meta* two-ring chiral 3, 4 and 5 are described below along with each corresponding all-*meta* single ring of 5PAM (12),^27^ 6PAM (13),^27^ and 7PAM (14) (Fig. 3 and Scheme S3†).

### Molecular structure of two-ring 3–5 and 10–11

**Fig. 4:**
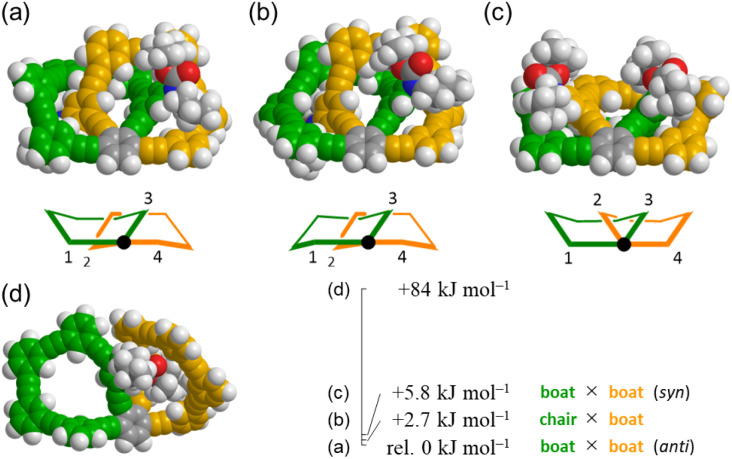
Energy-minimized structures (space-filling representation) for (*M*)-4 (11PAM): (a) rel. 0 kJ mol^−1^ (boat × boat, *anti*), (b) +2.7 kJ mol^−1^ (chair × boat), (c) +5.8 kJ mol^−1^ (boat × boat, *syn*), and (d) 10 (11PAM) (+84 kJ mol^−1^), obtained by conformational searches using MacroModel software (v11.8 OPLS3e, Monte Carlo Multiple Minimum method, non-solvated, 10 000 steps).

Conformational searches predicted energy-minimized structures for two 11PAMs (*n* = 6) of all-*meta*4 and partially-*ortho*10, as shown in Fig. 4. A search for 4 found that both rings of 6PAM adopted a nonplanar conformation to form (a) boat × boat, *anti*, (b) boat × chair [+2.7 kJ mol^−1^ relative to (a)], and (c) boat × boat, *syn* [+5.8 kJ mol^−1^ relative to (a)]. These three nonplanar structures would conformationally interconvert to each other through a partial flipping of either ring. In either conformation, the single-helix structure continued to exist even with some difference in local conformation; two phenylene groups at the 2- and 3-positions were helically-tilted in the same direction with respect to the central phenylene group (fused point) in conformations (a) and (b), while they were tilted in opposite directions to cancel each local chirality in (c). For partially-*ortho*10, two rings were predicted to adopt variant nonplanar conformations from each other (d), and were found at a considerably higher energy level (+84 kJ mol^−1^), compared to (a), (b) and (c) in all-*meta*4.

Two peripheral phenylene groups at the 1- and 4-positions were coplanar with respect to the fused point in every conformation of 4, while larger dihedral angles were made for the corresponding peripheral phenylene groups in 10.

The single-helix structure was also found in 3 (9PAM) and 5 (13PAM) (Fig. S1†). Five-membered rings in 3 were found to adopt a highly planar conformation, compared to six-membered rings in 4. Nonplanar conformations^18,21^ were seen for seven-membered rings in both 5 and 11 (13PAM), similar to the situation in 4 and 10 (11PAM).

**Fig. 5:**
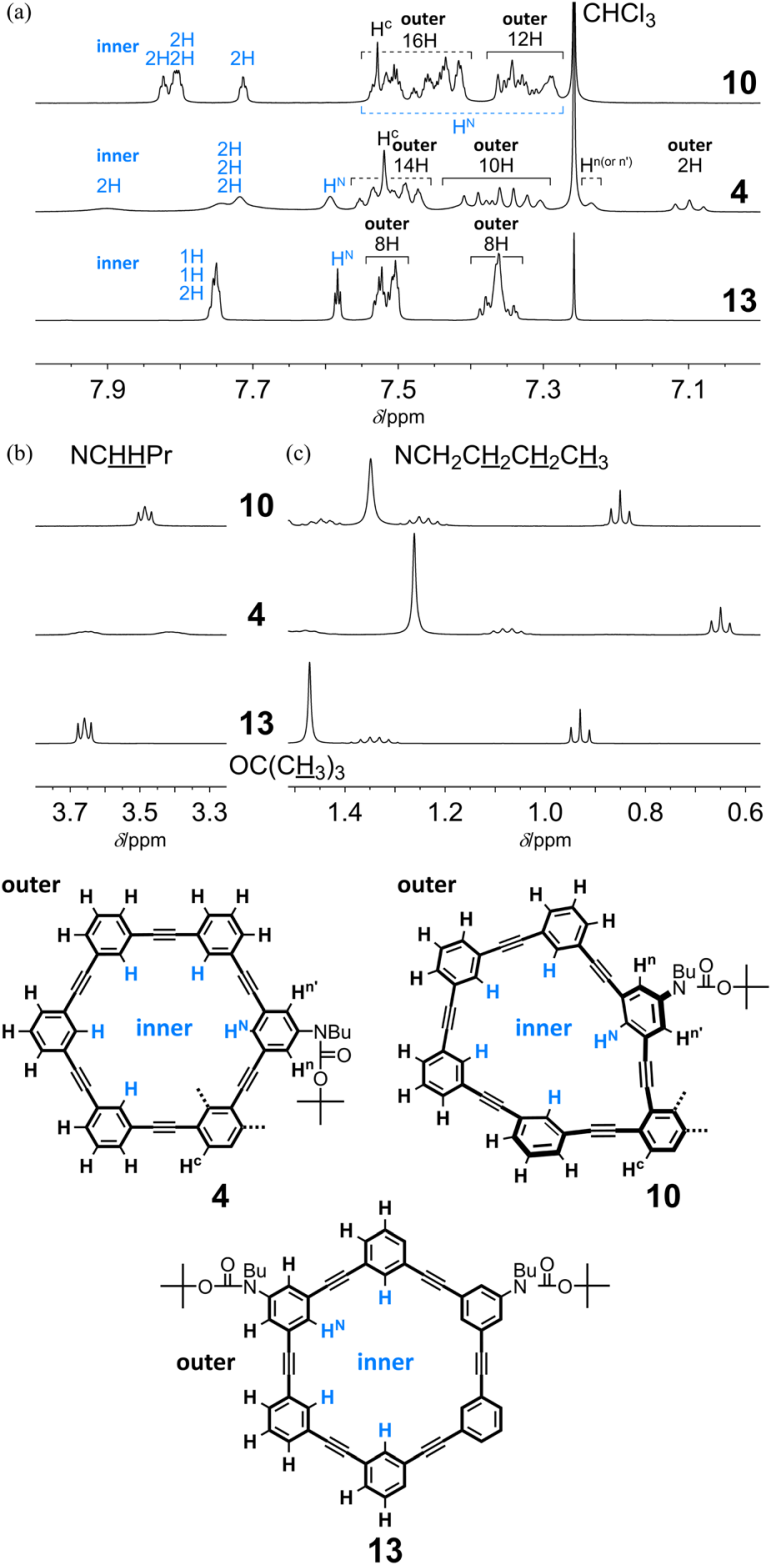
Partial ^1^H NMR spectra (400 MHz, (a) aromatic protons, (b) methylene protons closest to the nitrogen atom, and (c) aliphatic protons) of 4 (11PAM), 10 (11PAM) and 13 (6PAM), measured in chloroform-*d* at room temperature.

In the ^1^H NMR spectra of two-ring 11PAMs 4 and 10, measured at room temperature, a single set of averaged resonances was observed to show that the two rings were equivalent in solution (*C*_2_ for 4 and *C*_2v_ for 10) (Fig. 5). For two-ring 4, the molecular symmetry was changed from that of the single ring of 13, and all protons associated with both the inner and outer regions were differentiated regarding half of the molecule. In the spectrum of all-*meta*4, we found several remarkable changes in the chemical shift, compared to the corresponding protons in 13. Signals for a particular one of the outer protons and H^*n*(or *n*′)^ were shifted upfield (7.10 ppm and 7.24 ppm, respectively) due to shielding from some aromatic ring, and a signal for one of the inner protons was shifted downfield (7.90 ppm) due to deshielding from some triple bond. For the remaining inner protons, the chemical shift was almost unchanged even with the difference in molecular symmetry. These results showed that either 6PAM was partially stacked above or below the neighboring PAM. Alternatively, in the spectrum of partially-*ortho*10, no remarkable change was induced for any signal, as seen for 4, but both the inner and outer protons were slightly scattered according to the differentiation of the ring, compared to 13 (Fig. 5). In the aliphatic region, several signals were shifted upfield due to shielding from some aromatic ring(s) in the neighboring PAM for butyl (especially for protons farthest from the nitrogen) and *tert*-butyl protons in either spectrum of 4 or 10, in varying degrees.

In the spectrum of all-*meta*5 (13PAM), we observed the same upfield shift as seen for 4 (11PAM) of a signal for one of the outer protons (7.09 ppm) (Fig. S2A†). Similar upfield shifts were observed for a series of aliphatic protons, although the degree was less than that for 4 (11PAM). The spectrum of partially-*ortho*11 (13PAM) resembled that of 10 (11PAM) (Fig. S2B†). In the spectrum of all-*meta*3, obtained as a single product of 9PAM, there was no upfield shift for a particular outer proton as seen for 4 and 5 (Fig. S2A†). Instead, the signal for H^*n*(or *n*′)^ was shifted upfield (7.21 ppm), as seen in the spectrum of 4. Several signals, not only for inner protons but also for outer protons, were shifted downfield. These results could support the assignment to all-*meta*3, where either 5PAM could be partially stacked above or below the neighboring PAM.

The spectrum of 4, measured at 223 K, indicated the coexistence of multiple species with *C*_2_- as well as *C*_1_-symmetry (Fig. S3a†). These conformational isomers could convert to each other with an increase in temperature, to show a single set of averaged resonances above the coalescence temperature. The VT spectra of 10, measured at 223–323 K, revealed the nonequivalency in the two-ring structure (Fig. S3b†). A pair of singlet signals for *tert*-butyl protons were dominant at lower temperatures, and they coalesced above 263 K to show a single peak while maintaining the chemical shift. This result showed that a species with two nonequivalent rings that varied in conformation was dominant in solution. The averaging process upon changes in temperature was explained by conformational interconversion between each ring in a single dominant species.

**Fig. 6:**
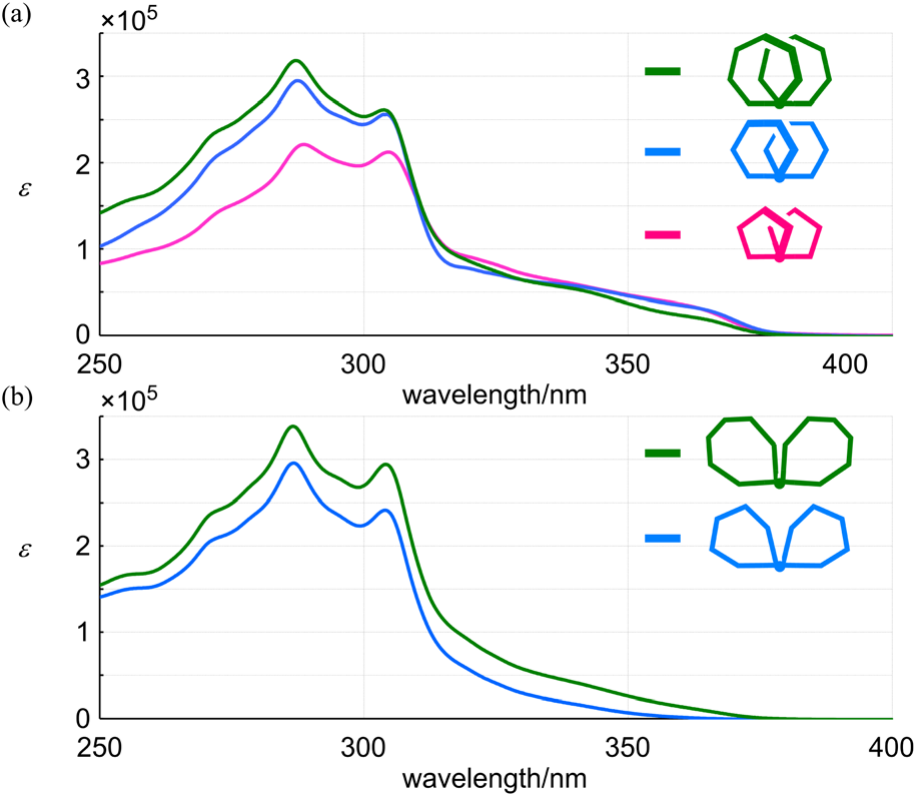
UV spectra of (a) all-*meta rac*-3 (9PAM, pink), *rac*-4 (11PAM, blue) and *rac*-5 (13PAM, green), and (b) partially-*ortho*10 (11PAM, blue) and 11 (13PAM, green), measured in dichloromethane at room temperature.

The absorption spectra of all-*meta*3, 4 and 5 shared a similar profile (Fig. 6a). In a shorter-wavelength region, the intensity increased with an increase in the number of phenylacetylene units from 9PAM to 11PAM and 13PAM. Alternatively, in a longer-wavelength region, the intensity was comparable for 9PAM and 11PAM, and attenuated for 13PAM. This finding could be related to the coplanarity of two peripheral phenylene rings at the 1- and 4-positions with respect to the fused point. In the spectra of partially-*ortho*10 and 11 (Fig. 6b), a similar profile was shown throughout the absorption region, and the intensity increased in proportion to the number of phenylacetylene units.

**Fig. 7:**
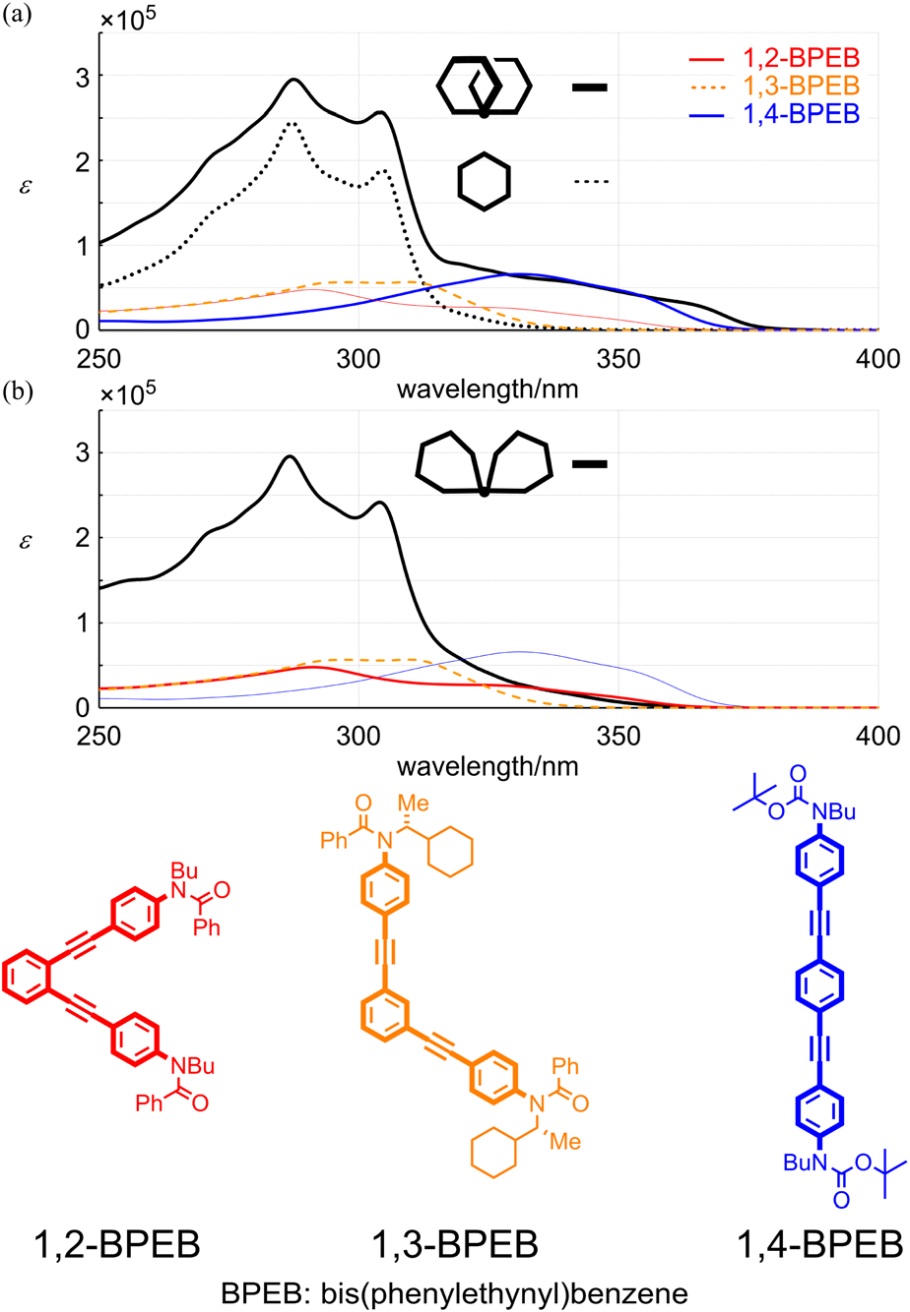
UV spectra of (a) *rac*-4 (11PAM, black thick solid line), 13 (6PAM, black thin dashed line), 1,2-BPEB (red line), 1,3-BPEB (orange line) and 1,4-BPEB (blue line), and (b) 10 (11PAM, black line), 1,2-BPEB (red line), 1,3-BPEB (orange line) and 1,4-BPEB (blue line). All spectra were measured in dichloromethane at room temperature.

Next, we compared the absorption properties of two-ring 11PAMs with those of the component element 6PAM and substructures 1,2-,^28^ 1,3- (ref. 29) and 1,4- (ref. 27) bis(phenylethynyl)benzenes (BPEBs) (Fig. 7 and Table 1). The absorption spectrum of 4 had two sections; one about *m*-phenylene ethynylenes, which resembled in appearance that of 6PAM,^16^ rather than 1,3-BPEB. The other absorption emerged in a longer wavelength region, which did not exist in the spectrum of 6PAM, and rather resembled that of 1,4-BPEB. The spectrum of 10 also had two sections and appeared to be composed of 6PAM and 1,2-BPEB. Regarding the intensity in a shorter-wavelength region about PAM, the sum of molar absorption coefficients (*ε*_1_ + *ε*_2_) for all-*meta*4 (11PAM) was 1.27-fold greater than that for 13 (6PAM). This value was smaller than 1.83 (=11 units/6 units, multiple of the number of phenylacetylene units), and rather close to 1.33 (=8/6). This result indicated that eight out of 11 units in 4 could exist as for PAM, and the remaining three units created new absorption similar to that of 1,4-BPEB. The sum of *ε*_1_ + *ε*_2_ for partially-*ortho*10 (11PAM) was 1.24-fold increased from that of 13 (6PAM), and new absorption that resembled that of 1,2-BPEB emerged in a longer wavelength region. A similar two-section absorption was seen for 3 (9PAM), and the relationship to 12 (5PAM) was similarly explained by the sum of *ε*_1_ + *ε*_2_. The value for 3 was 1.16-fold greater than that of 12, which was closer to 1.20 (=6/5) rather than 1.80 (=9 units/5 units).^30^

**Table 1:** Wavelengths (*λ*, *λ*_1_ and *λ*_2_) [nm] of absorption maximum, and molar absorption coefficients (*ε*, *ε*_1_ and *ε*_2_) [L mol^−1^ cm^−1^], measured in dichloromethane at room temperature

	*λ*	*ε*/10^4^	*λ*_1_	*ε*_1_/10^5^	*λ*_2_	*ε*_2_/10^5^
1 (6PAM × 2)	—	—	304	2.81	287	4.15
2 (6PAM × 2)	—	—	Sh. 306	2.14	288	3.72
3 (all-*meta* 9PAM)	Sh. 362, sh. 340, sh. 322	3.36, 5.90, 8.71	305	2.12	289	2.21
4 (all-*meta* 11PAM)	Sh. 363, sh. 340, sh. 320	3.19, 5.73, 7.72	304	2.56	287	2.94
5 (all-*meta* 13PAM)	Sh. 363, sh. 342, sh. 324	2.07, 5.08, 7.62	304	2.61	287	3.18
12 (5PAM)	—	—	304	1.59	287	2.16
13 (6PAM)	—	—	305	1.88	287	2.45
10 (partially-*ortho* 11PAM)	Sh. 351^a^, sh. 331^a^	0.61, 2.76	304	2.41	287	2.95
11 (partially-*ortho* 13PAM)	Sh. 351^a^, sh. 331^a^	2.45, 5.56	304	2.94	287	3.38
1,2-BPEB	Sh. 351^a^, sh. 331^a^	1.14, 2.49	—	—	291	0.478
1,3-BPEB	—	—	309	0.567	298	0.565
1,4-BPEB	Sh. 352, 331	4.45, 6.59	—	—	—	—

a Since there was no distinct extreme at a longer wavelength region in the spectra of 10, 11 and 1,2-BPEB, for such shoulder (sh.) peaks, *ε* values at the same wavelength are noted for reference.

### Chiroptical properties

**Fig. 8:**
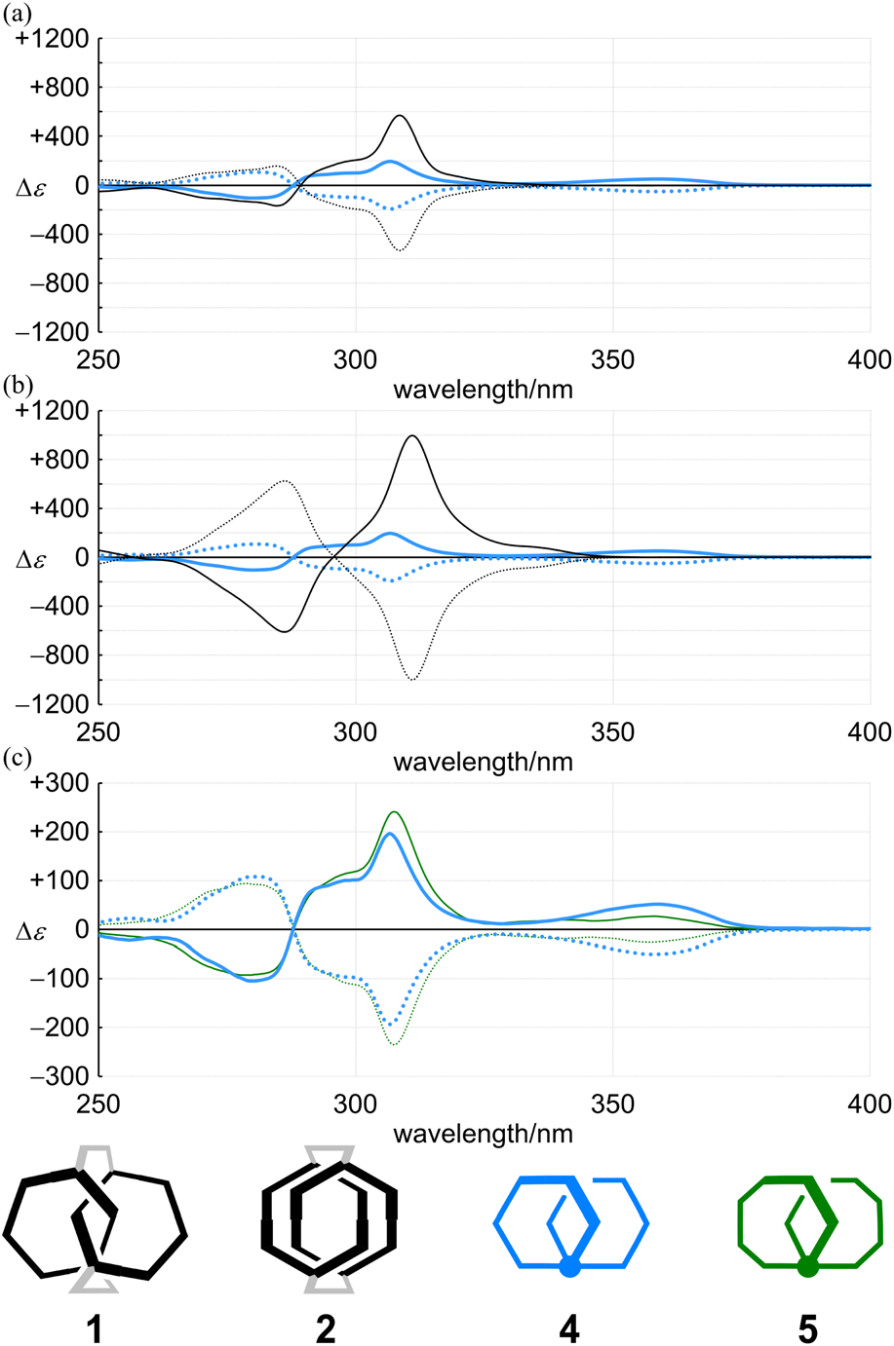
CD spectra of (a) (+)-1 (black solid line), (–)-1 (black dashed line), (+)-4 (blue solid line) and (–)-4 (blue dashed line), (b) (+)-2 (black solid line), (–)-2 (black dashed line), (+)-4 (blue solid line) and (–)-4 (blue dashed line), and (c) (+)-4 (blue solid line), (–)-4 (blue dashed line), (+)-5 (green solid line) and (–)-5 (green dashed line). All spectra were measured in dichloromethane at 293 K.

The racemates 4 (11PAM) and 5 (13PAM) were optically resolved by HPLC with a chiral stationary column. We found compositive Cotton effects for each enantiomer in the two-section absorption region (Fig. 8). Although the extreme wavelengths (*λ*_1_ and *λ*_2_) in the absorption spectra of 1, 2 and 4 were consistent (Table 1), the extreme wavelengths in the CD spectra were slightly different. Therefore, we compared the molar CDs (Δ*ε*) at extreme wavelengths in the CD spectra of 1 (6PAM × 2) and 2 (6PAM × 2) to those of 4 (11PAM).

**Fig. 9:**
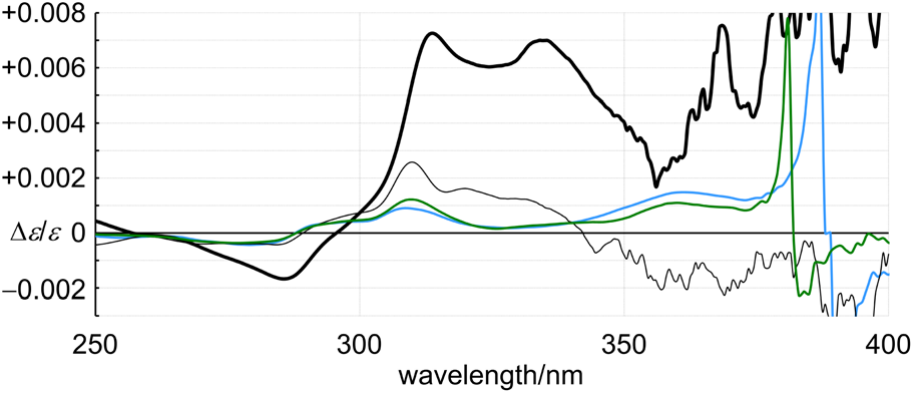
Plots of Δ*ε*/*ε* for (+)-1 (6PAM × 2, black thin solid line), (+)-2 (6PAM × 2, black thick solid line), (+)-4 (11PAM, blue line) and (+)-5 (13PAM, green line) *versus* wavelength. All spectra were measured in dichloromethane at room temperature.

In the comparison of 1 and 4 (Fig. 8a), regarding the absorption region about PAM, the spectral appearance was comparable. The values of *ε*_1_ and Δ*ε*_1_ for 1 were increased by 1.0- and 2.9-fold, and *ε*_2_ and Δ*ε*_2_ were each increased by 1.6-fold, respectively. In the comparison of 2 and 4 (Fig. 8b), regarding the absorption region about PAM, the spectral appearance was different. The values of *ε*_1_ and Δ*ε*_1_ for 2 were increased by 0.67- and 5.1-fold, and *ε*_2_ and Δ*ε*_2_ were increased by 1.5- and 6.0-fold, respectively. In the spectrum of 2, unique Cotton effects were found at around 340 nm, which were absent from the spectra of 1 and 4. Thus, a similar pattern for the plots of Δ*ε*/*ε* was obtained with 1 and 4, while an original pattern was created by 2 (Fig. 9). These similarities and differences indicated that the double helix in 1 seemed to be close to the single helix in 4, and differed from the double helix in 2. For such a structure in 1, either ring was misaligned by reversal of the local chirality at one of the two bridges to adopt a conformation similar to the single helix in 4, while maintaining the global configuration. Through the alignment of two rings in 2, a successive arrangement of several local chiral environments was attained to create unique Cotton effects for the double helix generated by the mechanical linkage of the two 6PAMs, as well as for local chirality generated by the proximity of each peripheral side of 6PAM.

**Fig. 10:**
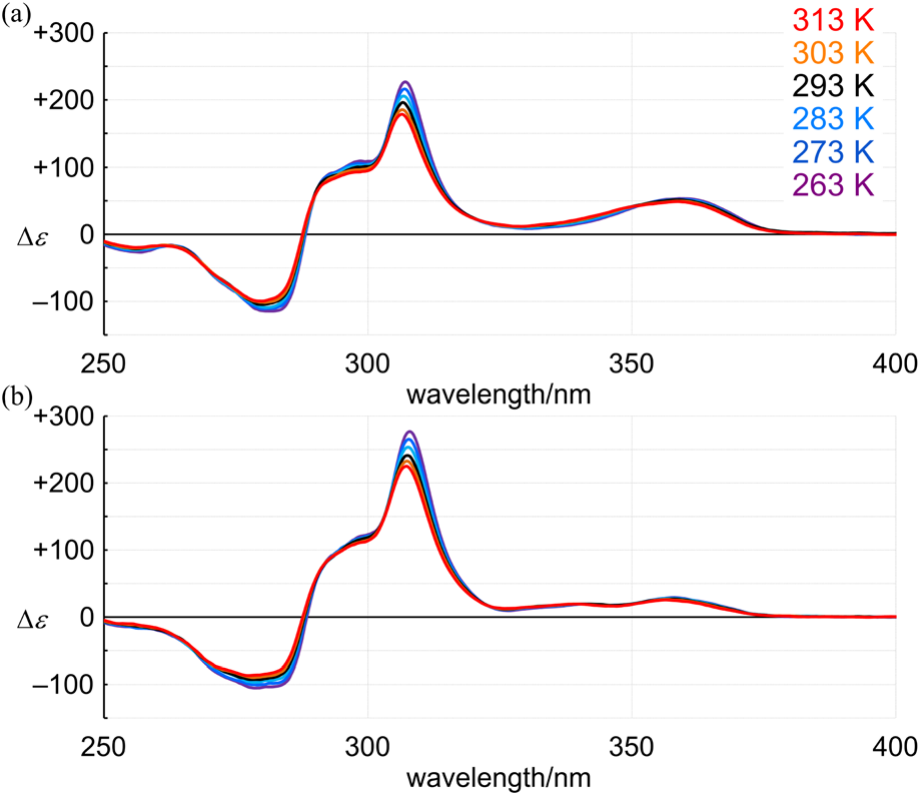
VT-CD spectra of (a) (+)-4 (11PAM) and (b) (+)-5 (13PAM), measured in dichloromethane at 263–313 K.

In the CD spectra of 4 (11PAM) and 5 (13PAM), we found an identical profile of the Cotton effects throughout the two-section absorption region (Fig. 8c). Regarding the absorption region about PAM, the intensity of molar CDs for 5 was greater than that of 4. If we considered the consistence in the values of Δ*ε*/*ε* (Fig. 9), this increase in Δ*ε* was due to an increase in *ε*, and the single-helix structure in 4 and 5 was inherently identical. The Cotton effects in this region varied with temperature, the intensity was greater at lower temperatures, which was due to interconversions among nonplanar conformations of PAM (Fig. 10). Alternatively, in a longer wavelength region, the intensity of the Cotton effects was greater for 4, which was invariant with a change in temperature (Fig. 10). This result indicated that the Cotton effects in this region were inherently generated in 4 and 5 even with a slight difference in the two phenylene ethynylenes at the 1- and 4-positions with respect to the fused point.

**Fig. 11:**
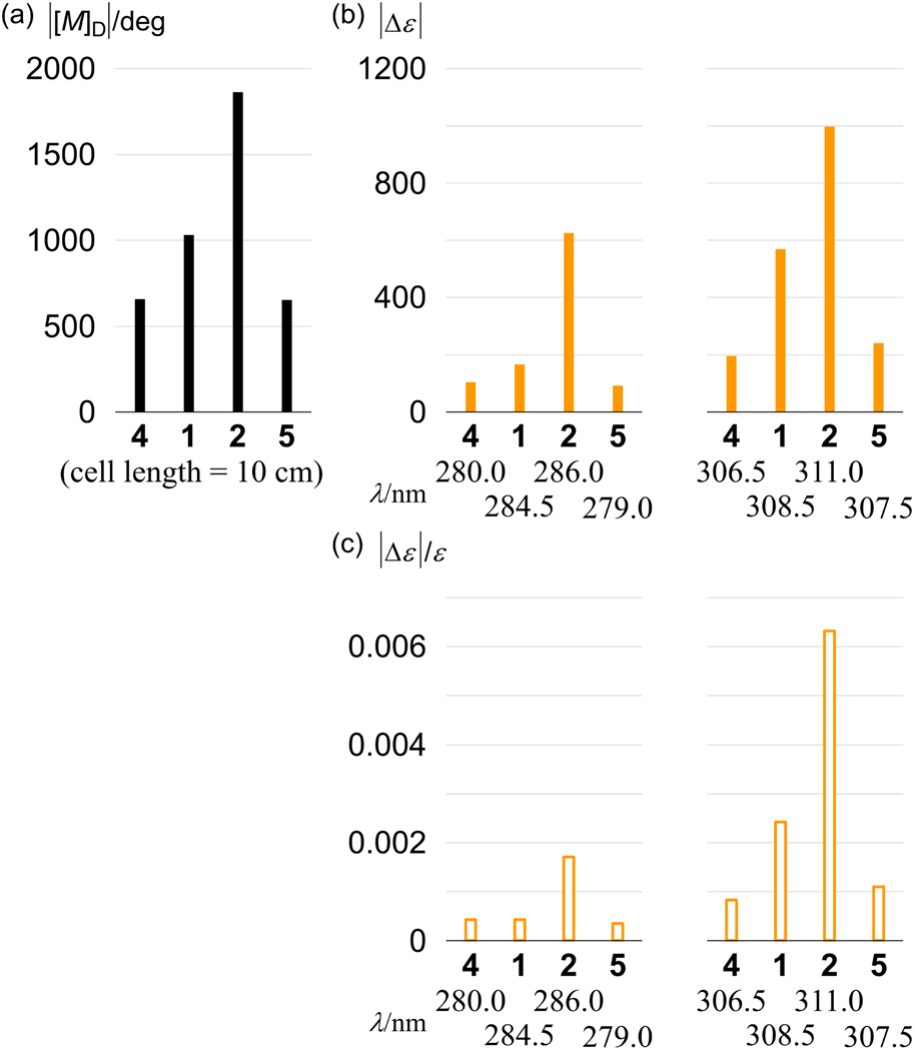
Absolute values of (a) molar optical rotation [*M*]_D_ (1 M, cell length = 10 cm) of (+)-4 ([*α*]_D_ = +448), (+)-1 ([*α*]_D_ = +591), (–)-2 ([*α*]_D_ = −1067) and (+)-5 ([*α*]_D_ = +391), measured in chloroform at room temperature, (b) molar circular dichroism and (c) dissymmetry at extreme wavelengths, measured in dichloromethane at 293 K.

Finally, we compared the absolute values of molar optical rotation [*M*]_D_ for 1, 2, 4 and 5. The values increased for 4 (657), 1 (1031) and 2 (1863) in this order (Fig. 11). We found that this increasing pattern was similar to that of the molar CD and that of dissymmetry in the absorption region about PAM. In the comparison of 4 and 5 with the identical single-helix structure, the value for 5 (651) was almost the same as that for 4.

## Conclusions

We have used two achiral rings of 6PAM to create chiral molecules (1 and 2) and demonstrated that the chiroptical properties were uniquely generated in each assembly (6PAM × 2). To explain this disparity, we were interested in a double-helix structure generated in 1 and 2, and synthesized a two-ring chiral analog with identical components by the ring fusion of two 6PAMs to 11PAM (4), where the single helix exists as a chiral source in place of the double helix in 1 and 2.

In the comparison of the absorption properties of the two rings in 1 and 2 with those of two-ring 4, they shared a similar profile in each spectrum due to the identical component. The CD spectrum of 1 was similar in appearance to that of 4. Alternatively, the CD spectrum of 2 was different from those of 1 and 4 with the generation of unique Cotton effects. Based on the similarities and differences, we assumed that the double helix in 1 was deformed to adopt a conformation similar to the single helix in 4, while the double helix in 2 provided a unique chiral environment through the alignment of two rings. The value of the molar optical rotation was greater for 2 than for 1 (and 4).

In the comparison with two-ring analog 5 (13PAM), which was synthesized by ring fusion of two 7PAMs for reference, we found that the molar absorption coefficient and the molar circular dichroism increased with the number of phenylacetylene units. Thus, 4 (11PAM) and 5 (13PAM) showed the same profile in their dissymmetry based on the identical single helix.

## Conflicts of interest

There are no conflicts to declare.

## Supplementary Material

RA-013-D3RA01780J-s001
