# Supplementary material for: Two-ring chirality generated by the alignment of two achiral phenylacetylene macrocycles
Source: RSC Adv. 2023 Apr 14;13(17):11712–9. doi: 10.1039/d3ra01780j (PMC10102884; doi:10.1039/d3ra01780j)
Supplement: RA-013-D3RA01780J-s001 [file RA-013-D3RA01780J-s001.pdf]

Electronic Supplementary Material (ESI) for RSC Advances.
This journal is © The Royal Society of Chemistry 2023

Supplementary Information

Two-ring chirality generated by the alignment of two achiral phenylacetylene macrocycles

Ryo Katoono* and Kohei Arisawa

Department of Chemistry, Faculty of Science, Hokkaido University

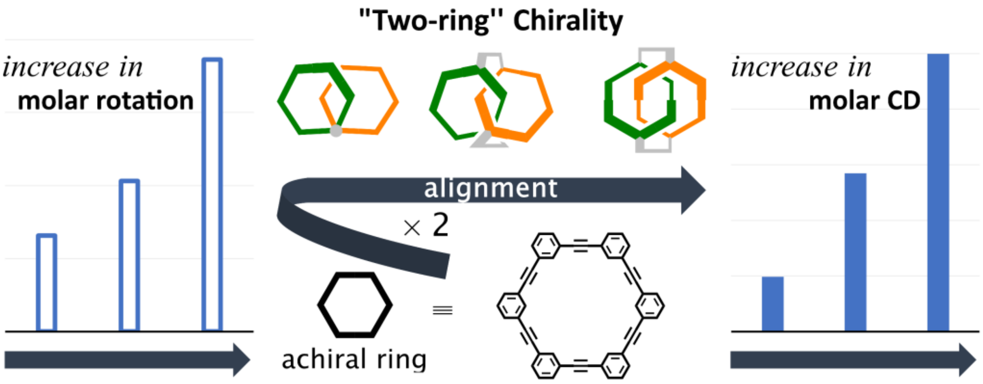

Contents

Supplementary Figures (**Figures S1-S3**)	S2-S7
Experimental (**Schemes S1-S3**)	S8-S20
References	S20
Copies of ^{1}H/^{13}C NMR and MS spectra of new compounds	S21-S48

Supplementary Figures

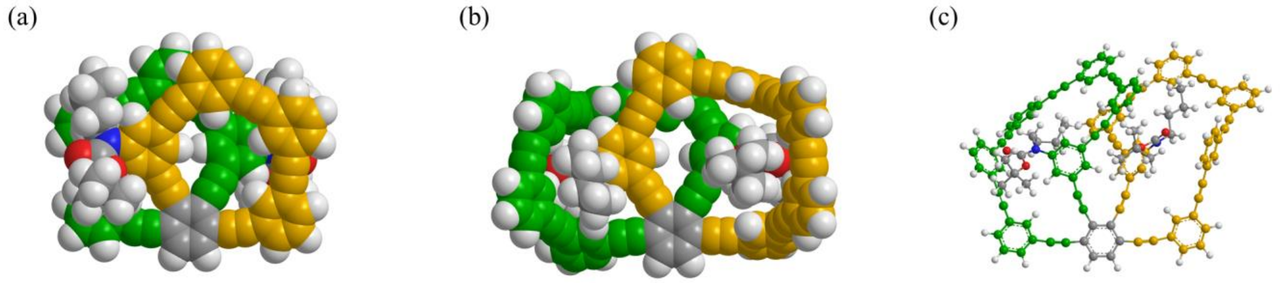

Fig. S1 The lowest energy structures for (a) (*M*)-**3** (9PAM), (b) (*M*)-**5** (13PAM) (rel. 0 kJ mol^{-1}) (space-filling representation), and (c) **11** (13PAM) (+34.6 kJ mol^{-1}) (ball and stick representation), obtained by conformational searches using MacroModel software (v11.8 OPLS3e, Monte Carlo Multiple Minimum method, non-solvated, 10 000 steps). Only one enantiomer is depicted for two-ring chiral **3** and **5**.

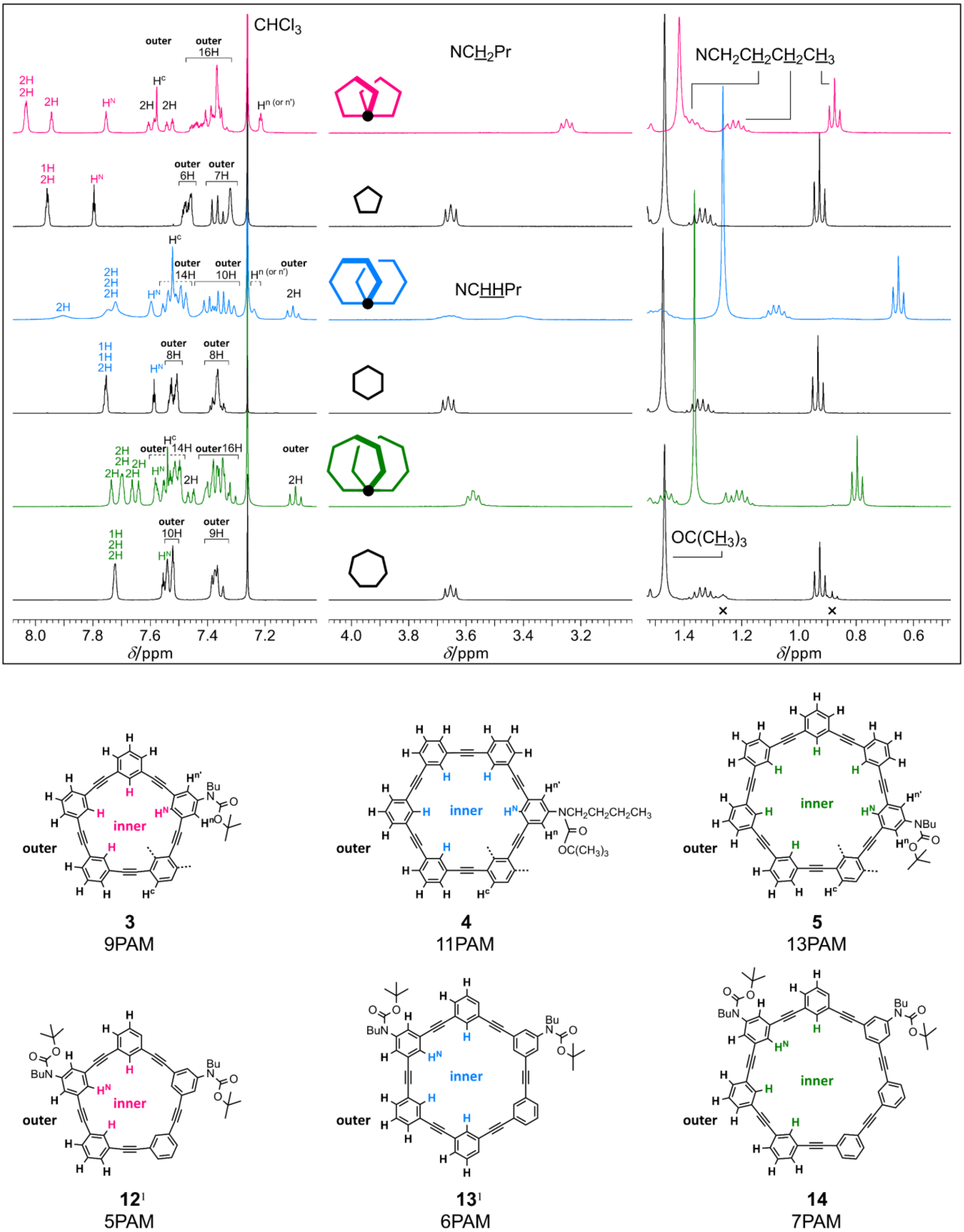

Fig. S2A Partial ^{1}H NMR spectra (400 MHz; left: aromatic protons, middle: methylene protons, closest to the nitrogen atom, and right: aliphatic protons) of *rac*-**3** (9PAM), **12** (5PAM), *rac*-**4** (11PAM), **13** (6PAM), *rac*-**5** (13PAM) and **14** (7PAM), measured in chloroform-*d* at room temperature.

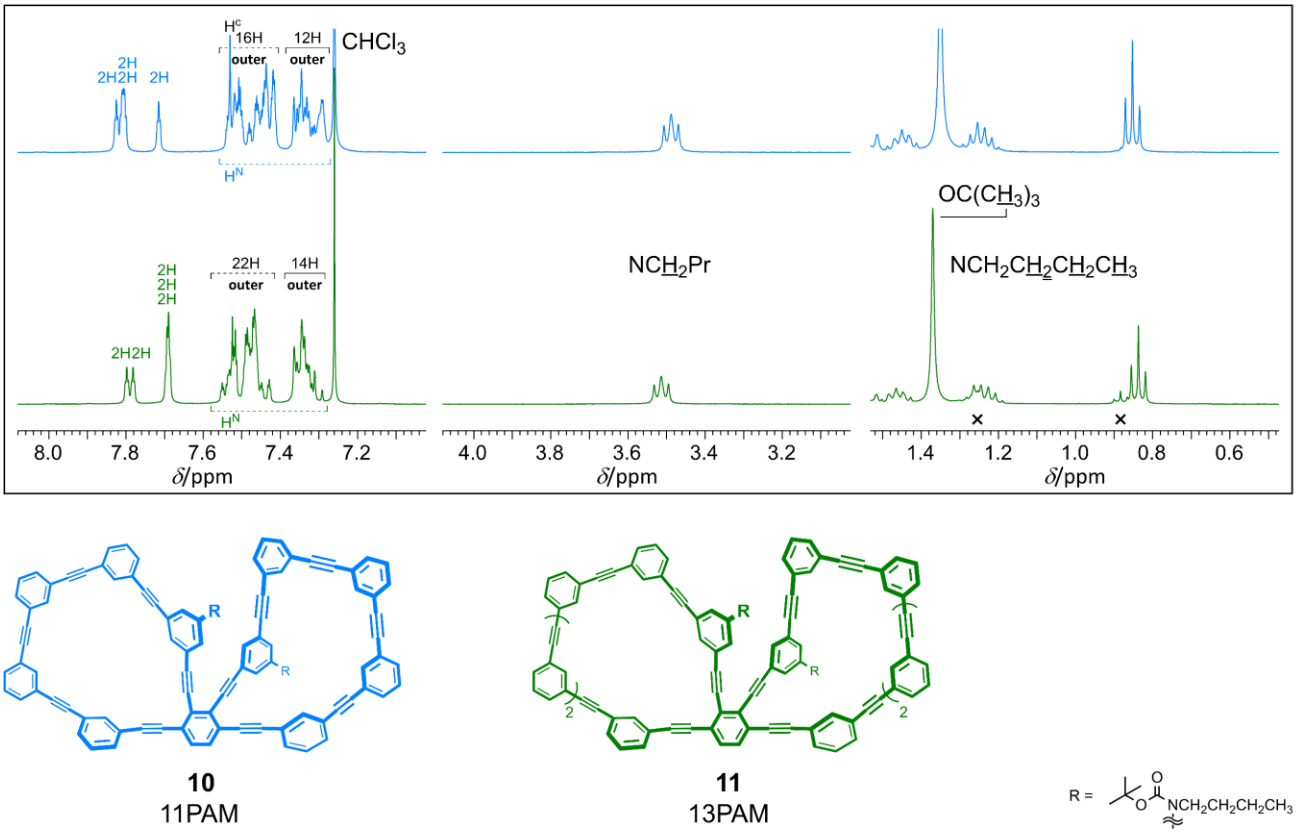

Fig. S2B Partial ^{1}H NMR spectra (400 MHz; left: aromatic protons, middle: methylene protons, closest to the nitrogen atom, and right: aliphatic protons) of **10** (11PAM) and **11** (13PAM), measured in chloroform-*d* at room temperature.

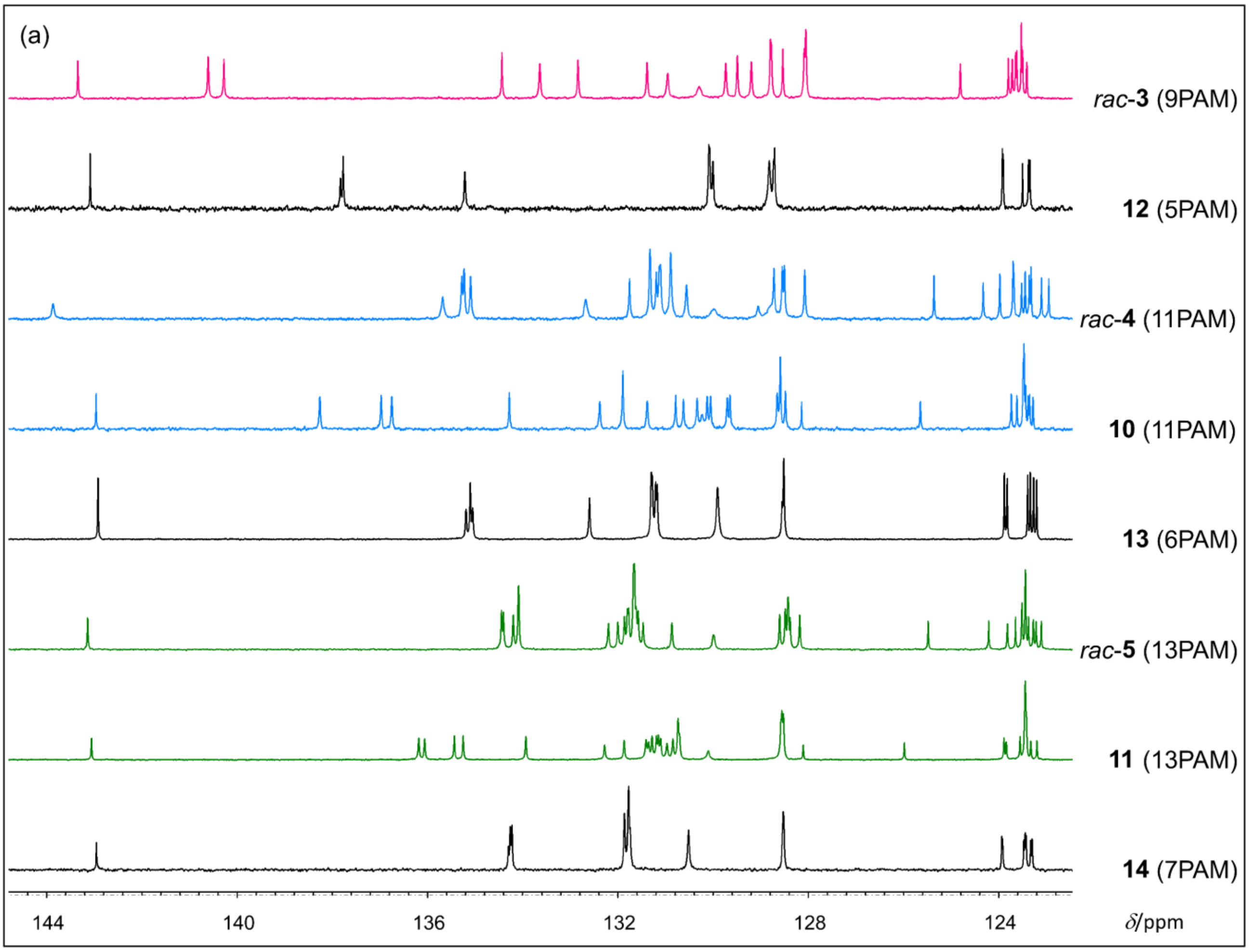

Fig. S2C Partial ^{13}C NMR spectra [100 MHz; (a): aromatic carbons] of *rac*-**3** (9PAM), **12** (5PAM), *rac*-**4** (11PAM), **10** (11PAM), **13** (6PAM), *rac*-**5** (13PAM), **11** (13PAM) and **14** (7PAM), measured in chloroform-*d* at room temperature.

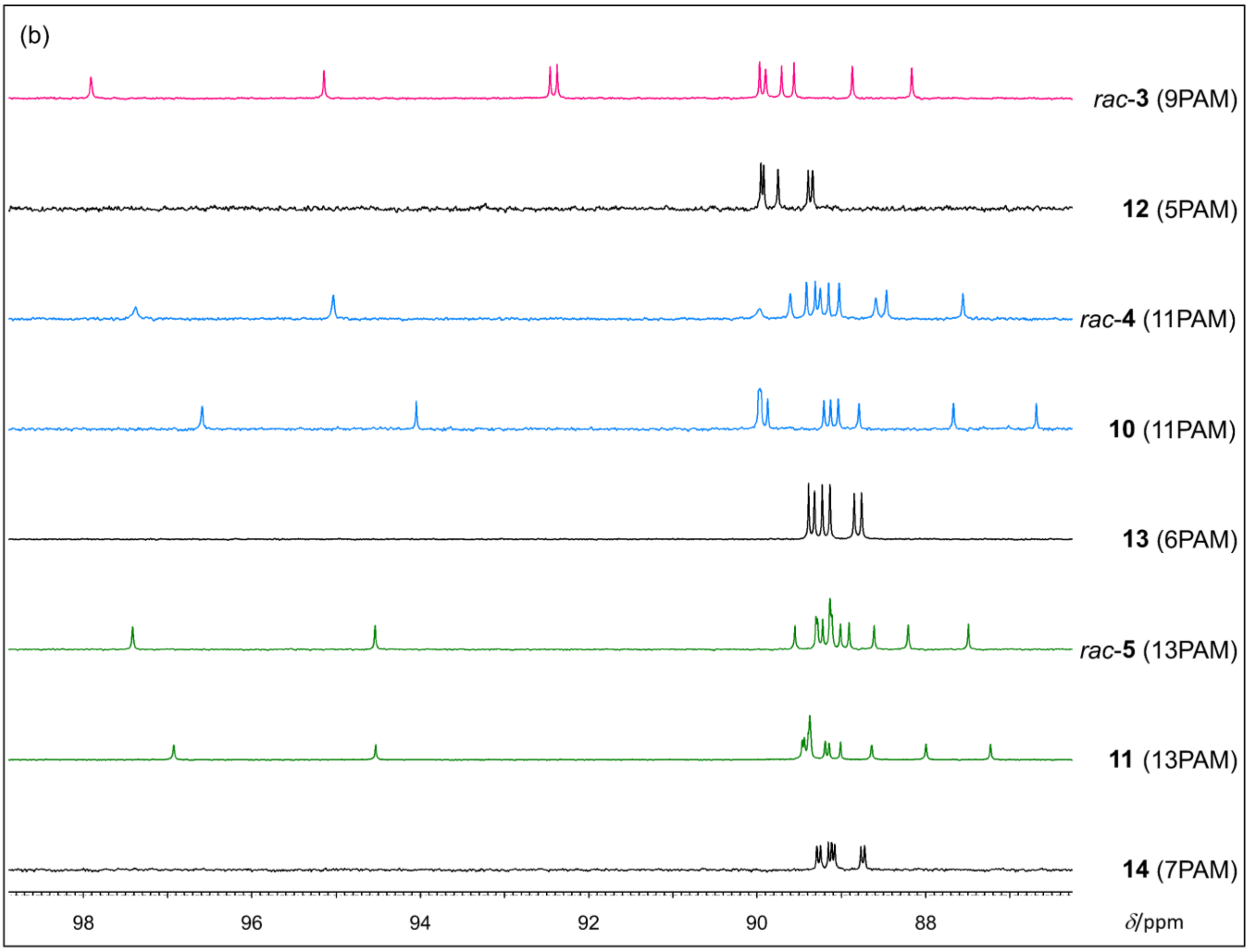

Fig. S2C Partial ^{13}C NMR spectra [100 MHz; (b): acetylenic carbons] of *rac*-**3** (9PAM), **12** (5PAM), *rac*-**4** (11PAM), **10** (11PAM), **13** (6PAM), *rac*-**5** (13PAM), **11** (13PAM) and **14** (7PAM), measured in chloroform-*d* at room temperature.

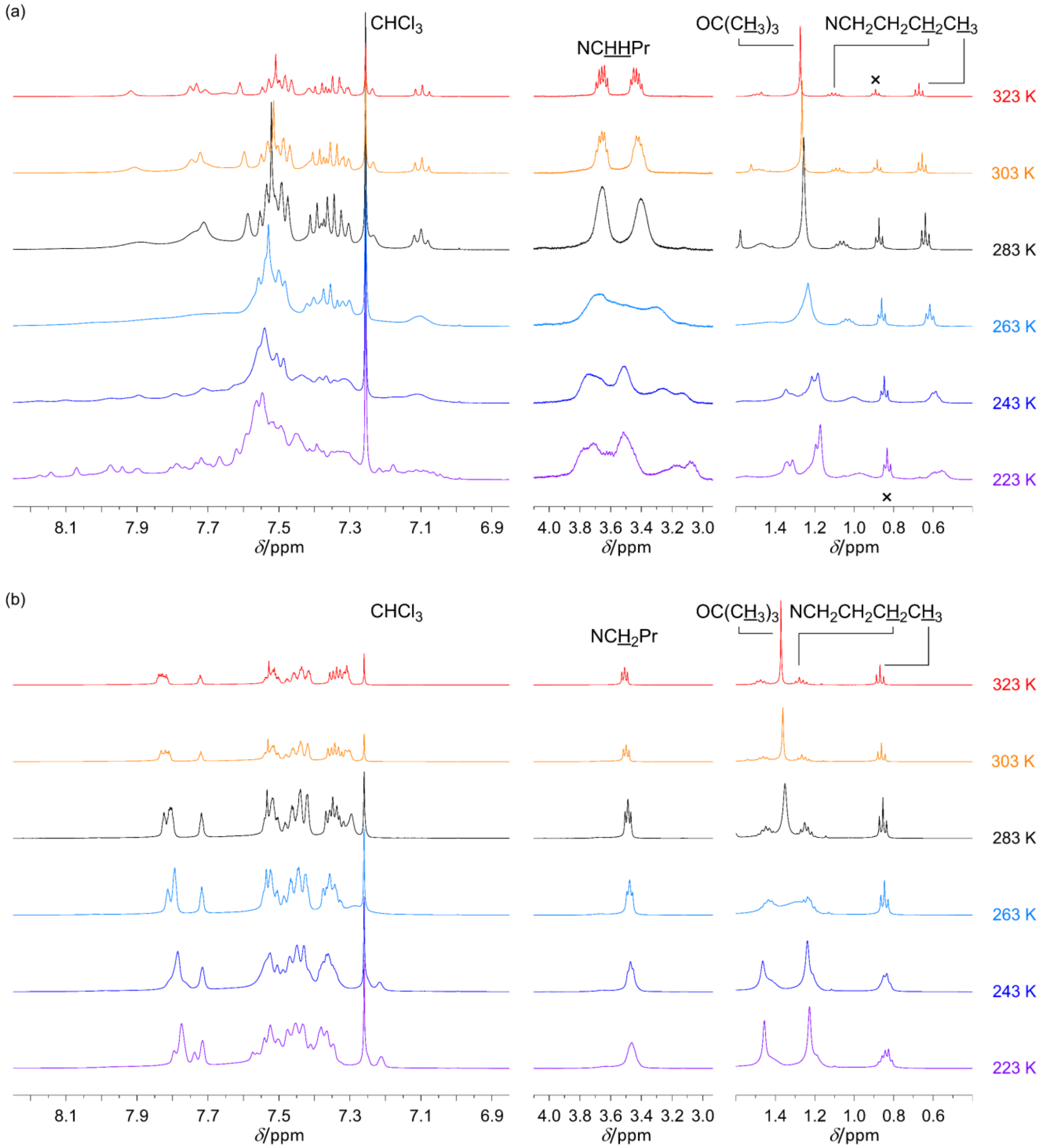

Fig. S3 Partial VT ^{1}H NMR spectra (400 MHz; left: aromatic protons, middle: methylene protons, closest to the nitrogen atom, and right: aliphatic protons) of 11PAMs: (a) **4** and (b) **10**, measured in chloroform-*d* at 223-323 K.

Experimental

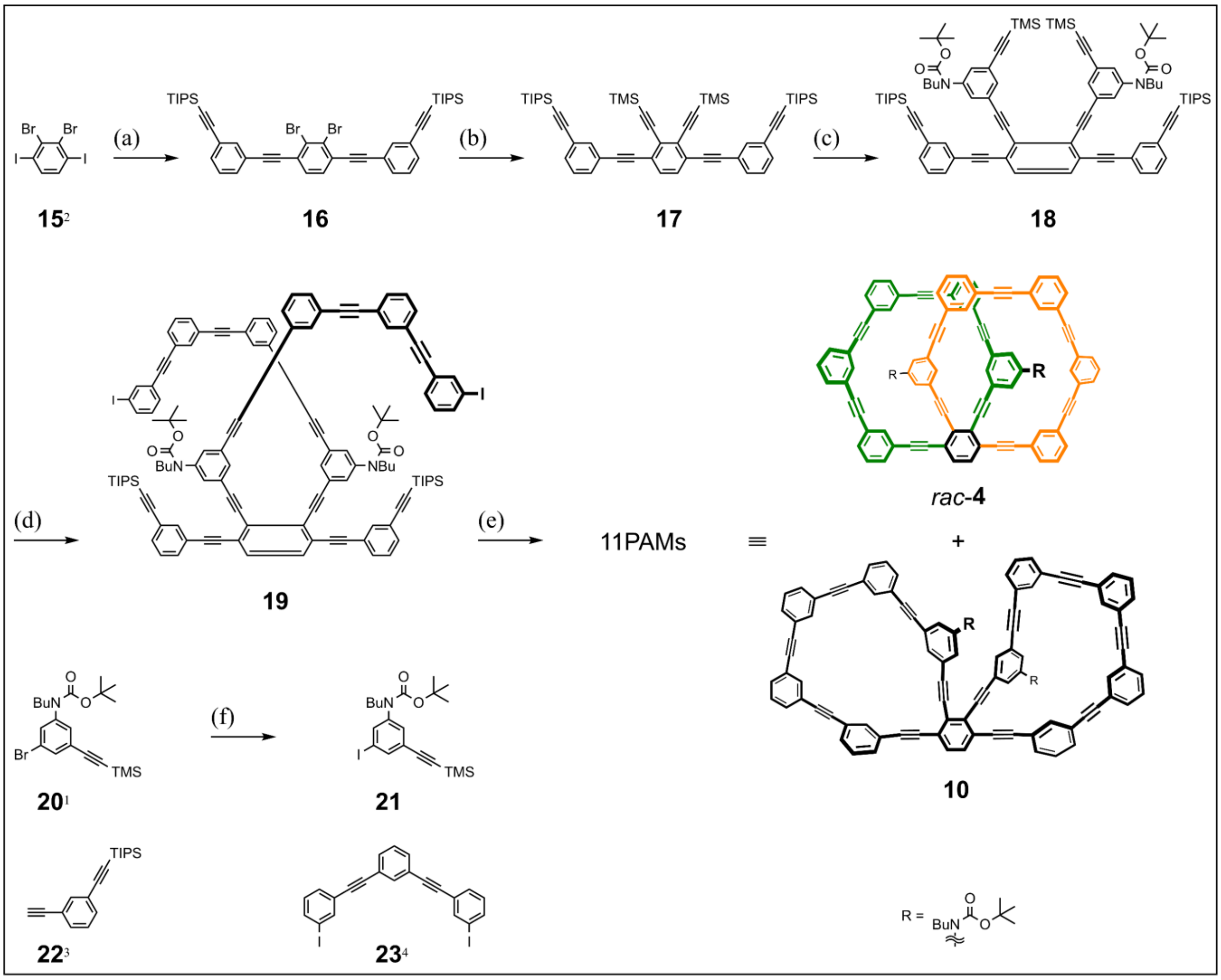

Scheme S1. Synthesis of 11PAMs, *rac*-**4** and **10**. Reagents and yields: (a) **22**, $Pd(PPh_3)_4$, CuI, Et_3N (92%); (b) TMSA, $Pd(PPh_3)_4$, CuI, Et_3N (80%); (c) i) K_2CO_3, MeOH, THF (96%), ii) **21**, $Pd(PPh_3)_4$, CuI, Et_3N, THF (94%); (d) i) NaH, MeOH, THF (97%), ii) **23**, $Pd(PPh_3)_4$, CuI, Et_3N, THF (52%); (e) i) tetra-^{n}butylammonium fluoride (TBAF), THF (78%), ii) $Pd(PPh_3)_4$, CuI, Et_3N, THF (10% for *rac*-**4** and 35% for **10**); (f) ^{n}BuLi, 1,2-diiodoethane, diethyl ether (98%).

Preparation of **15**[2] (*modified procedure*)

To a solution of 1,2-dibromobenzene (18.0 g, 76.3 mmol) and TMSCl (39 mL, 0.31 mol) in THF (214 mL) at –78 ºC was added a solution of LDA, which was prepared by $^{i}Pr_2NH$ (44 mL, 0.31 mol) and ^{n}BuLi (1.6 M in hexane, 194 mL, 305 mmol) in THF (175 mL) at –78 ºC and used after being warmed to room temperature, via a syringe pump (3 mL/min). The mixture was further stirred at –78 ºC for 17 h, quenched with aq. 1M HCl, and then diluted with hexane. The organic layer was separated, washed with brine, dried over magnesium sulfate, and then purified by column chromatography on SiO_2 (hexane) to give 1,4-bis(trimethylsilyl)-2,3-dibromobenzene (26.1 g) as a white solid in 90% yield.

To an ice-cooled solution of ICl (24.6 g, 151 mmol) in CH_2Cl_2 (220 mL) was added a solution of 1,4-bis(trimethylsilyl)-2,3-dibromobenzene (26.1 g, 68.7 mmol) in CH_2Cl_2 (54 mL), and the mixture was stirred at room temperature for 1 h, quenched with satd. aq. 1M $NaHCO_3$., and then separated. The organic layer was washed with

aq. NaOH, water and brine, dried over magnesium sulfate, and then purified by column chromatography on SiO_2 (chloroform), followed by recrystallization from 2-propanol to give **15** (30.1 g) as colorless needles in 90%.

Preparation of **16**

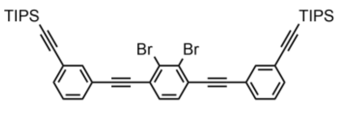

16

To a solution of **15** (1.71 g, 3.51 mmol) and **22**[3] (2.02 g, 7.16 mmol) in Et_3N (70 mL) were added $Pd(PPh_3)_4$ (122 mg, 0.106 mmol) and CuI (40 mg, 0.21 mmol) at room temperature under an argon atmosphere, and the mixture was stirred for 4 h. After removal of a solid by filtration through a Celite pad, the filtrate was concentrated and purified by column chromatography on SiO_2 (hexane) to give **16** (2.58 g, 92%). **16**: elemental analyses Found: C, 66.29; H, 6.64%. Calc. for $C_{44}H_{52}Br_2Si_2$: C, 66.32; H, 6.58%; ^{1}H NMR δ_H(400 MHz; $CDCl_3$; Me_4Si)/ppm 7.67 (2H, t, J = 1.2 Hz), 7.51 (2H, dt, J = 1.2, 7.6 Hz), 7.48 (2H, dt, J = 1.2, 8.0 Hz), 7.47 (2H, s), 7.32 (2H, dd, J = 7.6, 8.0 Hz), 1.3-1.1 (42H, br.m); ^{13}C NMR δ_C(100 MHz; $CDCl_3$)/ppm 135.0, 132.5, 131.5, 131.1, 128.7, 128.4, 126.9, 124.0, 122.6, 105.9, 95.1, 91.8, 88.8, 18.7, 11.3; FD-LRMS *m/z* 794.1 (M^+, 46%), 795.1 ($[M+1]^+$, 28), 796.1 ($[M+2]^+$, 100), 797.1 ($[M+3]^+$, 58), 798.1 ($[M+4]^+$, 69), 799.1 ($[M+5]^+$, 35), 800.1 ($[M+6]^+$, 15).

Preparation of **17**

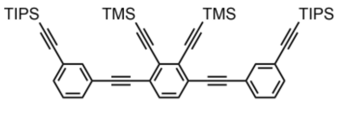

17

To a solution of **16** (2.00 g, 2.51 mmol), $Pd(PPh_3)_4$ (147 mg, 0.127 mmol) and CuI (48 mg, 0.25 mmol) in Et_3N (50 mL) was added TMSA (2.1 mL, 15 mmol) at 80 °C under an argon atmosphere, and the mixture was stirred for 6 h. After removal of a solid by filtration through a Celite pad, the filtrate was concentrated and purified by column chromatography on SiO_2 (1:8 dichloromethane/hexane) to give **17** (1.67 g, 80%). **17**: elemental analyses Found: C, 77.88; H, 8.64%. Calc. for $C_{54}H_{70}Si_4$: C, 78.00; H, 8.49%; ^{1}H NMR δ_H(400 MHz; $CDCl_3$; Me_4Si)/ppm 7.67 (2H, t, J = 1.6 Hz), 7.48 (2H, dt, J = 1.6, 8.0 Hz), 7.46 (2H, dt, J = 1.2, 8.0 Hz), 7.42 (2H, s), 7.30 (2H, dd, J = 7.6, 8.0 Hz), 1.2-1.1 (42H, br.m), 0.31 (18H, s); ^{13}C NMR δ_C(100 MHz; $CDCl_3$)/ppm 135.2, 132.2, 131.4, 130.8, 128.5, 128.3, 125.8, 123.9, 123.2, 105.9, 104.0, 101.3, 94.4, 91.5, 88.4, 18.7, 11.3, 0.1; FD-LRMS *m/z* 830.5 (M^+, 100%), 831.5 ($[M+1]^+$, 78), 832.5 ($[M+2]^+$, 47), 833.5 ($[M+3]^+$, 19).

Preparation of **18**

To a solution of **17** (2.41 g, 2.89 mmol) in THF (15 mL) and MeOH (15 mL) was added K_2CO_3 (1.18 g, 8.69 mmol) at room temperature, and the mixture was stirred at that temperature for 20 min. After addition of water, the organic layer was separated, washed with brine, dried over magnesium sulfate, and then purified by column chromatography on SiO_2 (1:5 dichloromethane/hexane) to give **17'** (1.91 g, 96%). **17'**: ^{1}H NMR δ_H(400 MHz; $CDCl_3$; Me_4Si)/ppm 7.67 (2H, t, J = 1.6 Hz), 7.51-7.49 (4H, m), 7.47 (2H, dt, J = 1.6, 8.0 Hz), 7.31 (2H, dd, J = 7.6, 8.0 Hz), 3.67 (2H, s), 1.2-1.1 (42H, br.m).

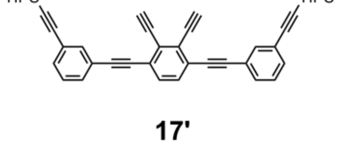

17'

To a solution of **21** (5.24 g, 11.1 mmol), $Pd(PPh_3)_4$ (321 mg, 0.278 mmol) and CuI (54 mg, 0.28 mmol) in Et_3N (15 mL) was added a solution of **17'** (1.91 g, 2.78 mmol) in THF (9 mL) via a syringe pump over 75 min at 80 °C under an argon atmosphere, and the mixture was stirred at that temperature for 30 min. After removal of a solid

by filtration through a Celite pad, the filtrate was concentrated and purified by column chromatography on SiO_2 (1:30 ethyl acetate/hexane) to give **18** (3.59 g, 94%). **18**: ^{1}H NMR δ_H(400 MHz; $CDCl_3$; Me_4Si)/ppm 7.66 (2H, t, J = 1.2 Hz), 7.50 (2H, s), 7.48 (2H, t, J = 1.6 Hz), 7.48 (2H, dt, J = 1.2, 8.0 Hz), 7.45 (2H, dt, J = 1.2, 8.0 Hz), 7.32 (2H, t, J = 1.6 Hz), 7.29 (2H, br.), 7.27 (2H, t, J = 8.0 Hz), 3.53 (4H, t, J = 7.6 Hz), 1.49-1.42 (4H, m), 1.39 (18H, s), 1.29-1.20 (4H, m), 1.2-1.1 (42H, br.m), 0.85 (6H, t, J = 7.2 Hz), 0.23 (18H, s); ^{13}C NMR δ_C(100 MHz; $CDCl_3$)/ppm 154.1, 142.9, 134.9, 132.3, 132.2, 131.5, 131.2, 131.0, 130.0, 128.5, 127.7, 126.1, 124.1, 124.0, 123.6, 122.9, 105.9, 103.5, 96.9, 95.3, 94.8, 91.6, 88.2, 87.3, 80.4, 49.4, 30.5, 28.3, 19.8, 18.6, 13.7, 11.3, –0.1; FD-LRMS m/z 1372.7 (M^+, 84%), 1373.7 ($[M+1]^+$, 100), 1374.7 ($[M+2]^+$, 72), 1375.7 ($[M+3]^+$, 40), 1376.7 ($[M+4]^+$, 18), 1377.7 ($[M+5]^+$, 8).

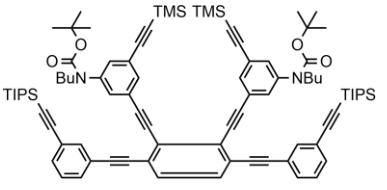

18

Preparation of **19**

To an ice-cooled solution of **18** (3.59 g, 2.61 mmol) in THF (52 mL) were added 60% NaH in oil (0.22 g, 5.5 mmol) and MeOH (1.6 mL), and the mixture was stirred at room temperature for 15 min, diluted with ethyl acetate, and then quenched with water in an ice water bath. The organic layer was separated, washed with brine, dried over magnesium sulfate, and then concentrated. The resulting solid was purified by column chromatography on SiO_2 (1:20 ethyl acetate/hexane) to give **18'** (3.11 g, 97%). **18'**: ^{1}H NMR δ_H(400 MHz; $CDCl_3$; Me_4Si)/ppm 7.68 (2H, t, J = 1.6 Hz), 7.55 (2H, t, J = 1.6 Hz), 7.53-7.51 (4H, m), 7.47 (2H, dt, J = 1.2, 8.0 Hz), 7.37 (2H, t, J = 1.6 Hz), 7.32 (2H, br.t), 7.30 (2H, t, J = 7.6 Hz), 3.55 (4H, t, J = 7.6 Hz), 3.08 (2H, s), 1.50-1.43 (4H, m), 1.40 (18H, s), 1.30-1.20 (4H, m), 1.2-1.1 (42H, br.m), 0.86 (6H, t, J = 7.2 Hz).

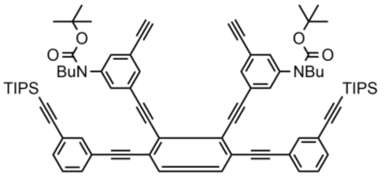

18'

To a solution of **23**[4] (886 mg, 1.67 mmol), $Pd(PPh_3)_4$ (145 mg, 0.126 mmol) and CuI (48 mg, 0.25 mmol) in Et_3N (38 mL) was added a solution of **18'** (257 mg, 0.209 mmol) in THF (4 mL) via a syringe pump over 6 h at 60 °C under an argon atmosphere, and the mixture was stirred at that temperature for 30 min. After removal of a solid by filtration through a Celite pad, the filtrate was concentrated and purified by column chromatography on SiO_2 (1:20 ethyl acetate/hexane) to give **19** (220 mg, 52%). **19**: ^{1}H NMR δ_H(400 MHz; $CDCl_3$; Me_4Si)/ppm 7.89 (2H, t, J = 1.6 Hz), 7.74-7.65 (6H, m), 7.62 (2H, t, J = 1.2 Hz), 7.60 (2H, t, J = 1.2 Hz), 7.53 (2H, dt, J = 1.2, 8.0 Hz), 7.52 (2H, s), 7.51-7.43 (10H, m), 7.40 (2H, dt, J = 1.6, 8.0 Hz), 7.37-7.26 (10H, m), 7.08 (2H, dd, J = 7.6, 8.0 Hz), 3.56 (4H, t, J = 7.6 Hz), 1.51-1.44 (4H, m), 1.40 (18H, s), 1.29-1.19 (4H, m), 1.12 (42H, s), 0.84 (6H, t, J = 7.2 Hz); ^{13}C NMR δ_C(100 MHz; $CDCl_3$)/ppm 154.1, 143.1, 140.2, 137.4, 135.0, 134.7, 132.3, 132.1, 131.6, 131.5, 131.5, 131.3, 130.7, 129.9, 128.6, 128.5, 128.4, 128.0, 125.9, 125.1, 124.1, 124.1, 123.9, 123.5, 123.3, 123.2, 123.2, 123.0, 105.9, 97.1, 94.8, 93.7, 91.8, 89.8, 89.4, 89.2, 89.1, 88.7, 88.3, 88.2, 87.7, 80.5, 49.5, 30.6, 28.3, 19.9, 18.7, 13.7, 11.3; FD-LRMS m/z 1016.3 (M^{2+}, 77%), 1016.9 ($[M+1]^{2+}$, 100), 1017.4 ($[M+2]^{2+}$, 90), 1017.8 ($[M+3]^{2+}$, 65), 1018.3 ($[M+4]^{2+}$, 36), 2032.7 (M^+, 59), 2033.7 ($[M+1]^+$, 95), 2034.7 ($[M+2]^+$, 78), 2035.7 ($[M+3]^+$, 44), 2036.7 ($[M+4]^+$, 19); FD-HRMS Found: 2032.67316, Calc. for $C_{126}H_{118}I_2N_2O_4Si_2$: 2032.67194.

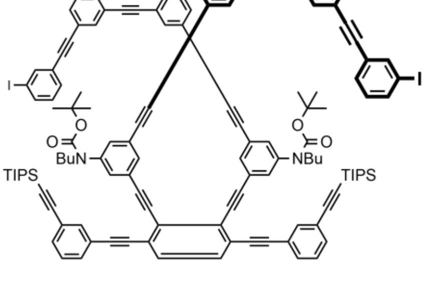

19

Preparation of 11PAMs (+)-**4**, (–)-**4** and **10**

To a solution of **19** (502 mg, 0.247 mmol) in THF (15 mL) was added TBAF (1 M in THF, 0.52 mL, 0.52 mmol) at room temperature, and the mixture was stirred at that temperature for 15 min. After dilution with dichloromethane and water, the organic layer was separated, washed with brine, dried over magnesium sulfate, and then concentrated. The resulting solid was purified by column chromatography on SiO_2 (1:4 ethyl acetate/hexane) to give **7** (330 mg, 78%). **7**: ^{1}H NMR δ_H(400 MHz; $CDCl_3$; Me_4Si)/ppm 7.89 (2H, t, J = 1.6 Hz), 7.72-7.66 (6H, m), 7.62 (2H, t, J = 1.6 Hz), 7.61 (2H, t, J = 1.2 Hz), 7.57 (2H, dt, J = 1.2, 7.6 Hz), 7.52 (2H, s), 7.51-7.45 (10H, m), 7.42 (2H, dt, J = 1.2, 8.0 Hz), 7.40-7.27 (10H, m), 7.08 (2H, t, J = 8.0 Hz), 3.58 (4H, t, J = 7.6 Hz), 3.07 (2H, s), 1.53-1.45 (4H, m), 1.41 (18H, s), 1.30-1.20 (4H, m), 0.84 (6H, t, J = 7.2 Hz).

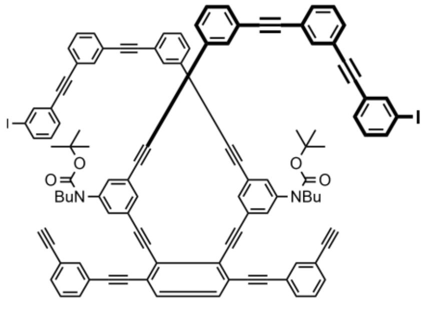

7

To a solution of $Pd(PPh_3)_4$ and CuI in Et_3N was added a solution of **7** in THF via a syringe pump over hours at 75 °C under an argon atmosphere [The reactions were implemented in two batches. The crude products were combined and purified at once]. After removal of the solvents by evaporation, the residue was dissolved in dichloromethane, which was washed with brine, dried over magnesium sulfate, and then purified by column chromatography on SiO_2 (1:2 ethyl acetate/hexane) to give *rac*-**4** (29 mg, 10%) and **10** (98 mg, 35%). Each product was further purified by GPC (chloroform; JAIGEL-2H & 2.5H, Japan Analytical Industry Co., Ltd., Japan), followed by HPLC separation with a standard normal-phase column (7:3 dichloromethane/hexane cont. 0.1vol% ethanol; YMC-Pack SIL, SIL-06, YMC Co., Ltd., Japan) to give a white solid, respectively. (+)-**4** and (–)-**4** were isolated in this order by HPLC separation with a chiral stationary column (3:7 chloroform/hexane; CHIRALPAK IA, DAICEL Co., Japan).

	**7** (total)	$Pd(PPh_3)_4$	CuI	Et_3N	time
1	330 mg (0.192 mmol) /58 mL THF	133 mg (0.115 mmol)	22 mg (0.12 mmol)	58 mL	19 h
2		133 mg (0.115 mmol)	22 mg (0.12 mmol)	58 mL	20 h

rac-**4**: IR (neat) ν_{max}/cm^{-1} 3062, 2959, 2929, 2871, 2210, 1701, 1595, 1583, 1482, 1367, 1146, 891, 789, 682; ^{1}H NMR δ_H(400 MHz; $CDCl_3$; Me_4Si)/ppm 7.90 (2H, br.s), 7.80-7.66 (6H, br.m), 7.60 (2H, br.s), 7.56-7.45 (14H, m), 7.52 (2H, s), 7.45-7.29 (10H, m), 7.26-7.21 (2H, br.m), 7.10 (2H, dd, J = 7.6, 8.0 Hz), 3.73-3.59 (2H, br.m), 3.50-3.33 (2H, br.m), 1.52-1.44 (4H, m), 1.26 (18H, s), 1.12-1.03 (4H, m), 0.65 (6H, t, J = 7.2 Hz); ^{13}C NMR δ_C(100 MHz; $CDCl_3$)/ppm 154.0, 143.9, 135.7, 135.3, 135.2, 135.1, 132.7, 131.8, 131.3, 131.2, 131.1, 131.1, 130.9, 130.6, 130.0 (br.), 129.1 (br.), 128.8 (br.), 128.7, 128.5, 128.5, 128.1, 125.4, 124.3, 124.0, 123.7, 123.7, 123.5, 123.4, 123.4, 123.3, 123.1, 122.9, 97.4 (br.), 95.0, 90.0 (br.), 89.6, 89.4, 89.3, 89.2, 89.1, 89.0, 88.6, 88.5, 87.6, 80.4, 49.9, 30.8, 28.1, 19.8, 13.6; FD-LRMS *m/z* 1464.6 (M^+, 81%), 1465.6 ($[M+1]^+$, 100), 1466.6 ($[M+2]^+$, 62), 1467.6 ($[M+3]^+$, 27), 1468.6 ($[M+4]^+$, 11); FD-HRMS Found: 1464.57911, Calc. for $C_{108}H_{76}N_2O_4$: 1464.58051; UV λ_{max}(CH_2Cl_2)/nm (log ε) 365 (shoulder 4.47), 340 (sh. 4.76), 320 (sh. 4.89), 304 (5.41), 287 (5.47), 272 (sh. 5.32), 258 (sh. 5.11).

(+)-**4** (1st): mp 185-188 °C (dec); $[\alpha]_D^{24}$ = +448 (c = 0.215 in chloroform); CD λ(CH_2Cl_2)/nm ($\Delta\varepsilon$) 359 (+51.7), 329

(+11.6), 307 (+194.8), 298 (+100.6), 293 (+86.6), 280 (–104.7), 261 (–16.6), 256 (–21.8).

(–)-**4** (2nd): $[\alpha]_D^{24} = -439$ (c = 0.228 in chloroform); CD $\lambda(CH_2Cl_2)$/nm ($\Delta\varepsilon$) 357 (–50.0), 329 (–9.5), 307 (–192.6), 298 (–96.5), 293 (–83.3), 280 (+108.2), 262 (+17.1), 257 (+22.6).

10: mp 196-199 °C (dec); IR (neat) ν_{max}/cm^{-1} 3058, 2958, 2929, 2871, 2204, 1701, 1594, 1581, 1481, 1366, 1145, 891, 789, 682; ^{1}H NMR δ_H(400 MHz; $CDCl_3$; Me_4Si)/ppm 7.83 (2H, br.t), 7.82-7.80 (4H, br.m), 7.72 (2H, br.t), 7.55-7.40 (18H, m), 7.53 (2H, s), 7.37-7.28 (12H, m), 3.49 (4H, t, J = 7.6 Hz), 1.49-1.41 (4H, m), 1.35 (18H, s), 1.29-1.20 (4H, m), 0.85 (6H, t, J = 7.2 Hz); ^{13}C NMR δ_C(100 MHz; $CDCl_3$)/ppm 154.1, 143.0, 138.3, 137.0, 136.8, 134.3, 132.4, 131.9, 131.4, 130.8, 130.6, 130.3, 130.2 (br.), 130.1, 130.1, 129.7, 129.6, 128.7, 128.6, 128.5, 128.1, 125.7, 123.7, 123.6, 123.5, 123.5, 123.4, 123.4, 123.4, 123.3, 96.6, 94.0, 90.0, 90.0, 90.0, 89.9, 89.2, 89.1, 89.0, 88.8, 87.7, 86.7, 80.4, 49.5, 30.5, 28.2, 19.9, 13.8; FD-LRMS m/z 1464.6 (M^+, 85%), 1465.6 ($[M+1]^+$, 100), 1466.6 ($[M+2]^+$, 65), 1467.6 ($[M+3]^+$, 30), 1468.6 ($[M+4]^+$, 9); FD-HRMS Found: 1464.57902, Calc. for $C_{108}H_{76}N_2O_4$: 1464.58051; UV $\lambda_{max}(CH_2Cl_2)$/nm (log ε) 304 (5.38), 287 (5.47), 279 (sh. 5.37), 272 (sh. 5.32), 256 (sh. 5.18).

Preparation of **21**

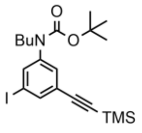

21

To a solution of **20**[1] (10.5 g, 24.8 mmol) in diethyl ether (250 mL) was added ^{n}BuLi (1.6 M in hexane, 16.6 mL, 26.1 mmol) at −78 °C under an argon atmosphere, and the mixture was stirred at that temperature for 1 min. To the mixture was added a solution of 1,2-diiodoethane (8.40 g, 29.8 mmol) in diethyl ether (85 mL), and the mixture was further stirred for 30 min. After addition of water, the organic layer was separated, dried over magnesium sulfate, and then roughly purified by column chromatography on SiO_2 (dichloromethane/hexane) to give an oil product (11.5 g, ca. 98%) containing **21**, which was subjected to the next reaction, even though the purification was incomplete. **21**: ^{1}H NMR δ_H(400 MHz; $CDCl_3$; Me_4Si)/ppm 7.63 (1H, t, J = 1.6 Hz), 7.50 (1H, t, J = 1.6 Hz), 7.24 (1H, br.t), 3.57 (2H, t, J = 7.6 Hz), 1.52-1.45 (2H, m), 1.43 (9H, s), 1.33-1.24 (2H, m), 0.90 (3H, t, J = 7.2 Hz), 0.24 (9H, s); ^{13}C NMR δ_C(100 MHz; $CDCl_3$)/ppm 154.0, 143.5, 137.8, 136.4, 129.5, 125.2, 102.6, 96.1, 92.6, 80.6, 49.5, 30.5, 28.3, 19.8, 13.7, –0.2; FD-LRMS m/z 471.1 (M^+, 100%), 472.1 ($[M+1]^+$, 29), 473.1 ($[M+2]^+$, 8).

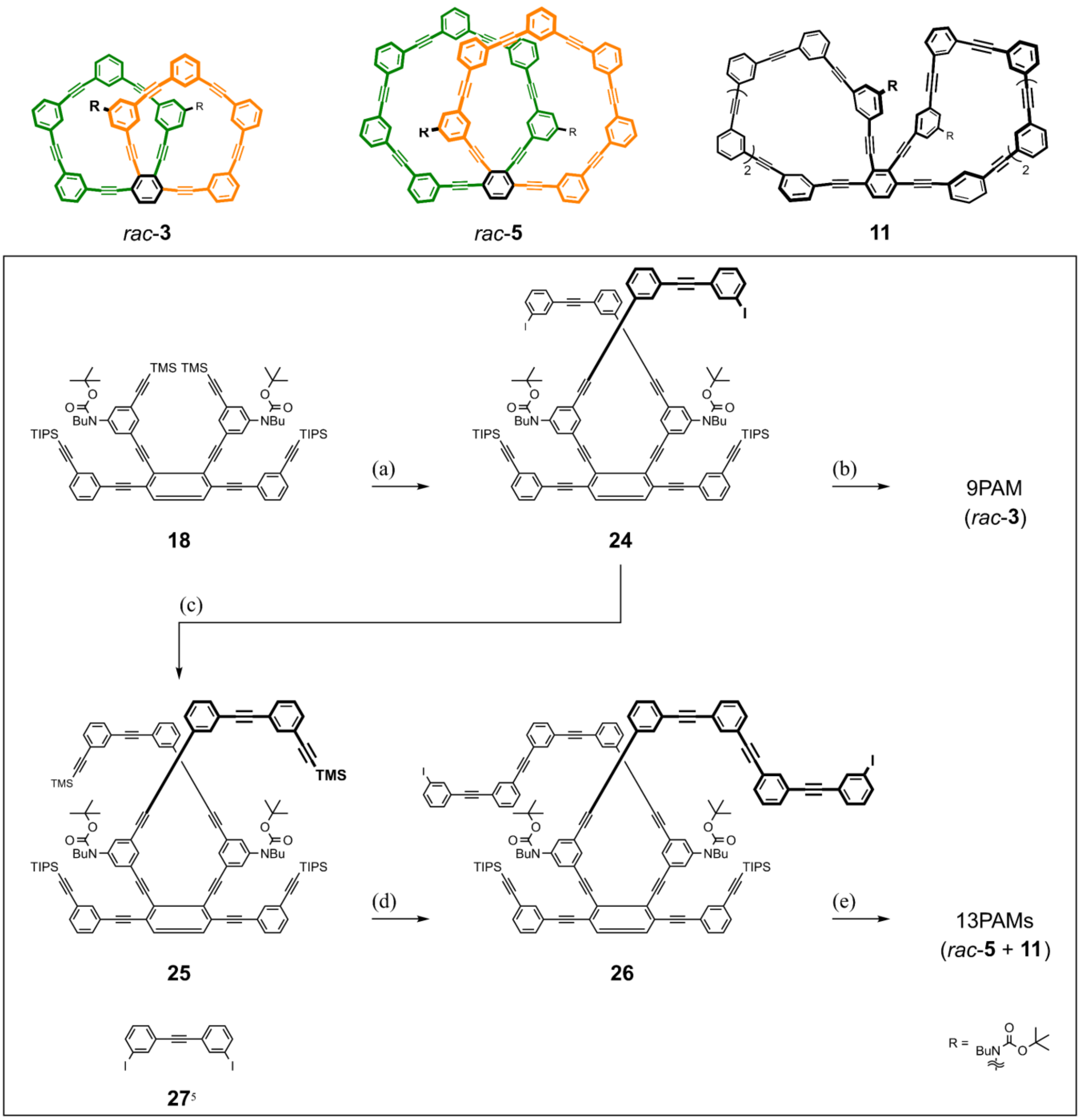

Scheme S2. Synthesis of *rac*-**3** (9PAM), *rac*-**5** (13PAM) and **11** (13PAM). Reagents and yields: (a) i) NaH, MeOH, THF, ii) **27**, $Pd(PPh_3)_4$, CuI, Et_3N, THF (71%); (b) i) TBAF, THF (85%), ii) $Pd(PPh_3)_4$, CuI, Et_3N, THF (42% for *rac*-**3**); (c) TMSA, $Pd(PPh_3)_4$, CuI, Et_3N (90%); (d) i) NaH, MeOH, THF (94%), ii) **27**, $Pd(PPh_3)_4$, CuI, Et_3N, THF (87%); (e) i) TBAF, THF (86%), ii) $Pd(PPh_3)_4$, CuI, Et_3N, THF (24% for *rac*-**5** and 12% for **11**).

Preparation of **24**

To a solution of **27**[5] (5.39 g, 12.5 mmol), $Pd(PPh_3)_4$ (1.09 g, 0.939 mmol) and CuI (358 mg, 1.88 mmol) in Et_3N (284 mL) was added a solution of **18'** (1.92 g, 1.56 mmol) in THF (28 mL) via a syringe pump over 1 h at 60 °C under an argon atmosphere, and the mixture was stirred at that temperature for 40 min. After removal of a solid by filtration through a Celite pad, the filtrate was concentrated and purified by column chromatography on SiO_2 (1:15 ethyl acetate/hexane) to give **24** (2.03 g, 71%). **24**: ^{1}H NMR δ_H(400 MHz; $CDCl_3$; Me_4Si)/ppm 7.88 (2H, t, J = 1.6 Hz), 7.69-7.66 (4H, m), 7.60-7.59 (4H, m), 7.53 (2H, dt, J = 1.6, 8.0 Hz), 7.52 (2H, s), 7.48 (2H, dt, J = 1.6, 8.0 Hz),

7.46-7.34 (10H, m), 7.31-7.26 (4H, m), 7.08 (2H, dd, J = 7.6, 8.0 Hz), 3.55 (4H, t, J = 7.6 Hz), 1.51-1.43 (4H, m), 1.40 (18H, s), 1.29-1.19 (4H, m), 1.12 (42H, s), 0.84 (6H, t, J = 7.2 Hz); ^{13}C NMR δ_C(100 MHz; $CDCl_3$)/ppm 154.1, 143.1, 140.2, 137.4, 135.0, 134.7, 132.3, 132.0, 131.7, 131.5, 131.4, 131.3, 130.7, 129.9, 128.5, 128.4, 128.0, 125.9, 125.1, 124.1, 124.1, 123.9, 123.3, 123.1, 123.0, 105.9, 97.1, 94.8, 93.7, 91.8, 89.8, 89.4, 88.7, 88.2, 88.2, 87.7, 80.5, 49.5, 30.6, 28.3, 19.9, 18.7, 13.7, 11.3; FD-LRMS m/z 1832.6 (M^+, 78%), 1833.6 ($[M+1]^+$, 100), 1834.6 ($[M+2]^+$, 75), 1835.6 ($[M+3]^+$, 44), 1836.6 ($[M+4]^+$, 17); FD-HRMS Found: 1832.60859, Calc. for $C_{110}H_{110}I_2N_2O_4Si_2$: 1832.60934.

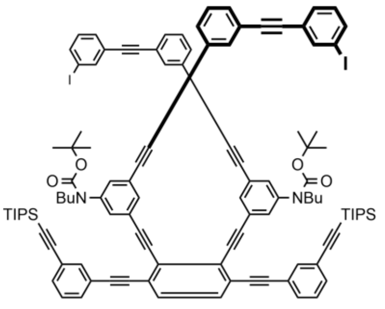

24

Preparation of 9PAM (*rac*-**3**)

To a solution of **24** (252 mg, 0.137 mmol) in THF (9 mL) was added TBAF (1 M in THF, 0.29 mL, 0.29 mmol) at room temperature, and the mixture was stirred at that temperature for 10 min. After dilution with ethyl acetate and water, the organic layer was separated, washed with brine, dried over magnesium sulfate, and then concentrated. The resulting solid was purified by column chromatography on SiO_2 (1:4 ethyl acetate/hexane) to give **6** (178 mg, 85%). **6**: ^{1}H NMR δ_H(400 MHz; $CDCl_3$; Me_4Si)/ppm 7.89 (2H, t, J = 1.6 Hz), 7.71 (2H. t, J = 1.6 Hz), 7.69-7.66 (2H, m), 7.61-7.59 (4H, m), 7.57 (2H, dt, J = 1.6, 7.6 Hz), 7.52 (2H, s), 7.49-7.36 (12H, m), 7.34-7.28 (4H, m), 7.08 (2H, t, J = 8.0 Hz), 3.58 (4H, t, J = 7.6 Hz), 3.07 (2H, s), 1.50-1.45 (4H, m), 1.40 (18H, s), 1.29-1.20 (4H, m), 0.84 (6H, t, J = 7.2 Hz).

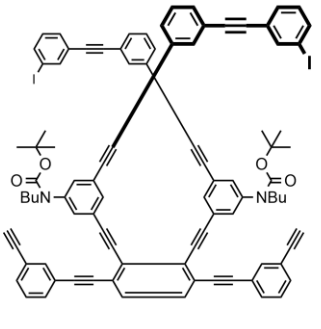

6

To a solution of $Pd(PPh_3)_4$ (81 mg, 0.070 mmol) and CuI (13 mg, 0.068 mmol) in Et_3N (35 mL) was added a solution of **6** (178 mg, 0.117 mmol) in THF (18 mL) via a syringe pump over 20 h at 75 °C under an argon atmosphere. After removal of the solvents by evaporation, the residue was dissolved in dichloromethane, which was washed with brine, dried over magnesium sulfate, and then purified by column chromatography on SiO_2 (1:4 ethyl acetate/hexane) to give *rac*-**3** (62 mg, 42%). An analytical sample was obtained as a white solid by further purification through GPC (chloroform), followed by HPLC separation with a standard normal-phase column (7:3 dichloromethane/hexane cont. 0.1vol% ethanol). Racemate of (+)-**3** and (–)-**3** was unfortunately inseparable by HPLC separation with either column (CHIRALPAK IA and IF).

rac-**3**: mp 179-183 °C (dec); IR (neat) ν_{max}/cm^{-1} 3061, 2958, 2930, 2871, 2204, 1701, 1593, 1577, 1482, 1367, 1145, 891, 789, 682; ^{1}H NMR δ_H(400 MHz; $CDCl_3$; Me_4Si)/ppm 8.06-8.01 (4H, m), 7.94 (2H, br.dd), 7.75 (2H, t, J = 1.6 Hz), 7.60 (2H, ddd, J = 1.2, 1.6, 8.0 Hz), 7.58 (2H, s), 7.53 (2H, ddd, J = 1.2, 1.6, 8.0 Hz), 7.46-7.33 (16H, m), 7.21 (2H, br.dd), 3.25 (4H, t, J = 7.6 Hz), 1.42 (18H, s), 1.41-1.34 (4H, m), 1.27-1.17 (4H, m), 0.88 (6H, t, J = 7.2 Hz); ^{13}C NMR δ_C(100 MHz; $CDCl_3$)/ppm 153.9, 143.4, 140.6, 140.3, 134.4, 133.6, 132.8, 131.4, 131.0, 130.3 (br.), 129.7, 129.5, 129.2, 128.8, 128.8, 128.5, 128.1, 128.1, 124.8, 123.8, 123.7, 123.7, 123.6, 123.5, 123.5, 123.4, 97.9, 95.1, 92.5, 92.4, 90.0, 89.9, 89.7, 89.6, 88.9, 88.2, 80.5, 49.5, 30.7, 28.3, 19.9, 13.8; FD-LRMS m/z 1264.5 (M^+, 95%), 1265.5 ($[M+1]^+$, 100), 1266.5 ($[M+2]^+$, 61), 1267.5 ($[M+3]^+$, 22), 1268.5 ($[M+4]^+$, 7); FD-HRMS Found: 1264.51662, Calc. for $C_{92}H_{68}N_2O_4$: 1264.51791; UV λ_{max}(CH_2Cl_2)/nm (log ε) 367 (sh. 4.38), 340 (sh. 4.77), 321 (sh. 4.95), 305

(5.33), 289 (5.34), 272 (sh. 5.15), 259 (sh. 4.99).

Preparation of **25**

To a solution of **24** (1.01 g, 0.549 mmol), $Pd(PPh_3)_4$ (25 mg, 0.022 mmol) and CuI (8 mg, 0.04 mmol) in Et_3N (11 mL) was added TMSA (0.77 mL, 5.4 mmol) at 40 °C under an argon atmosphere, and the mixture was stirred for 3 h. After removal of the solvent by evaporation, the residue was dissolved in dichloromethane, which was passed through a SiO_2/Celite pad. The filtrate was concentrated and purified by column chromatography on SiO_2 (1:15 ethyl acetate/hexane) to give **25** (880 mg, 90%). **25**: ^{1}H NMR δ_H(400 MHz; $CDCl_3$; Me_4Si)/ppm 7.69 (2H, t, J = 1.2 Hz), 7.64 (2H, t, J = 1.6 Hz), 7.61 (2H, t, J = 1.6 Hz), 7.59 (2H, t, J = 1.6 Hz), 7.53 (2H, dt, J = 1.6, 8.0 Hz), 7.52 (2H, s), 7.47-7.34 (14H, m), 7.31-7.26 (6H, m), 3.55 (4H, t, J = 7.6 Hz), 1.51-1.43 (4H, m), 1.40 (18H, s), 1.29-1.19 (4H, m), 1.12 (42H, s), 0.84 (6H, t, J = 7.2 Hz), 0.25 (18H, s); ^{13}C NMR δ_C(100 MHz; $CDCl_3$)/ppm 154.1, 143.1, 135.0, 135.0, 134.6, 132.3, 132.0, 131.7, 131.6, 131.5, 131.5, 131.3, 130.8, 129.9, 128.5, 128.4, 128.4, 128.0, 125.9, 124.1, 124.1, 123.9, 123.6, 123.3, 123.3, 123.2, 123.0, 105.9, 104.1, 97.1, 95.1, 94.8, 91.8, 89.4, 89.1, 89.0, 88.6, 88.2, 87.7, 80.5, 49.5, 30.5, 28.3, 19.8, 18.6, 13.7, 11.3, –0.1; FD-LRMS m/z 1772.9 (M^+, 63%), 1773.9 ($[M+1]^+$, 100), 1774.9 ($[M+2]^+$, 96), 1775.9 ($[M+3]^+$, 54), 1776.9 ($[M+4]^+$, 28), 1777.9 ($[M+5]^+$, 12); FD-HRMS Found: 1772.89664, Calc. for $C_{120}H_{128}N_2O_4Si_4$: 1772.89511.

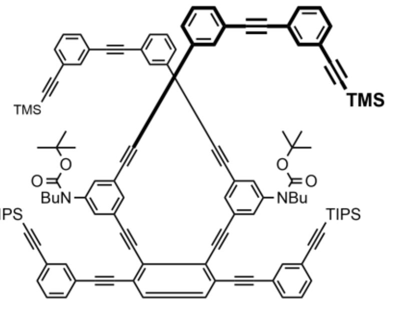

25

Preparation of **26**

To an ice-cooled solution of **25** (828 mg, 0.466 mmol) in THF (9 mL) were added 60% NaH in oil (39 mg, 0.98 mmol) and MeOH (0.28 mL), and the mixture was stirred at room temperature for 15 min, diluted with ethyl acetate, and then quenched with water in an ice water bath. The organic layer was separated, washed with brine, dried over magnesium sulfate, and then concentrated. The resulting solid was purified by column chromatography on SiO_2 (1:8 ethyl acetate/hexane) to give **25'** (711 mg, 94%). **25'**: ^{1}H NMR δ_H(400 MHz; $CDCl_3$; Me_4Si)/ppm 7.69 (2H, t, J = 1.6 Hz), 7.65 (2H, t, J = 1.6 Hz), 7.61 (2H, t, J = 1.6 Hz), 7.59 (2H, t, J = 1.6 Hz), 7.54-7.48 (4H, m), 7.52 (2H, s), 7.47-7.35 (12H, m), 7.33-7.26 (6H, m), 3.55 (4H, t, J = 7.6 Hz), 3.10 (2H, s), 1.51-1.43 (4H, m), 1.40 (18H, s), 1.29-1.21 (4H, m), 1.12 (42H, s), 0.84 (6H, t, J = 7.2 Hz).

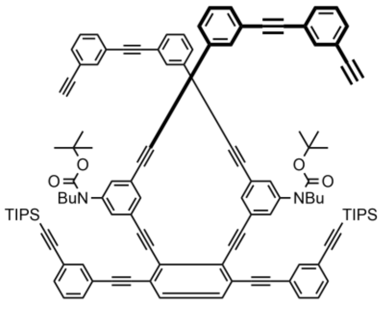

25'

To a solution of **27** (1.50 g, 3.50 mmol), $Pd(PPh_3)_4$ (303 mg, 0.262 mmol) and CuI (100 mg, 0.525 mmol) in Et_3N (79 mL) was added a solution of **25'** (711 mg, 0.436 mmol) in THF (8 mL) via a syringe pump over 1 h at 60 °C under an argon atmosphere, and the mixture was stirred at that temperature for 30 min. After removal of the solvents by evaporation, the residue was dissolved in dichloromethane, which was passed through a SiO_2/Celite pad. The filtrate was concentrated and purified by column chromatography on SiO_2 (1:8 ethyl acetate/hexane) to give **26** (849 mg, 87%).

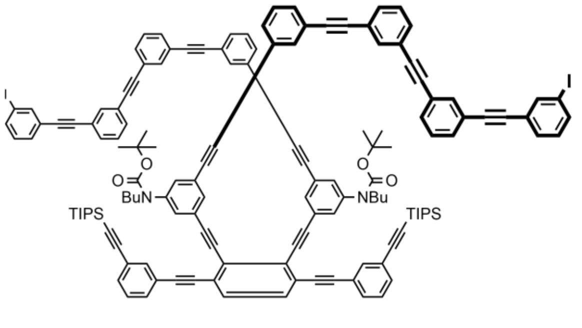

26

26: ^{1}H NMR δ_H(400 MHz; $CDCl_3$; Me_4Si)/ppm 7.89 (2H, t, J = 1.6 Hz), 7.71-7.66 (8H, m), 7.62 (2H, t, J = 1.2 Hz), 7.60 (2H, t, J = 1.2 Hz), 7.54-7.26 (32H, m), 7.08 (2H, dd, J = 7.6, 8.0 Hz), 3.56 (4H, t, J = 7.6 Hz), 1.51-1.44 (4H, m), 1.40 (18H, s), 1.29-1.19 (4H, m), 1.12 (42H, s), 0.84 (6H, t, J = 7.2 Hz); ^{13}C NMR δ_C(100 MHz; $CDCl_3$)/ppm 154.2, 143.2, 140.2, 137.4, 135.0, 134.7, 134.7, 132.3, 132.1, 131.6, 131.5, 131.5, 131.5, 131.3, 130.7, 129.9, 128.6, 128.5, 128.4, 128.0, 125.9, 125.1, 124.1, 124.1, 123.9, 123.5, 123.4, 123.3, 123.2, 123.2, 123.0, 105.9, 97.1, 94.8, 93.7, 91.8, 89.8, 89.5, 89.2, 89.2, 89.1, 89.1, 88.7, 88.3, 88.2, 87.7, 80.5, 49.5, 30.6, 28.3, 19.9, 18.7, 13.7, 11.3; FD-LRMS *m/z* 1116.4 (M^{2+}, 57%), 1116.9 ($[M+1]^{2+}$, 100), 1117.4 ($[M+2]^{2+}$, 99), 1117.9 ($[M+3]^{2+}$, 74), 1118.4 ($[M+4]^{2+}$, 47), 1118.9 ($[M+5]^{2+}$, 30), 2232.8 (M^+, 35), 2233.8 ($[M+1]^+$, 67), 2234.8 ($[M+2]^+$, 63), 2235.8 ($[M+3]^+$, 40), 2236.8 ($[M+4]^+$, 20), 2237.8 ($[M+5]^+$, 9); FD-HRMS Found: 2232.73474, Calc. for $C_{142}H_{126}I_2N_2O_4Si_2$: 2232.73454.

Preparation of 13PAMs (+)-**5**, (–)-**5** and **11**

To a solution of **26** (1.43 g, 0.638 mmol) in THF (40 mL) was added TBAF (1 M in THF, 1.34 mL, 1.34 mmol) at room temperature, and the mixture was stirred at that temperature for 17 min. After dilution with dichloromethane and water, the organic layer was separated, washed with brine, dried over magnesium sulfate, and then concentrated. The resulting solid was purified by column chromatography on SiO_2 (1:6 ethyl acetate/hexane) to give **8** (1.06 g, 86%). **8**: ^{1}H NMR δ_H(400 MHz; $CDCl_3$; Me_4Si)/ppm 7.89 (2H, t, J = 1.6 Hz), 7.71-7.66 (8H, m), 7.63 (2H, t, J = 1.2 Hz), 7.62 (2H, t, J = 1.2 Hz), 7.65 (2H, dt, J = 1.6, 7.6 Hz), 7.51-7.28 (30H, m), 7.08 (2H, t, J = 8.0 Hz), 3.58 (4H, t, J = 7.6 Hz), 3.08 (2H, s), 1.53-1.45 (4H, m), 1.41 (18H, s), 1.30-1.20 (4H, m), 0.84 (6H, t, J = 7.2 Hz).

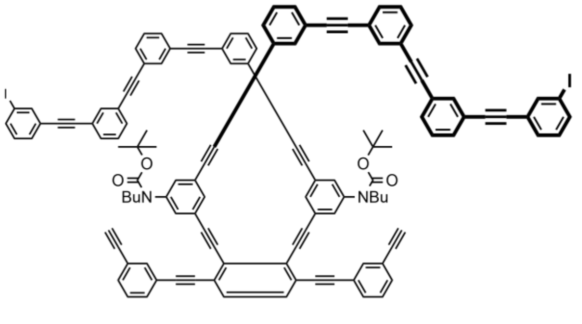

8

To a solution of $Pd(PPh_3)_4$ and CuI in Et_3N was added a solution of **8** in THF via a syringe pump over hours at 75 °C under an argon atmosphere [The reactions were implemented in five batches. The crude products were combined and purified at once]. After removal of the solvents by evaporation, the residue was dissolved in dichloromethane, which was washed with brine, dried over magnesium sulfate, and then purified by column chromatography on SiO_2 (1:2 ethyl acetate/hexane) to give *rac*-**5** (217 mg, 24%) and **11** (109 mg, 12%). Each product was further purified by GPC (chloroform), followed by HPLC separation with a standard normal-phase column (6:4 dichloromethane/hexane cont. 0.1vol% ethanol) to give a white solid, respectively. (+)-**5** and (–)-**5** were isolated in

this order by HPLC separation with a chiral stationary column (3:7 chloroform/hexane; CHIRALPAK IA).

	**8** (total)	$Pd(PPh_3)_4$	CuI	Et_3N	time
1	1.02 g (0.533 mmol) /160 mL THF	148 mg (0.128 mmol)	25 mg (0.13 mmol)	64 mL	21 h
2		148 mg (0.128 mmol)	24 mg (0.13 mmol)	64 mL	22 h
3		148 mg (0.128 mmol)	24 mg (0.13 mmol)	64 mL	22 h
4		147 mg (0.127 mmol)	25 mg (0.13 mmol)	64 mL	21 h
5		147 mg (0.127 mmol)	26 mg (0.14 mmol)	64 mL	21 h

rac-**5**: mp 164-167 °C (dec); IR (neat) ν_{max}/cm^{-1} 3061, 2960, 2929, 2871, 2210, 1700, 1597, 1583, 1482, 1367, 1145, 890, 789, 682; ^{1}H NMR δ_H(400 MHz; $CDCl_3$; Me_4Si)/ppm 7.74 (2H, br.t), 7.71-7.69 (4H, br.m), 7.66 (2H, br.t), 7.64 (2H, br.t), 7.59-7.49 (18H, m), 7.46 (2H, dt, J = 1.2, 8.0 Hz), 7.42-7.30 (16H, m), 7.09 (2H, dd, J = 7.6, 8.0 Hz), 3.63-3.52 (4H, m), 1.50-1.43 (4H, m), 1.36 (18H, s), 1.25-1.18 (4H, m), 0.80 (6H, t, J = 7.2 Hz); ^{13}C NMR δ_C(100 MHz; $CDCl_3$)/ppm 154.1, 143.1, 134.4, 134.4, 134.2, 134.1, 132.2, 132.0, 131.9, 131.8, 131.8, 131.7, 131.7, 131.6, 131.6, 131.5, 130.9, 130.0, 128.6, 128.5, 128.4, 128.4, 128.4, 128.2, 125.5, 124.2, 123.8, 123.7, 123.5 (sh.), 123.5, 123.4, 123.4, 123.3, 123.2, 123.1, 97.4, 94.5, 89.6, 89.3, 89.3, 89.2, 89.1, 89.1, 89.0, 88.9, 88.6, 88.2, 87.5, 80.4, 49.3, 30.5, 28.2, 19.9, 13.7; FD-LRMS m/z 1664.7 (M^+, 75%), 1665.7 ($[M+1]^+$, 100), 1666.7 ($[M+2]^+$, 69), 1667.7 ($[M+3]^+$, 33), 1668.7 ($[M+4]^+$, 12); FD-HRMS Found: 1664.64403, Calc. for $C_{124}H_{84}N_2O_4$: 1664.64311; UV λ_{max}(CH_2Cl_2)/nm (log ε) 365 (sh. 4.26), 340 (sh. 4.73), 323 (sh. 4.89), 304 (5.42), 287 (5.50), 272 (sh. 5.37), 257 (sh. 5.20).

(+)-**5** (1st): $[\alpha]_D^{24}$ = +391 (c = 0.188 in chloroform); CD λ(CH_2Cl_2)/nm ($\Delta\varepsilon$) 358 (+27.1), 346 (+17.6), 341 (+19.5), 326 (+11.2), 307 (+239.7), 298 (sh. +114.5), 293 (sh. +88.0), 279 (–92.3), 271 (sh. –73.8).

(–)-**5** (2nd): $[\alpha]_D^{24}$ = −373 (c = 0.154 in chloroform); CD λ(CH_2Cl_2)/nm ($\Delta\varepsilon$) 357 (–26.0), 346 (–15.8), 340 (–18.2), 327 (–10.3), 307 (–233.5), 298 (sh. –108.3), 293 (sh. –84.2), 279 (+94.1), 271 (sh. +75.6).

11: mp 179-184 °C (dec); IR (neat) ν_{max}/cm^{-1} 3062, 2958, 2929, 2871, 2207, 1701, 1595, 1581, 1481, 1366, 1144, 890, 789, 682; ^{1}H NMR δ_H(400 MHz; $CDCl_3$; Me_4Si)/ppm 7.80 (2H, br.t), 7.78 (2H, br.t), 7.71-7.66 (6H, br.m), 7.57-7.41 (22H, m), 7.52 (2H, s), 7.39-7.28 (14H, m), 3.51 (4H, t, J = 7.6 Hz), 1.50-1.43 (4H, m), 1.37 (18H, s), 1.28-1.19 (4H, m), 0.84 (6H, t, J = 7.2 Hz); ^{13}C NMR δ_C(100 MHz; $CDCl_3$)/ppm 154.1, 143.1, 136.2, 136.1, 135.4, 135.3, 133.9, 132.3, 131.9, 131.4, 131.4, 131.3, 131.2, 131.2, 131.1, 131.0, 130.8, 130.7, 130.7, 130.1 (br.), 128.6, 128.6, 128.5, 128.1, 126.0, 123.9, 123.8, 123.6, 123.4 (sh.), 123.4, 123.4, 123.3, 123.2, 96.9, 94.5, 89.5, 89.4, 89.4, 89.4, 89.2, 89.1, 89.0, 88.6, 88.0, 87.2, 80.4, 49.4, 30.5, 28.3, 19.9, 13.7; FD-LRMS m/z 1664.6 (M^+, 67%), 1665.7 ($[M+1]^+$, 100), 1666.7 ($[M+2]^+$, 67), 1667.7 ($[M+3]^+$, 37), 1668.7 ($[M+4]^+$, 11); FD-HRMS Found: 1664.64185, Calc. for $C_{124}H_{84}N_2O_4$: 1664.64311; UV λ_{max}(CH_2Cl_2)/nm (log ε) 304 (5.47), 287 (5.53), 279 (sh. 5.43), 272 (sh. 5.38), 256 (sh. 5.22).

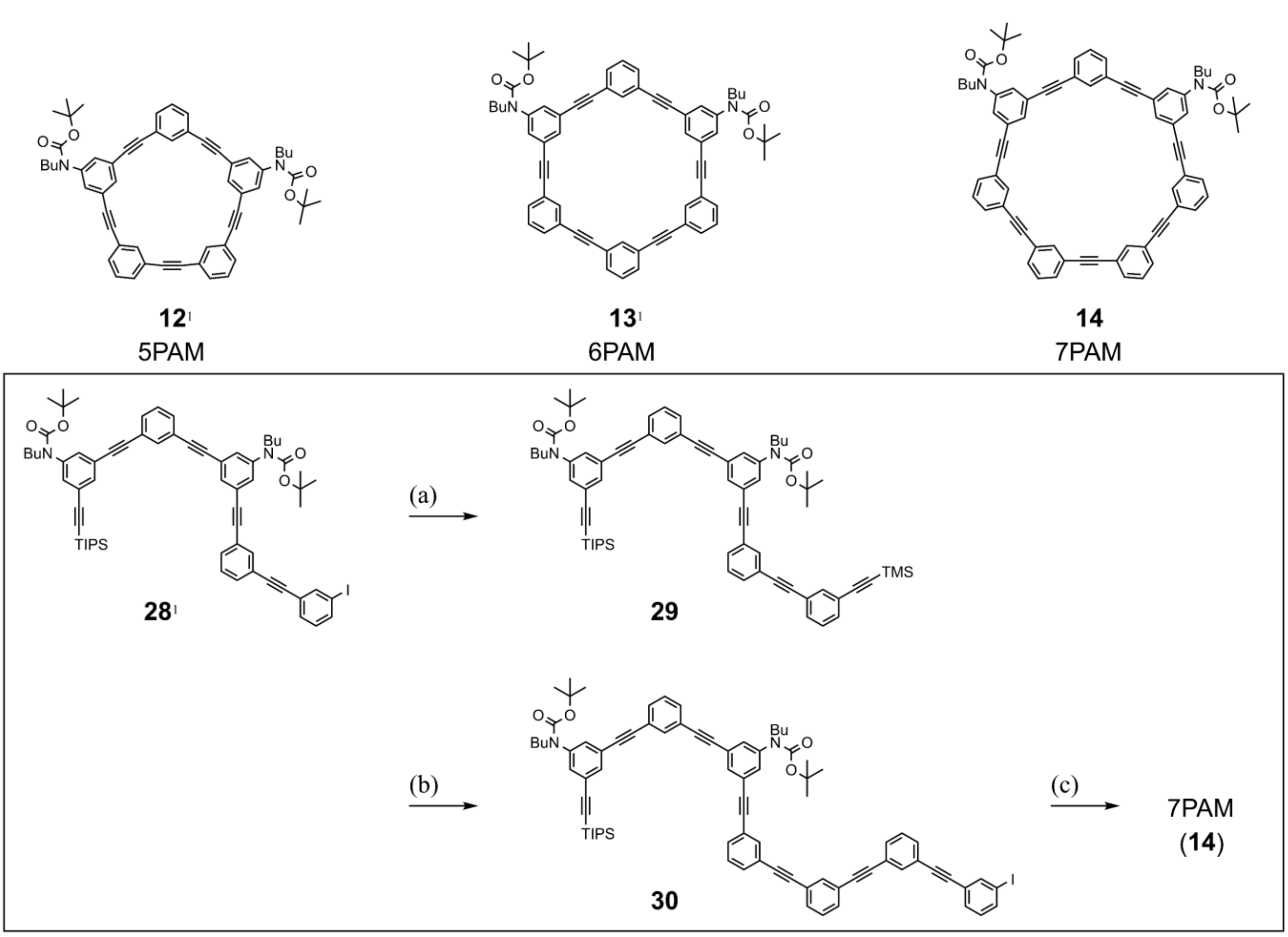

Scheme S3. Synthesis of reference ring **14** (7PAM). Reagents and yields: (a) TMSA, $Pd(PPh_3)_4$, CuI, Et_3N, THF (87%); (b) i) K_2CO_3, MeOH, THF (91%), ii) **27**, $Pd(PPh_3)_4$, CuI, Et_3N, THF (89%); (c) i) TBAF, THF (86%), ii) $Pd(PPh_3)_4$, CuI, Et_3N, THF (75%).

Preparation of **29**

To a solution of **28**[1] (998 mg, 0.886 mmol), $Pd(PPh_3)_4$ (20 mg, 0.017 mmol) and CuI (7 mg, 0.04 mmol) in Et_3N (3 mL) and THF (3 mL) was added TMSA (0.25 mL, 1.8 mmol) at room temperature under an argon atmosphere, and the mixture was stirred for 4 h. After removal of a solid by filtration through a Celite pad, the filtrate was concentrated and purified by column chromatography on SiO_2 (1:1-2:1-3:1 dichloromethane/hexane) to give **29** (845 mg, 87%). **29**: ^{1}H NMR δ_H(400 MHz; $CDCl_3$; Me_4Si)/ppm 7.71 (1H, br.t), 7.70 (1H, br.t), 7.66 (1H, br.t), 7.54 (1H, t, *J* = 1.2 Hz), 7.52-7.45 (6H, m), 7.43 (1H, ddd, *J* = 1.2, 1.6, 8.0 Hz), 7.37-7.26 (7H, m), 3.65 (2H, t, *J* = 7.2 Hz), 3.63 (2H, t, *J* = 7.6 Hz), 1.63-1.47 (4H, m), 1.47 (9H, s), 1.45 (9H, s), 1.39-1.28 (4H, m), 1.14 (21H, br.s), 0.93 (3H, t, *J* = 7.6 Hz), 0.92 (3H, t, *J* = 7.2 Hz), 0.26 (9H, s); ^{13}C NMR δ_C(100 MHz; $CDCl_3$)/ppm 154.3, 154.3, 143.0, 142.8, 135.1, 134.7, 134.7, 132.6, 132.1, 131.8, 131.6, 131.5, 130.5, 130.3, 130.2, 128.6, 128.4, 124.4, 123.9, 123.9, 123.6, 123.6, 123.5, 123.3, 123.3, 123.3, 123.2, 105.6, 104.0, 95.1, 91.9, 89.3, 89.3, 89.2, 89.1, 89.0, 88.8, 88.8, 88.7, 80.5, 80.5, 49.6, 46.3, 30.6, 30.6, 28.3, 28.3, 19.9, 19.9, 18.6, 13.8, 13.8, 11.3, –0.1; FD-LRMS *m/z* 1096.6 (M^+, 100%), 1097.6 ($[M+1]^+$, 93), 1098.6 ($[M+2]^+$, 50), 1099.6 ($[M+3]^+$, 19); FD-HRMS Found: 1096.59733, Calc. for $C_{72}H_{84}N_2O_4Si_2$: 1096.59696.

Preparation of **30**

To a solution of **29** (2.23 g, 2.03 mmol) in THF (11 mL) and MeOH (11 mL) was added K_2CO_3 (287 mg, 2.11 mmol) at room temperature, and the mixture was stirred at that temperature for 35 min. After removal of a solid by filtration through a Celite pad, the filtrate was concentrated and purified by column chromatography on SiO_2 (1:4 dichloromethane/hexane-dichloromethane) to give **29'** (1.90 g, 91%). **29'**: ^{1}H NMR δ_H(400 MHz; $CDCl_3$; Me_4Si)/ppm 7.72-7.71 (2H, br.m), 7.67 (1H, t, J = 1.6 Hz), 7.54 (1H, t, J = 1.2 Hz), 7.53-7.46 (7H, br.m), 7.38-7.26 (7H, m), 3.65 (2H, t, J = 7.6 Hz), 3.63 (2H, t, J = 7.6 Hz), 3.10 (1H, s), 1.59-1.50 (4H, m), 1.47 (9H, s), 1.45 (9H, s), 1.37-1.29 (4H, m), 1.14 (21H, br.s), 0.93 (3H, t, J = 7.6 Hz), 0.92 (3H, t, J = 7.2 Hz).

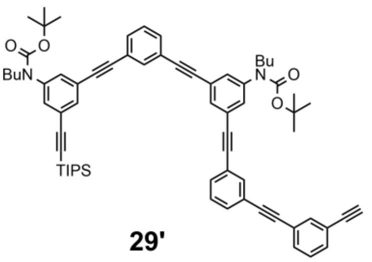

29'

To a solution of **27** (3.04 g, 7.08 mmol), $Pd(PPh_3)_4$ (601 mg, 0.520 mmol) and CuI (198 mg, 1.04 mmol) in Et_3N (173 mL) was added a solution of **29'** (1.90 g, 1.73 mmol) in THF (17 mL) via a syringe pump over 3 h at 60 °C under an argon atmosphere, and the mixture was stirred at that temperature for 30 min. After removal of a solid by filtration through a Celite pad, the filtrate was concentrated and purified by column chromatography on SiO_2 (1:1 dichloromethane/hexane-dichloromethane) to give **30** (2.05 g, 89%). **30**: ^{1}H NMR δ_H(400 MHz; $CDCl_3$; Me_4Si)/ppm 7.90 (1H, t, J = 1.6 Hz), 7.74-7.69 (4H, m), 7.68 (1H, ddd, J = 1.2, 1.6, 8.0 Hz), 7.55 (1H, t, J = 1.6 Hz), 7.53-7.47 (10H, m), 7.39-7.26 (8H, m), 7.09 (1H, t, J = 8.0 Hz), 3.65 (2H, t, J = 7.6 Hz), 3.62 (2H, t, J = 7.6 Hz), 1.59-1.47 (4H, m), 1.47 (9H, s), 1.45 (9H, s), 1.38-1.27 (4H, m), 1.14 (21H, br.s), 0.93 (3H, t, J = 7.2 Hz), 0.92 (3H, t, J = 7.2 Hz); ^{13}C NMR δ_C(100 MHz; $CDCl_3$)/ppm 154.3, 154.3, 143.0, 142.8, 140.2, 137.4, 134.7, 134.7, 132.6, 132.1, 131.6, 131.6, 131.5, 131.5, 130.7, 130.5, 130.3, 130.2, 129.8, 128.6, 125.1, 124.4, 123.9, 123.9, 123.6, 123.4, 123.4, 123.3, 123.3, 123.2, 105.6, 93.7, 91.9, 89.8, 89.3, 89.3, 89.2, 89.1, 89.1, 89.1, 88.8, 88.8, 88.7, 88.3, 80.5, 80.5, 49.6, 30.6, 30.6, 28.3, 28.3, 19.9, 19.9, 18.6, 13.8, 13.8, 11.3; FD-LRMS m/z 1326.5 (M^+, 100%), 1327.5 ($[M+1]^+$, 97), 1328.5 ($[M+2]^+$, 52), 1329.6 ($[M+3]^+$, 21); FD-HRMS Found: 1326.51652, Calc. for $C_{83}H_{83}IN_2O_4Si$: 1326.51668.

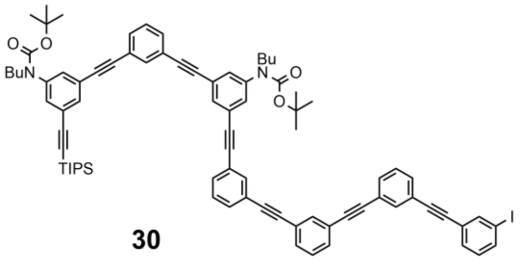

30

Preparation of **14** (7PAM)

To a solution of **30** (416 mg, 0.313 mmol) in THF (9 mL) was added TBAF (1 M in THF, 0.33 mL, 0.33 mmol) at room temperature, and the mixture was stirred at that temperature for 20 min. The reaction mixture was diluted with ethyl acetate, which was neutralized by a little amount of 0.1M aq. HCl and washed with water. The organic layer was separated, concentrated and then purified by column chromatography on SiO_2 (1:1 dichloromethane/hexane-dichloromethane) to give **30'** (316 mg, 86%). **30'**: ^{1}H NMR δ_H(400 MHz; $CDCl_3$; Me_4Si)/ppm 7.90 (1H, t, J = 1.6 Hz), 7.73-7.67 (4H, m), 7.56-7.48 (12H, m), 7.39-7.26 (8H, m), 7.09 (1H, t, J = 8.0 Hz), 3.65 (2H, t, J = 7.6 Hz), 3.62 (2H, t, J = 7.6 Hz), 3.11 (1H, s), 1.59-1.48 (4H, m), 1.47 (9H, s), 1.45 (9H, s), 1.37-1.25 (4H, m), 0.93 (3H, t, J = 7.2 Hz), 0.91 (3H, t, J = 7.2 Hz).

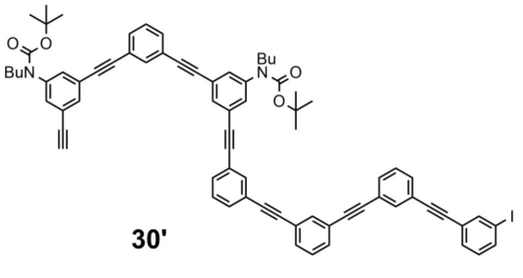

30'

To a solution of $Pd(PPh_3)_4$ (139 mg, 0.120 mmol) and CuI (23 mg, 0.12 mmol) in Et_3N (30 mL) was added a solution of **30'** (234 mg, 0.200 mmol) in THF (15 mL) via a syringe pump over 18 h at 70-80 °C under an argon atmosphere. After removal of a solid by filtration through a Celite pad, the filtrate was diluted with dichloromethane, which was washed with water, dried over magnesium sulfate, and then concentrated. The resulting solid was purified by column chromatography on SiO_2 (dichloromethane) to give **14** (156 mg, 75%, low reproducibility). An analytical sample was obtained as a white amorphous solid by further purification through GPC (chloroform), followed by HPLC separation with a standard normal-phase column (9:1 dichloromethane/hexane cont. 0.1vol% ethanol). **14**: ^{1}H NMR δ_H(400 MHz; $CDCl_3$; Me_4Si)/ppm 7.78-7.66 (5H, br.), 7.56 (2H, t, J = 1.2 Hz), 7.55-7.49 (10H, m), 7.42-7.33 (9H, m), 3.65 (4H, t, J = 7.6 Hz), 1.59-1.52 (4H, m), 1.47 (18H, s), 1.38-1.29 (4H, m), 0.93 (6H, t, J = 7.2 Hz); ^{13}C NMR δ_C(100 MHz; $CDCl_3$)/ppm 154.3, 143.0, 134.3, 134.3, 134.2, 131.9, 131.8, 131.7, 130.5, 128.5, 123.9, 123.9, 123.5, 123.4, 123.4, 123.3, 123.3, 89.3, 89.2, 89.2, 89.1, 89.1, 88.8, 88.7, 80.6, 49.6, 30.6, 28.4, 19.9, 13.8; FD-LRMS *m*/*z* 1042.5 (M^+, 100%), 1043.5 ($[M+1]^+$, 88), 1044.5 ($[M+2]^+$, 40), 1045.5 ($[M+3]^+$, 14); FD-HRMS Found: 1042.47229, Calc. for $C_{74}H_{62}N_2O_4$: 1042.47096.

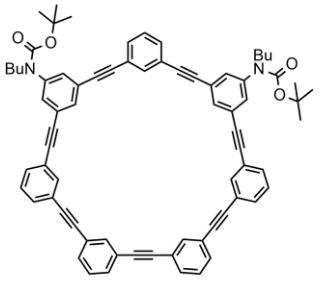

14

References

1 R. Katoono, Y. Obara, K. Kusaka and T. Suzuki, *Chem. Commun.*, 2018, **54**, 735.

2 V. Diemer, F. R. Leroux and F. Colobert, *Eur. J. Org. Chem.*, 2011, 327.

3 S. Y.-L. Leung, A. Y.-Y. Tam, C.-H. Tao, H. S. Chow and V. W.-W. Yam, *J. Am. Chem. Soc.*, 2012, **134**, 1047.

4 H.-B. Yang, K. Ghosh, N. Das and P. J. Stang, *Org. Lett.*, 2006, **8**, 3991.

5 A. Orita, K. Miyamoto, M. Nakashima, F. Ye and J. Otera, *Adv. Synth. Catal.*, 2004, **346**, 767.

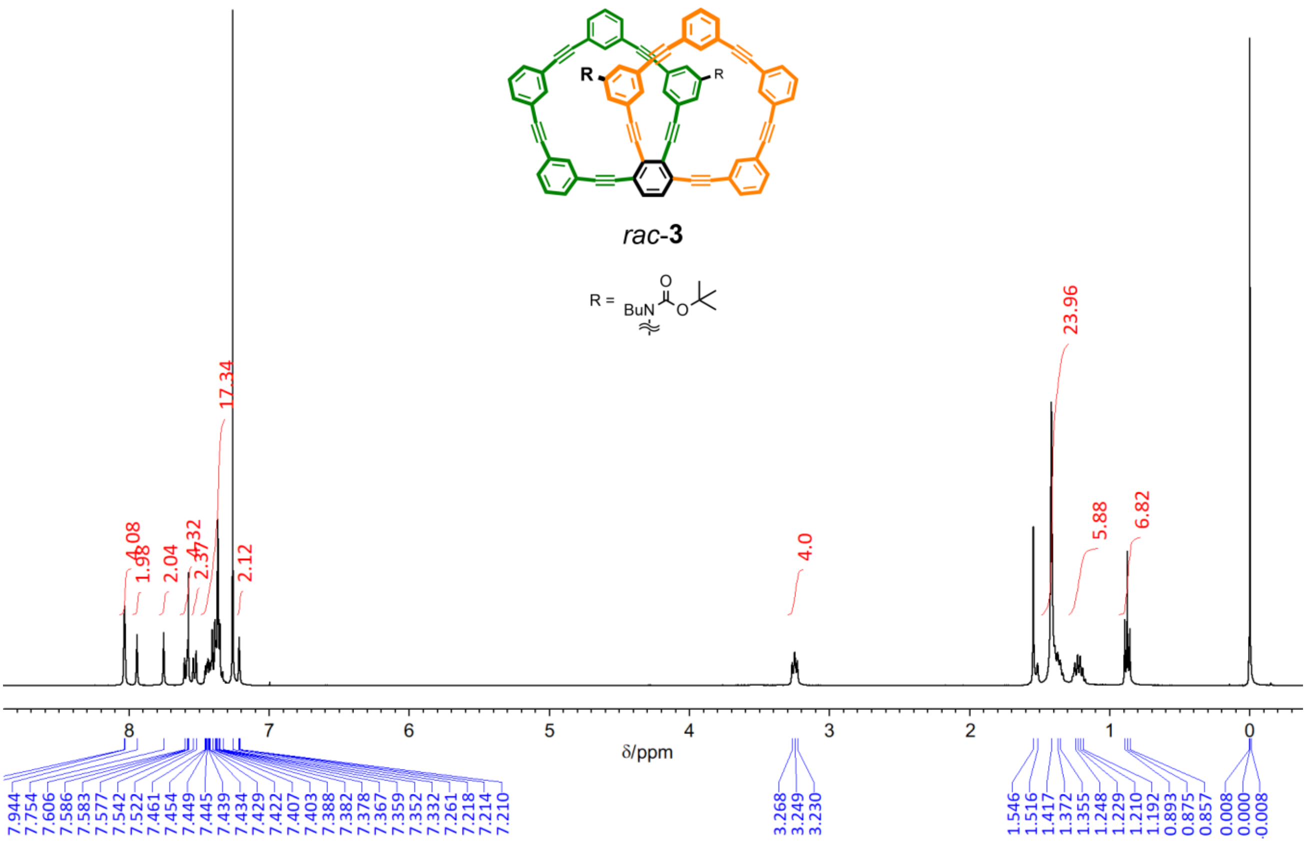

^{1}H NMR spectrum (400 MHz) of *rac*-**3** (9PAM), measured in chloroform-*d* at room temperature.

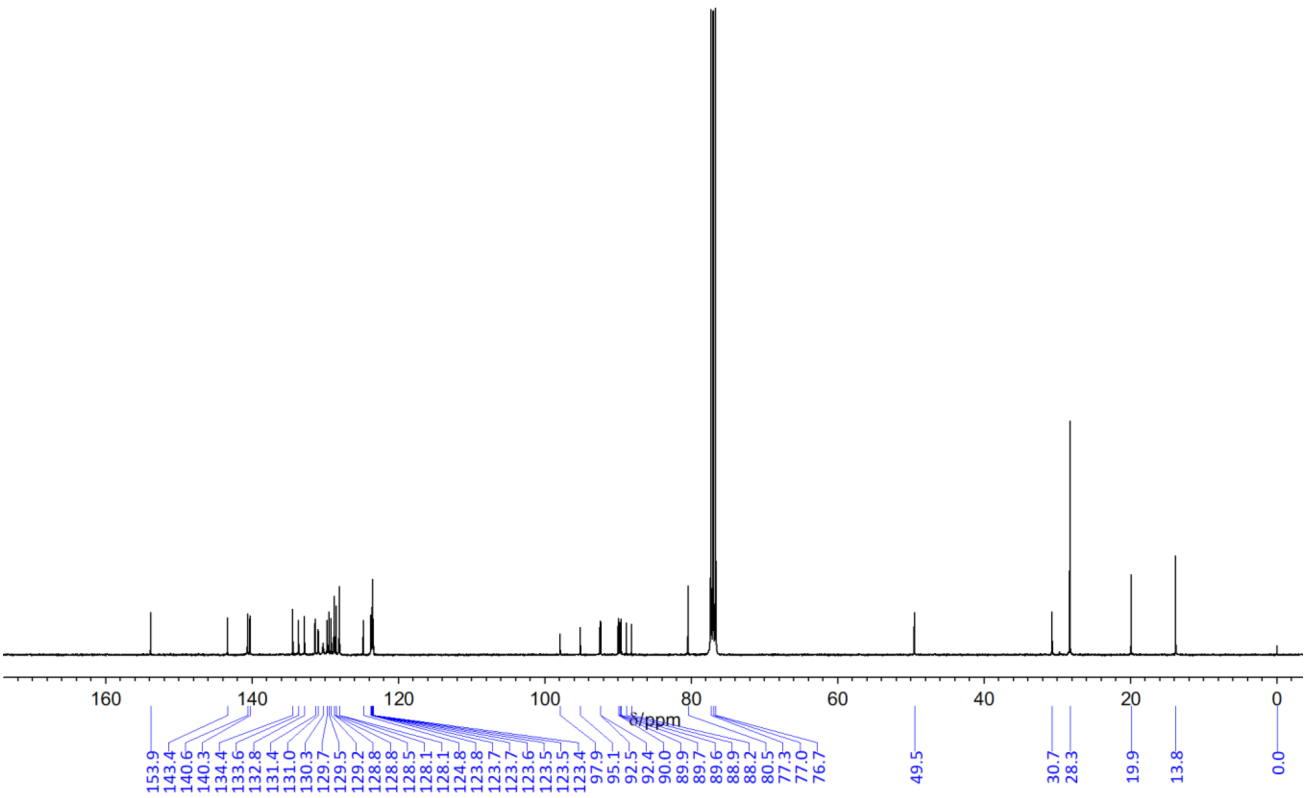

^{13}C NMR spectrum (100 MHz) of *rac*-**3** (9PAM), measured in chloroform-*d* at room temperature.

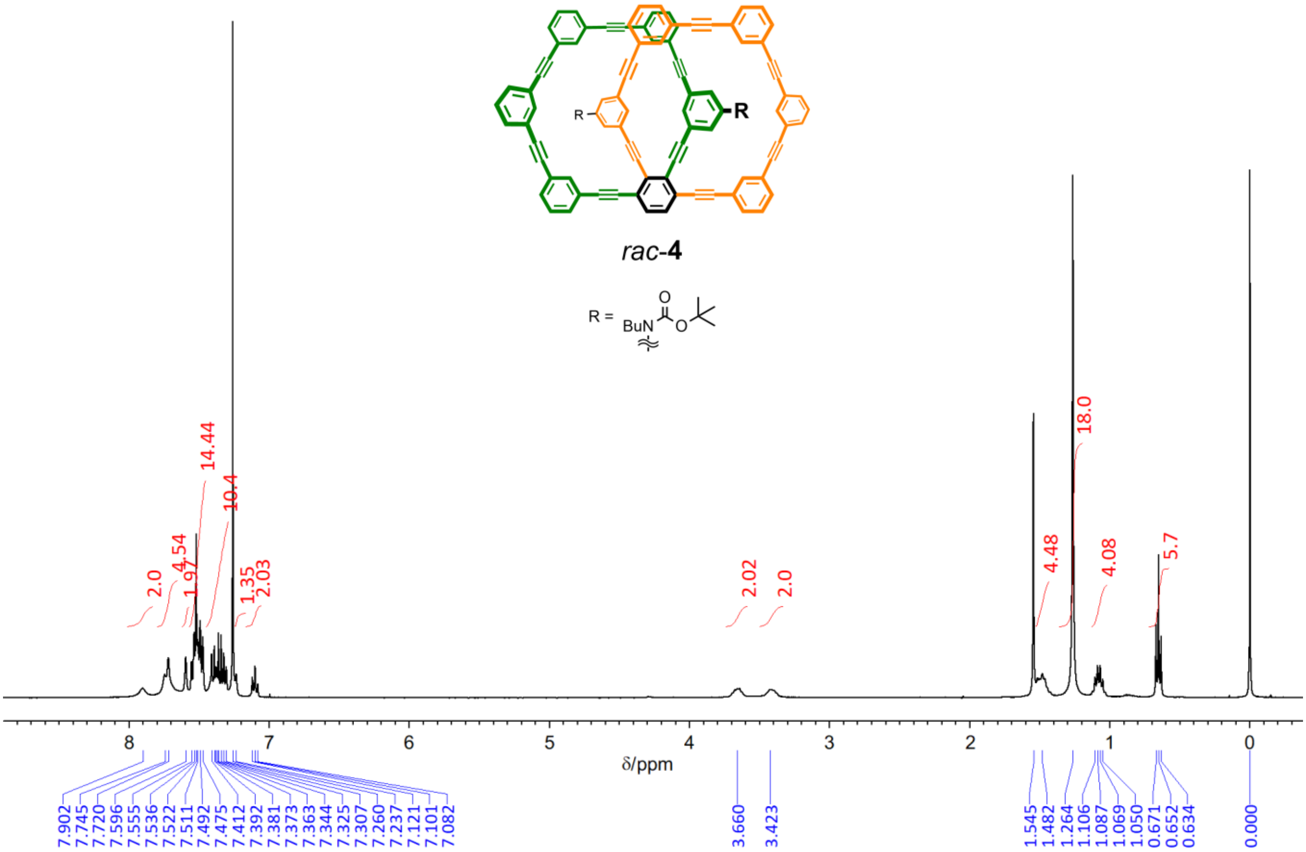

^{1}H NMR spectrum (400 MHz) of *rac*-**4** (11PAM), measured in chloroform-*d* at room temperature.

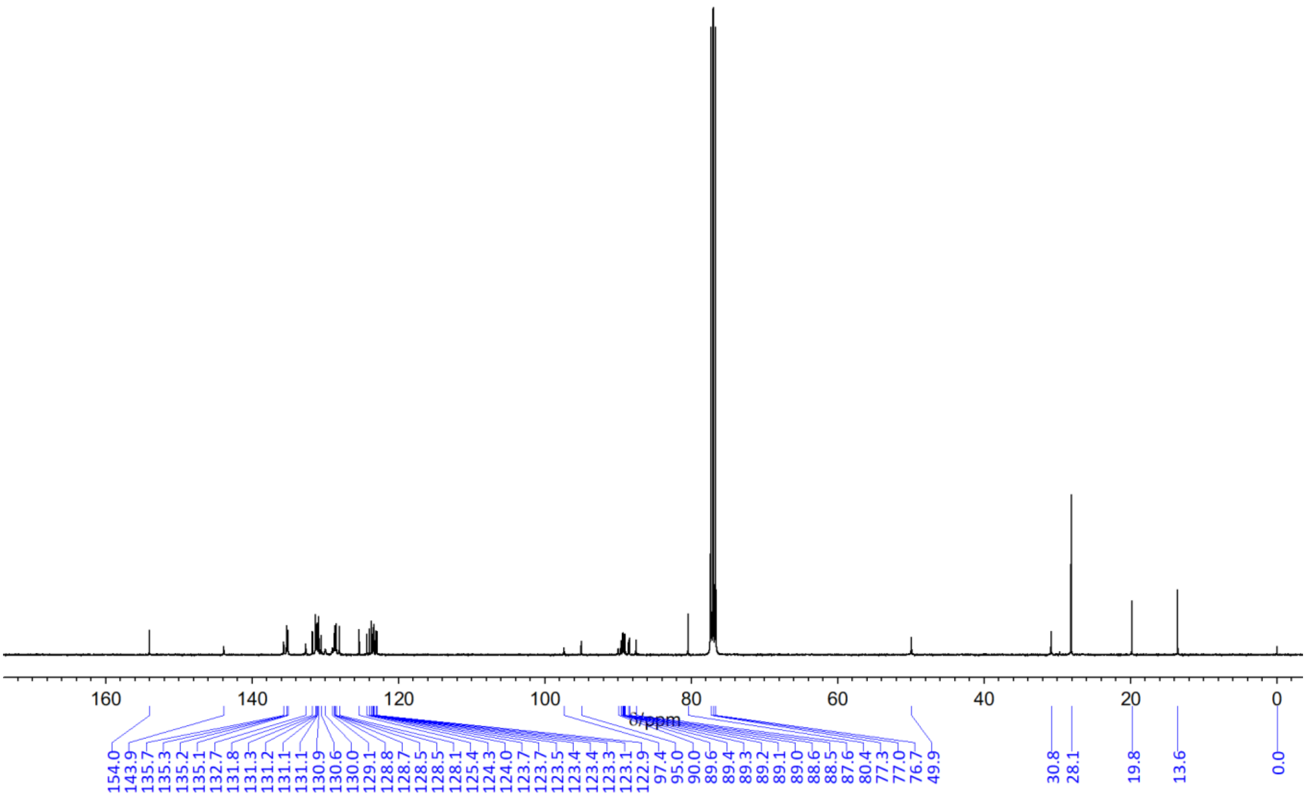

^{13}C NMR spectrum (100 MHz) of *rac*-**4** (11PAM), measured in chloroform-*d* at room temperature.

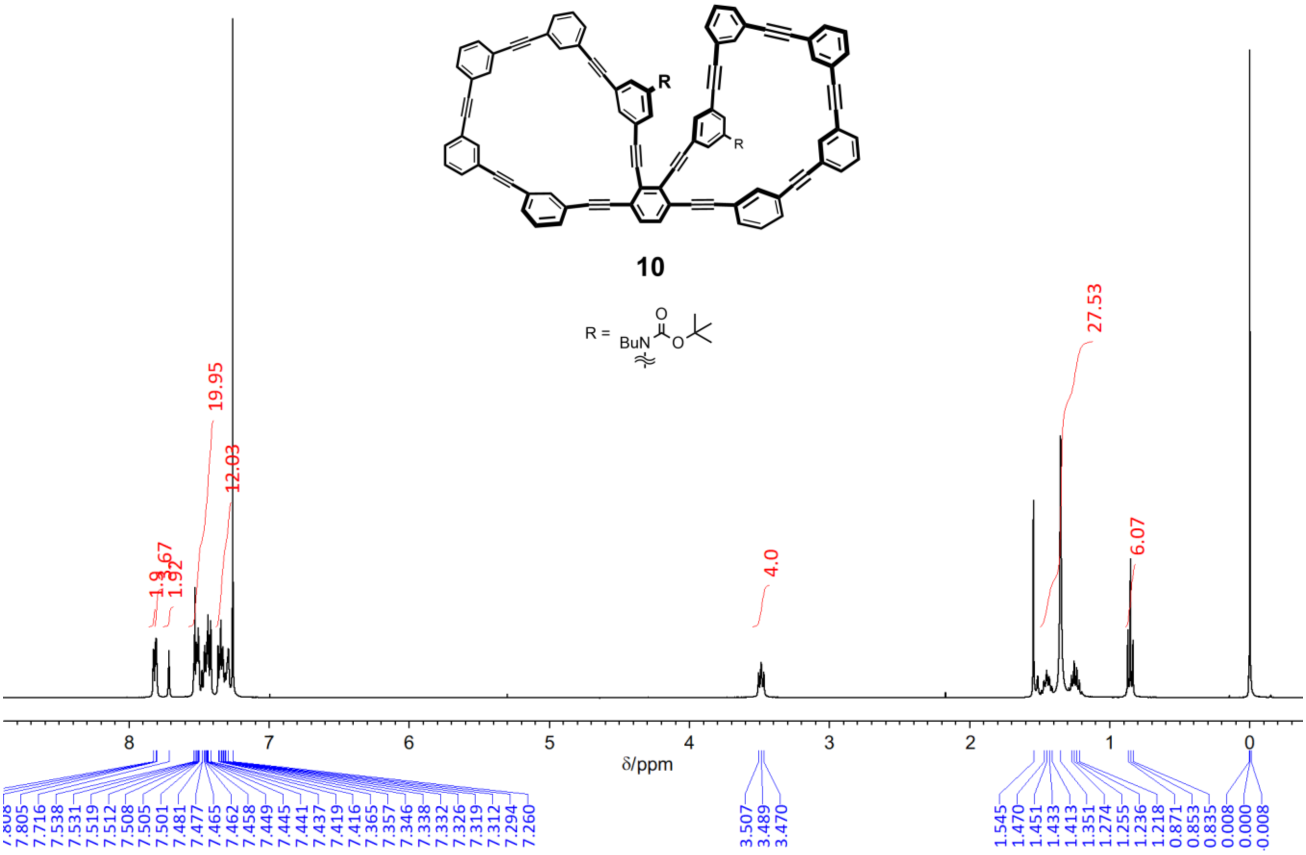

^{1}H NMR spectrum (400 MHz) of **10** (11PAM), measured in chloroform-*d* at room temperature.

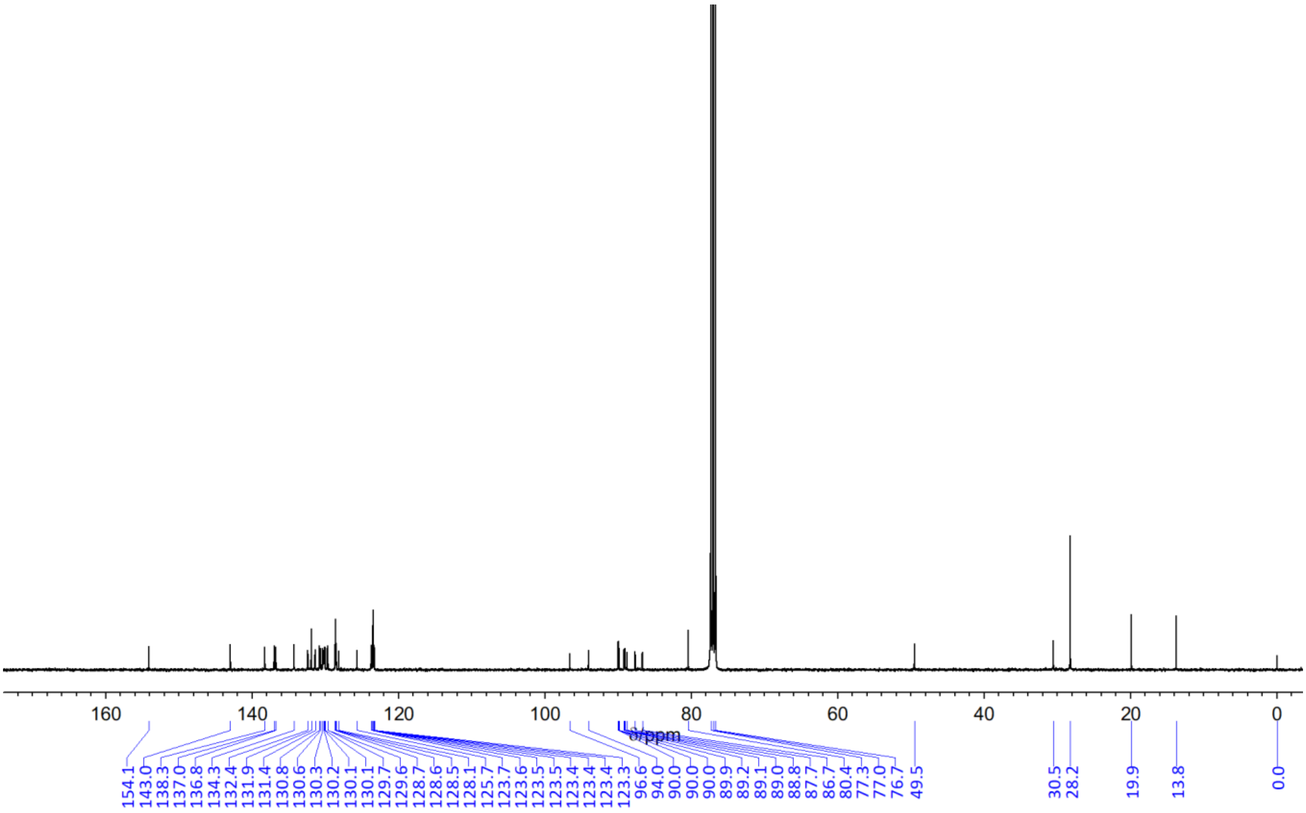

^{13}C NMR spectrum (100 MHz) of **10** (11PAM), measured in chloroform-*d* at room temperature.

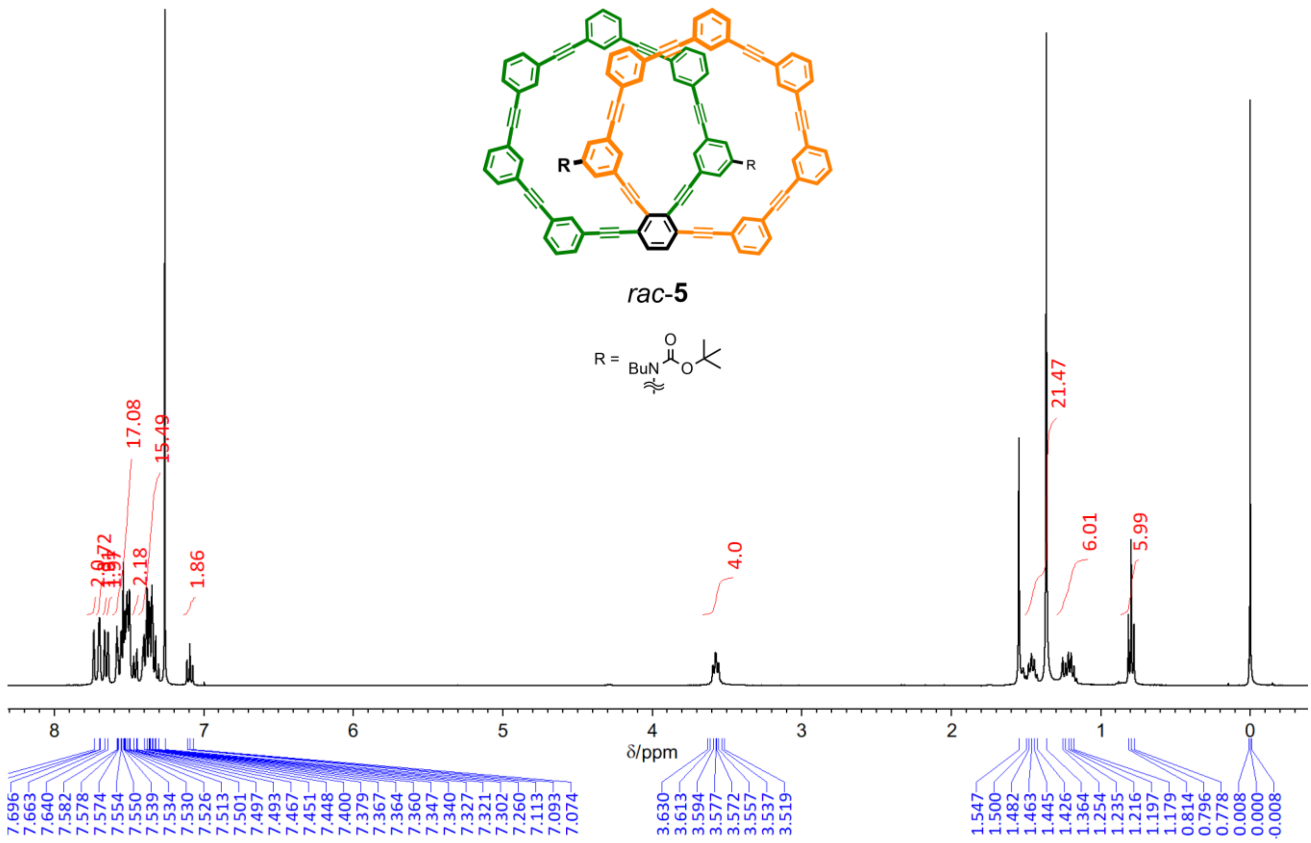

^{1}H NMR spectrum (400 MHz) of *rac*-**5** (13PAM), measured in chloroform-*d* at room temperature.

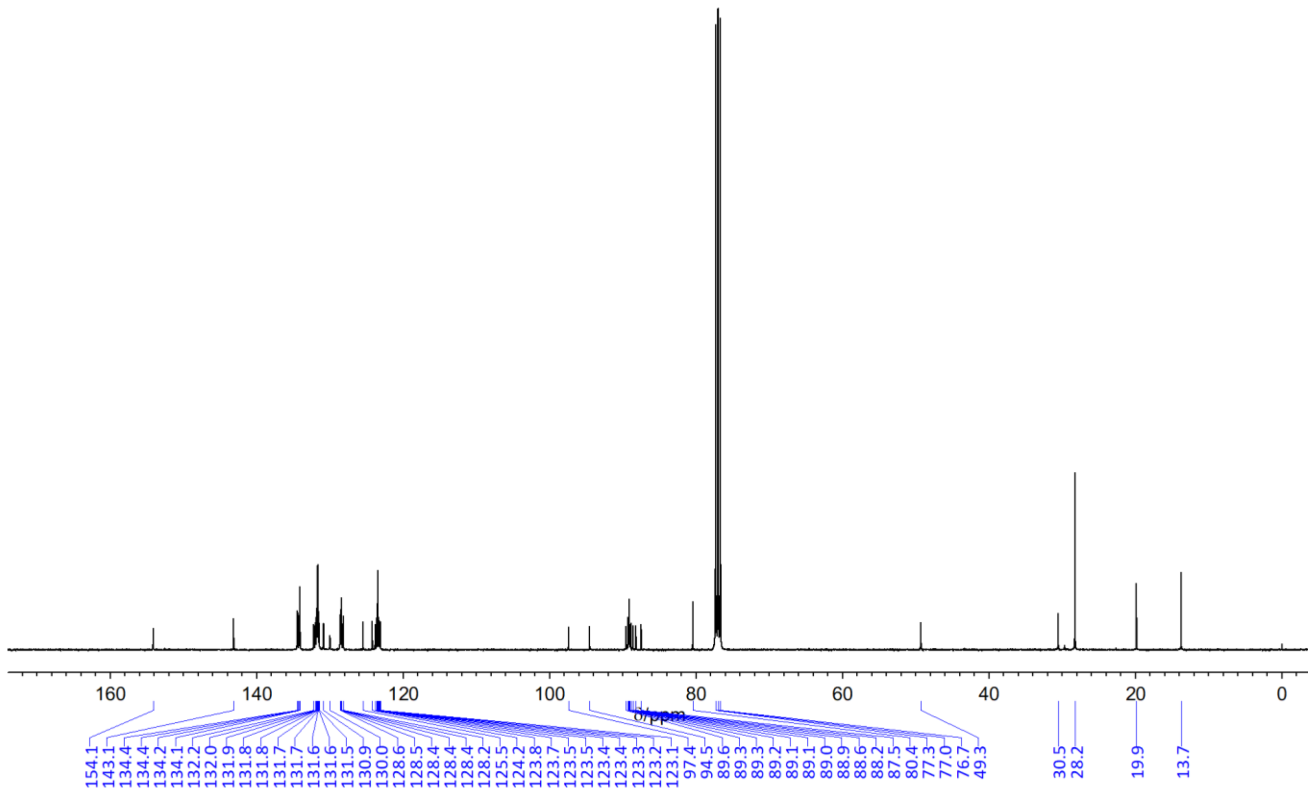

^{13}C NMR spectrum (100 MHz) of *rac*-**5** (13PAM), measured in chloroform-*d* at room temperature.

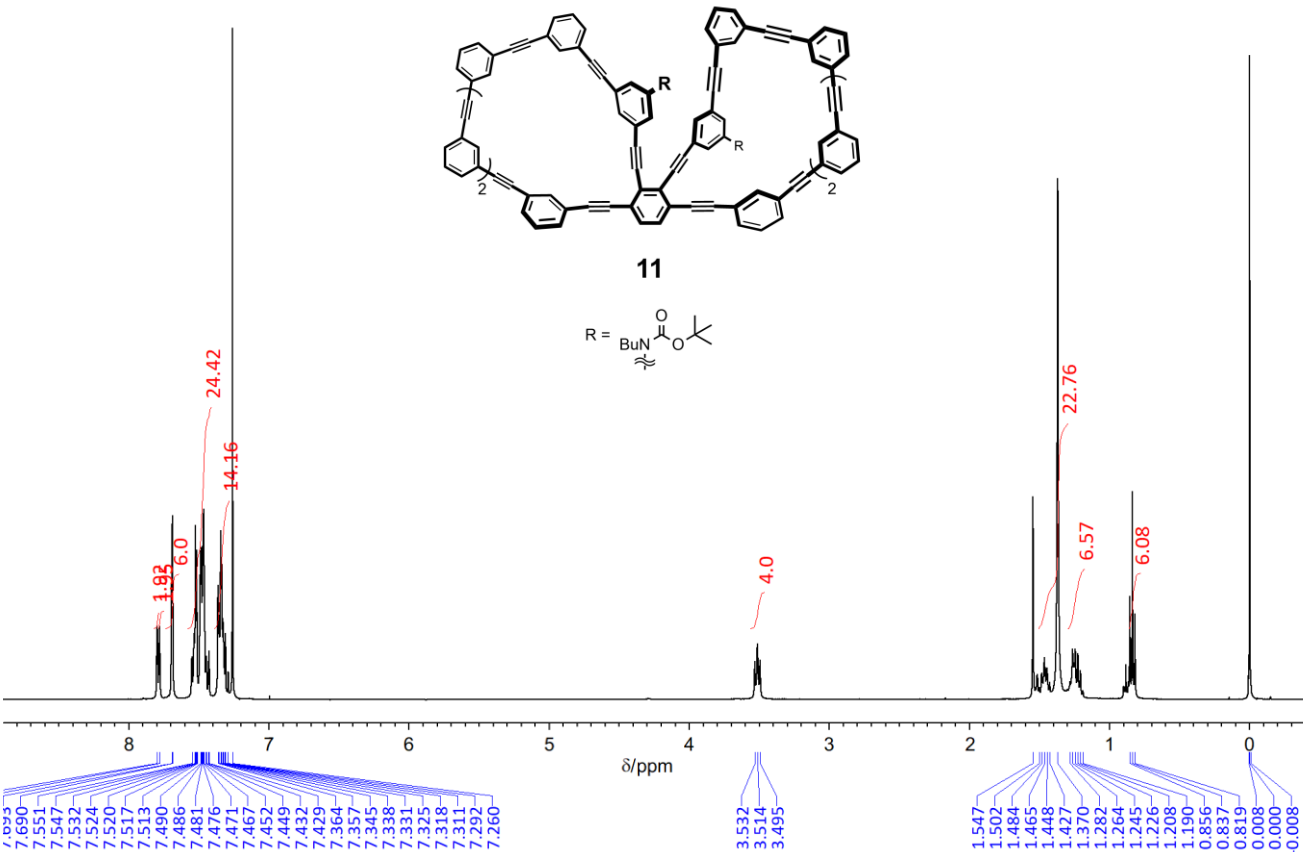

^{1}H NMR spectrum (400 MHz) of **11** (13PAM), cont. residual hexane, measured in chloroform-*d* at room temperature.

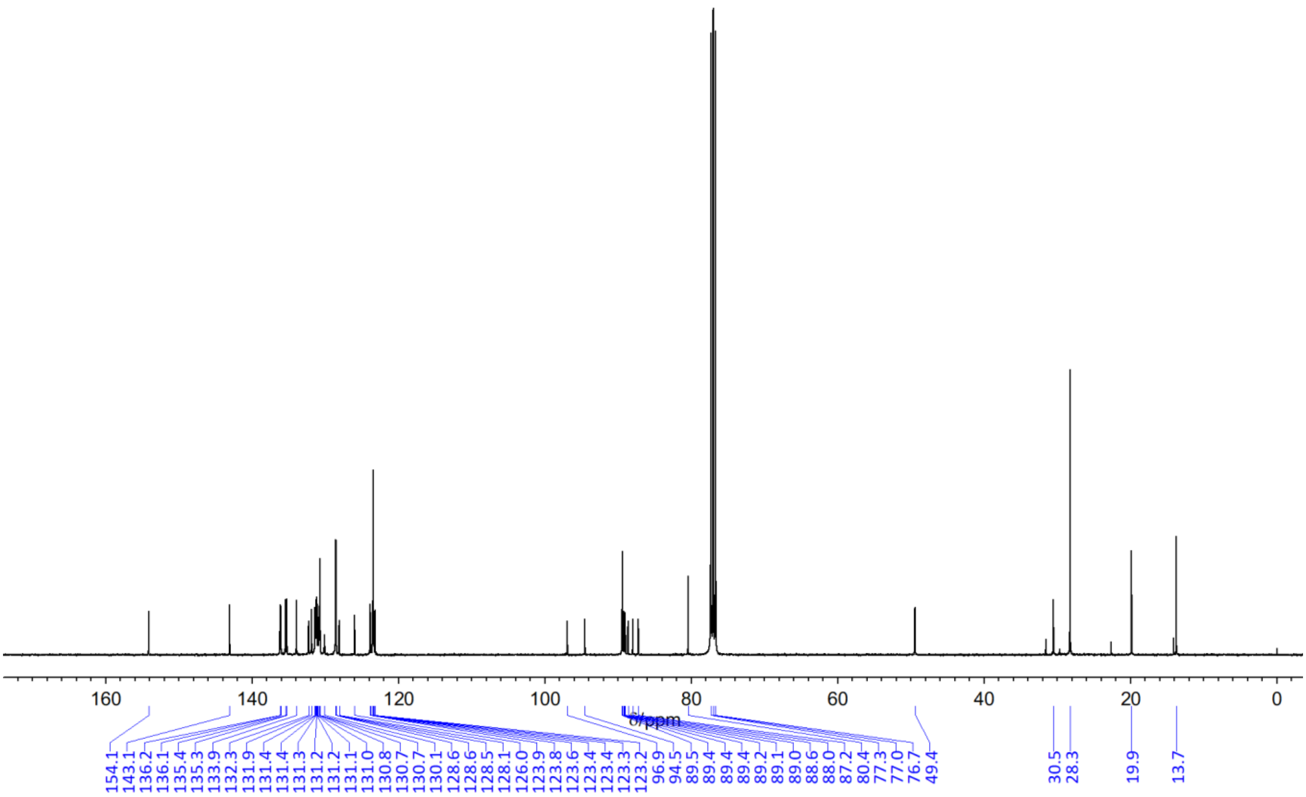

^{13}C NMR spectrum (100 MHz) of **11** (13PAM), cont. residual hexane, measured in chloroform-*d* at room temperature.

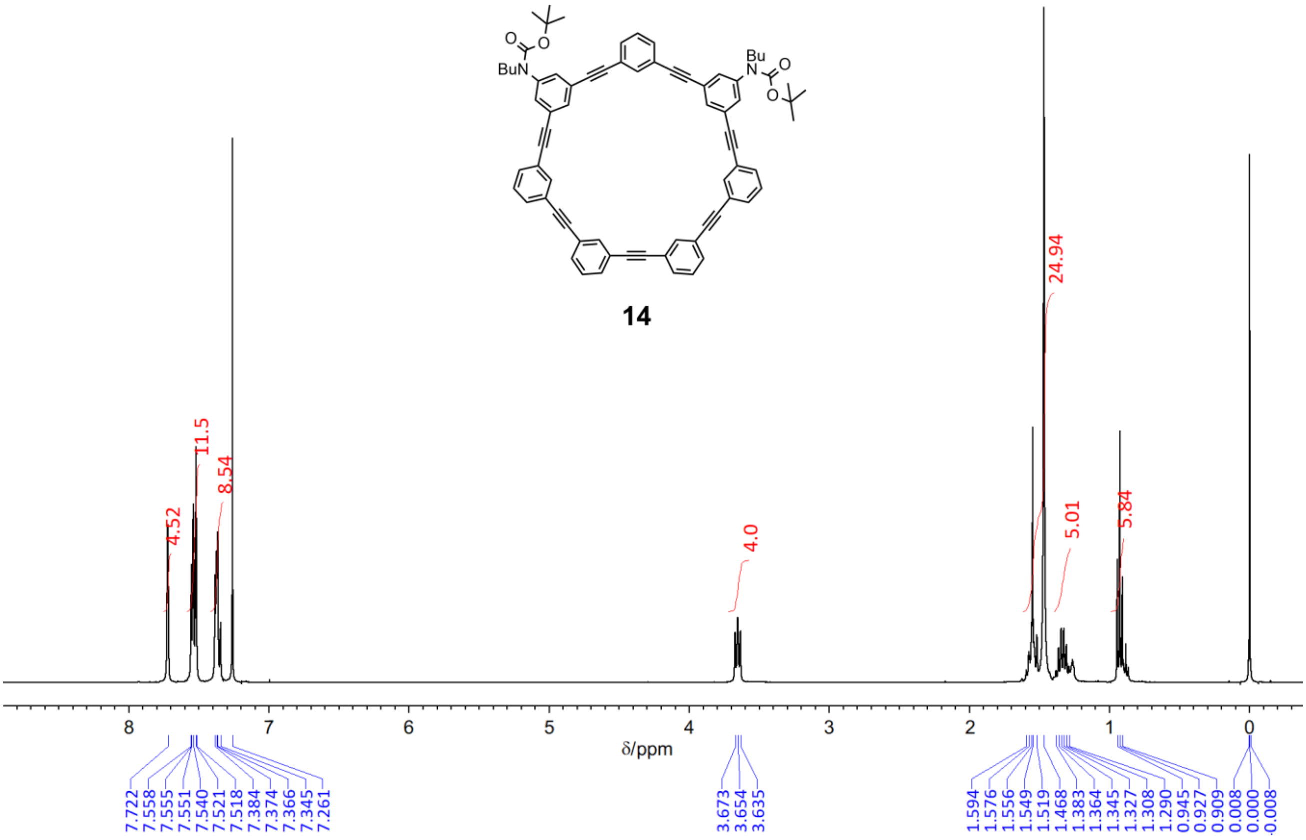

^{1}H NMR spectrum (400 MHz) of **14** (7PAM), cont. residual hexane, measured in chloroform-*d* at room temperature.

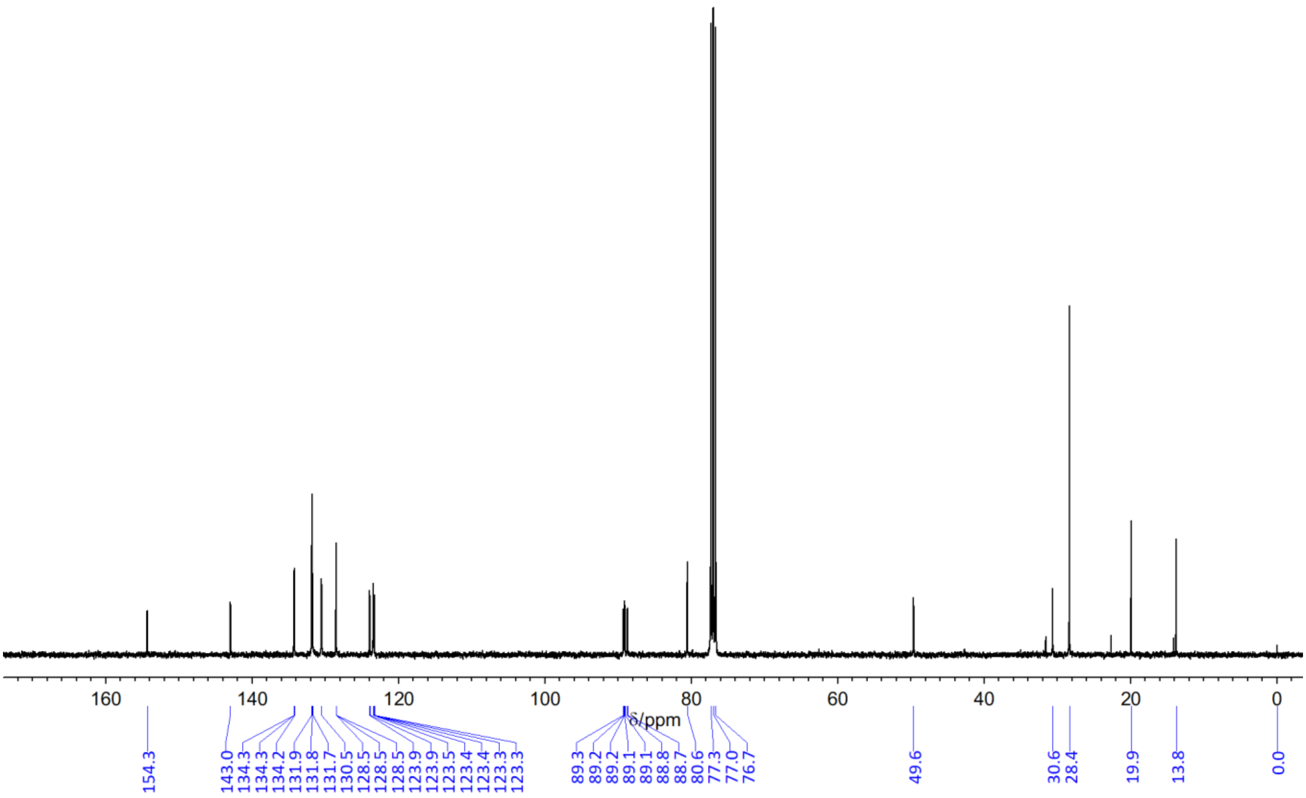

^{13}C NMR spectrum (100 MHz) of **14** (7PAM), cont. residual hexane, measured in chloroform-*d* at room temperature.

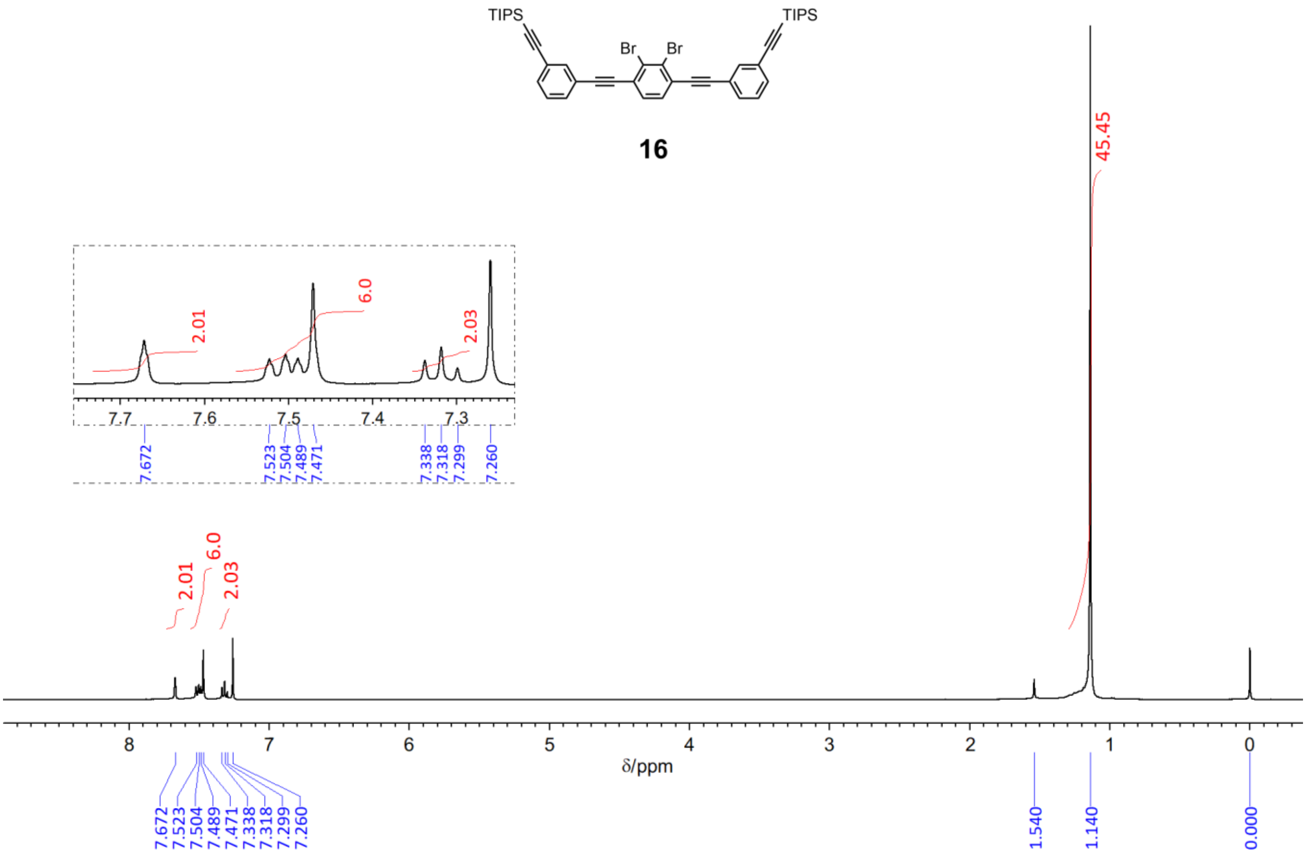

^{1}H NMR spectrum (400 MHz) of **16**, measured in chloroform-*d* at room temperature.

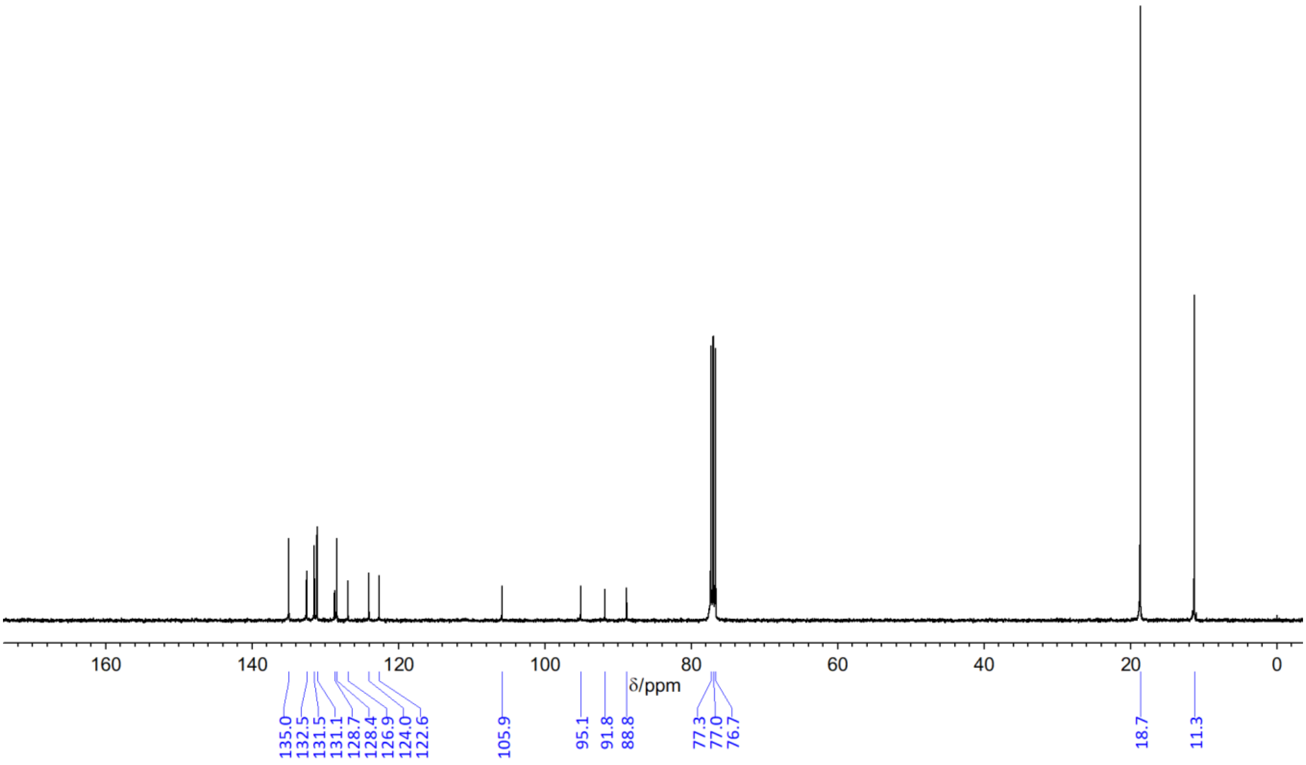

^{13}C NMR spectrum (100 MHz) of **16**, measured in chloroform-*d* at room temperature.

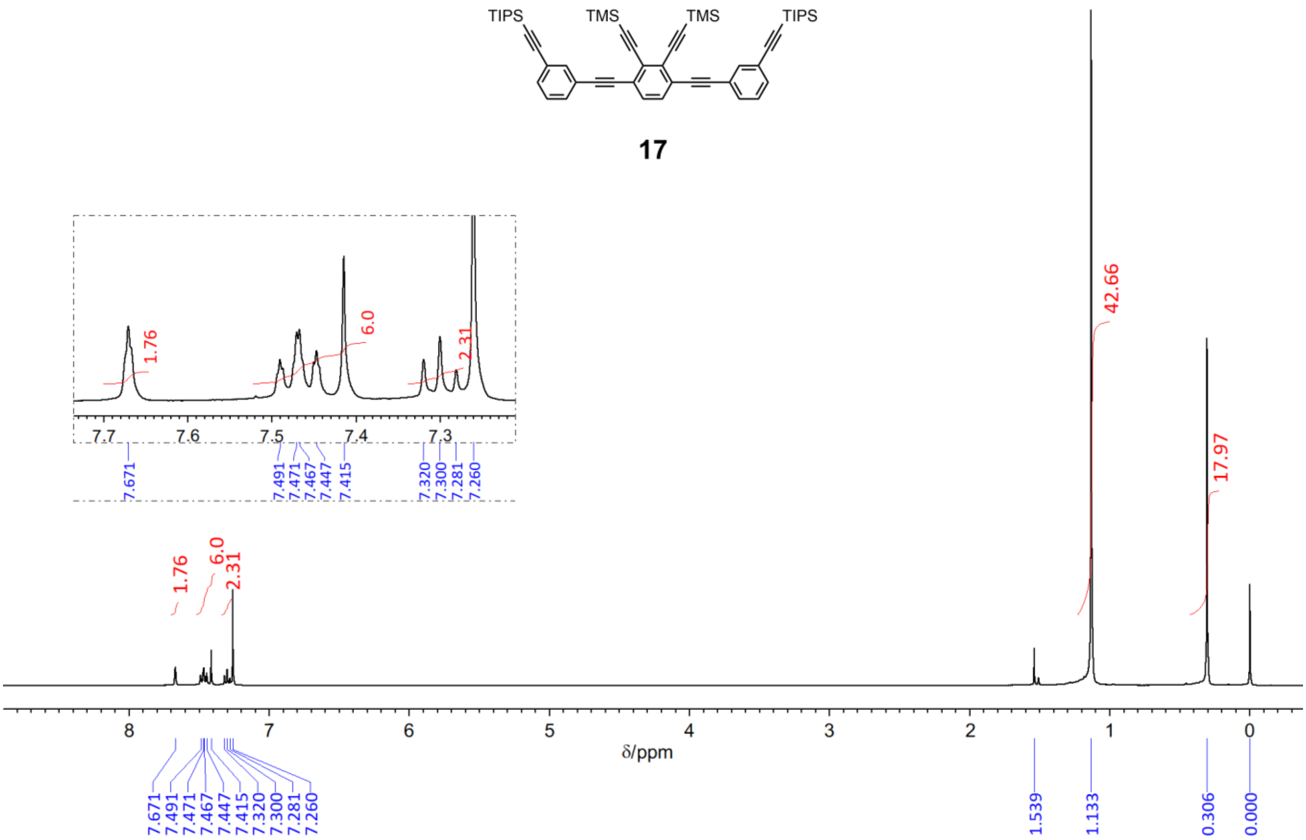

^{1}H NMR spectrum (400 MHz) of **17**, measured in chloroform-*d* at room temperature.

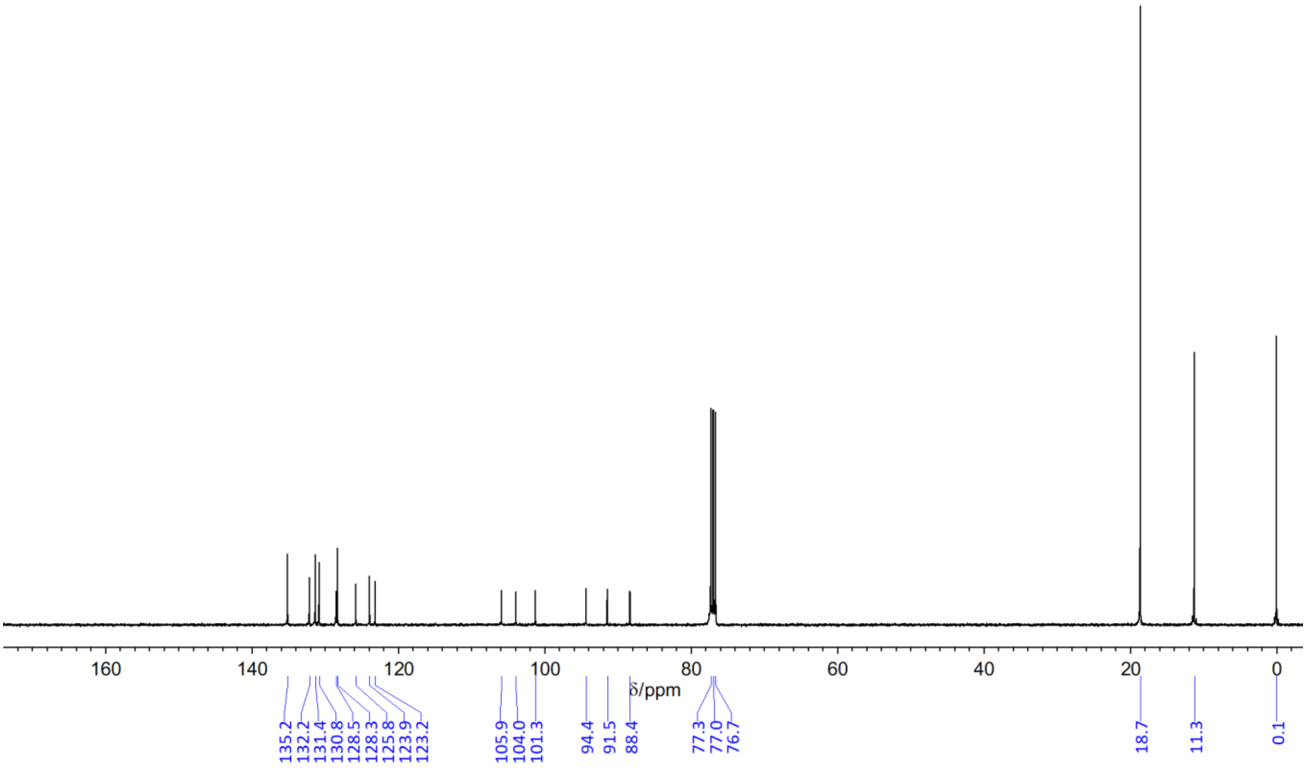

^{13}C NMR spectrum (100 MHz) of **17**, measured in chloroform-*d* at room temperature.

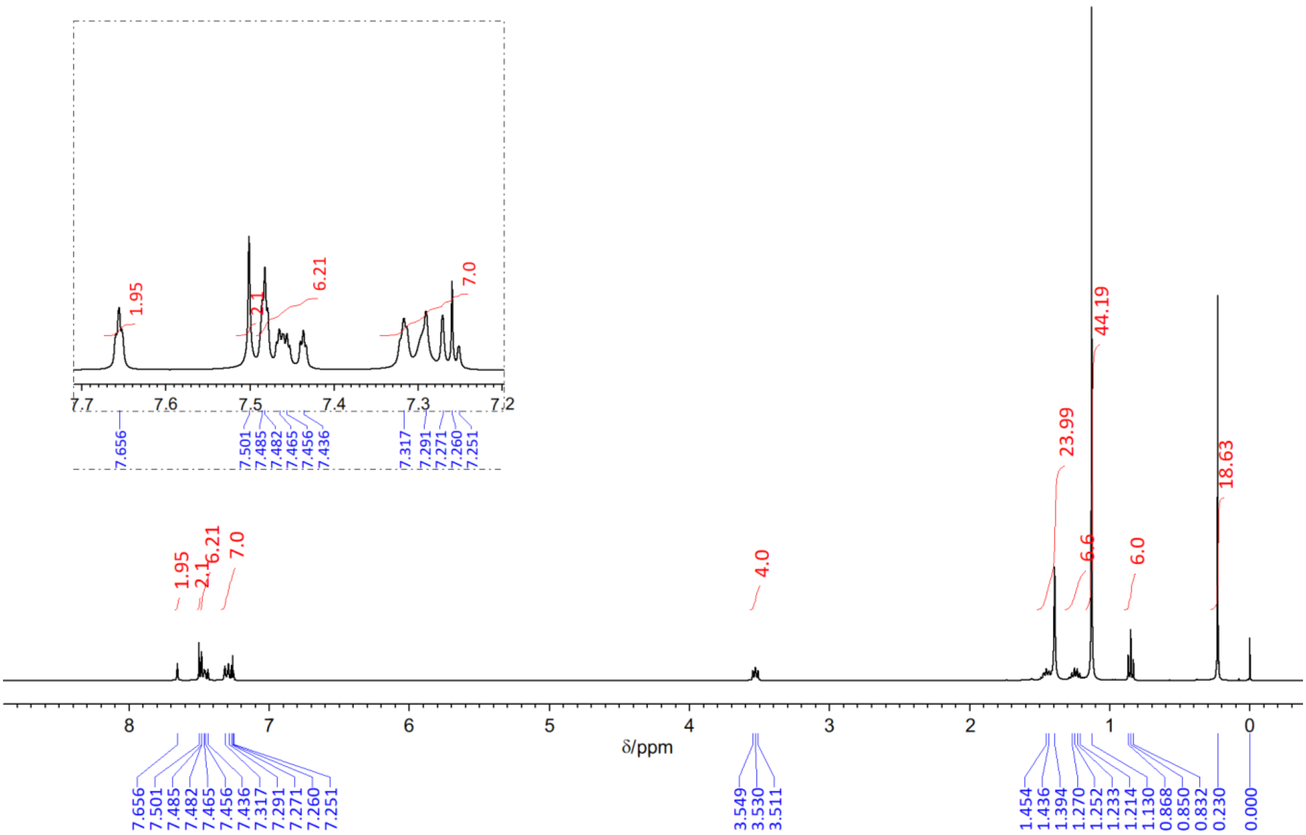

^{1}H NMR spectrum (400 MHz) of **18**, measured in chloroform-*d* at room temperature.

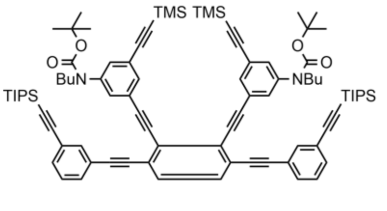

18

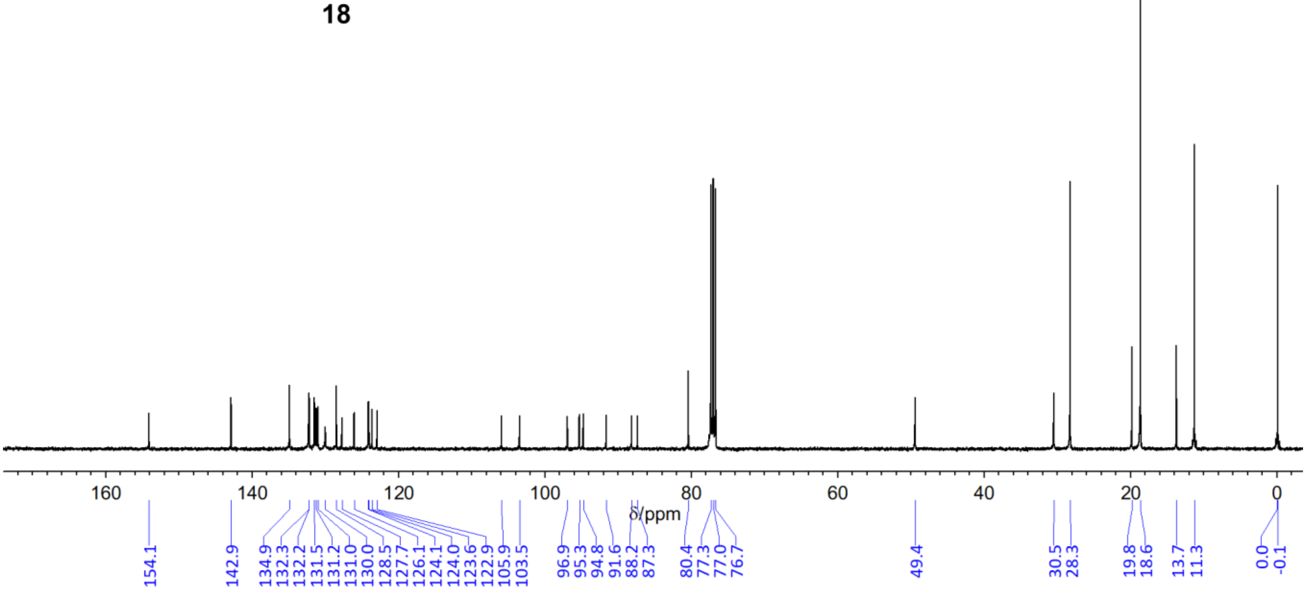

^{13}C NMR spectrum (100 MHz) of **18**, measured in chloroform-*d* at room temperature.

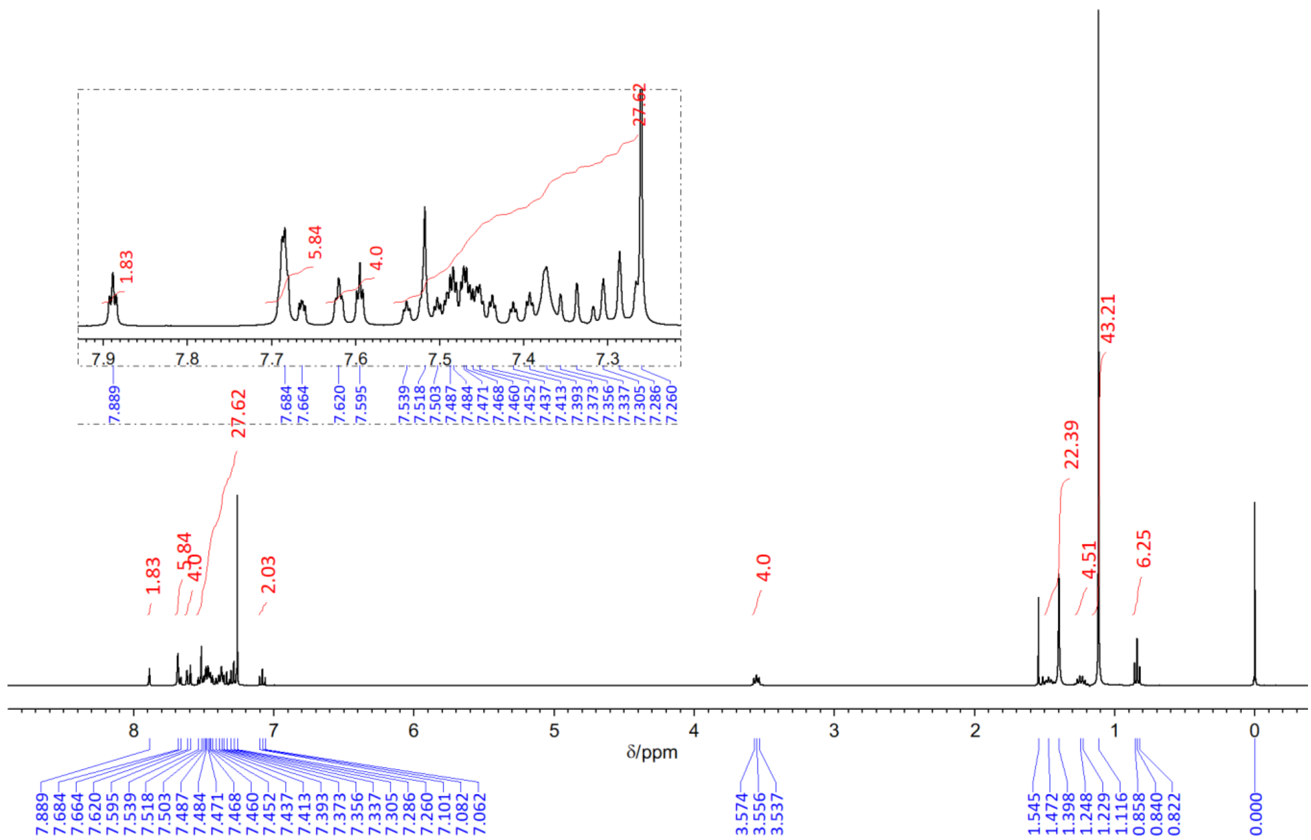

^{1}H NMR spectrum (400 MHz) of **19**, measured in chloroform-*d* at room temperature.

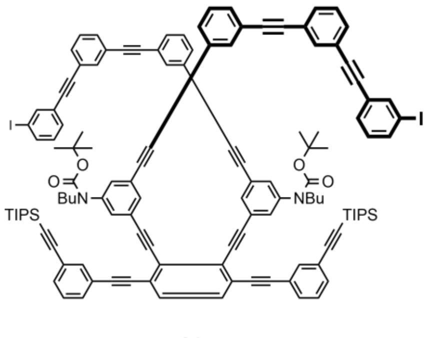

19

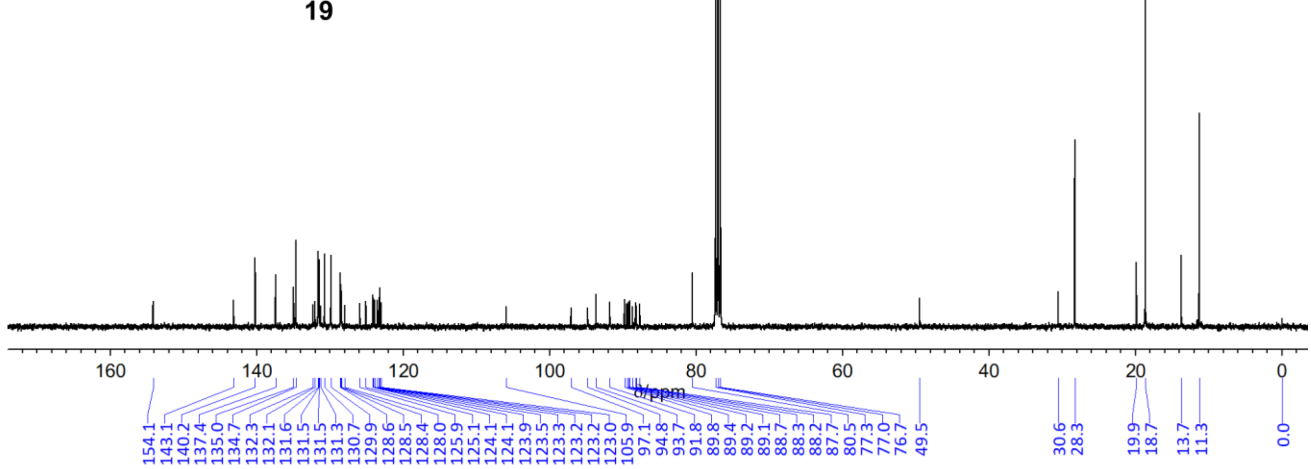

^{13}C NMR spectrum (100 MHz) of **19**, measured in chloroform-*d* at room temperature.

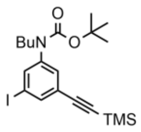

21

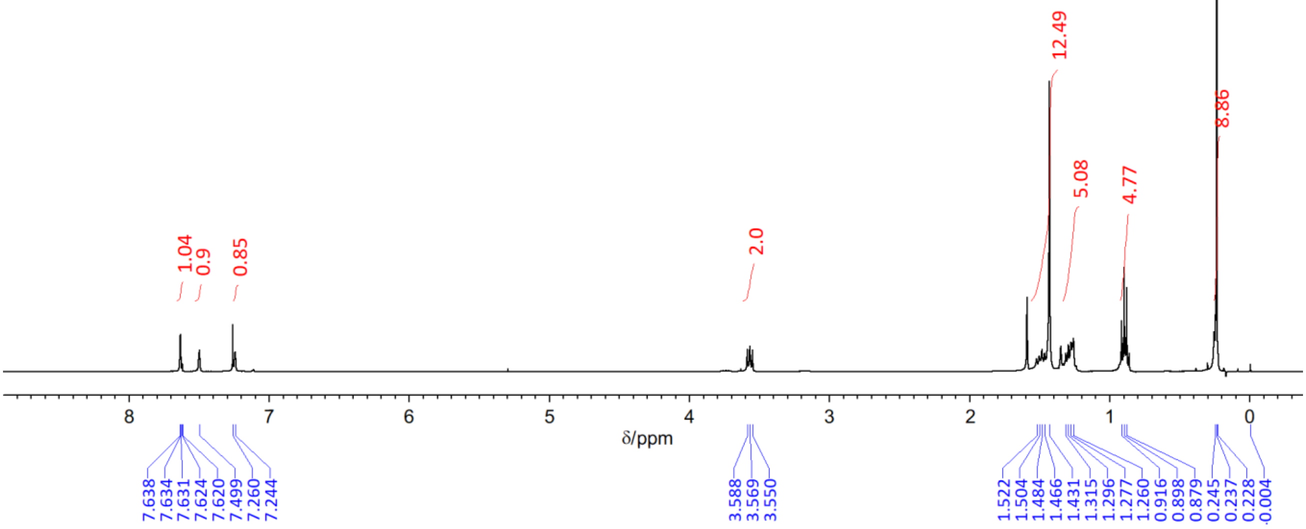

^{1}H NMR spectrum (400 MHz) of **21** (incomplete purification), measured in chloroform-*d* at room temperature.

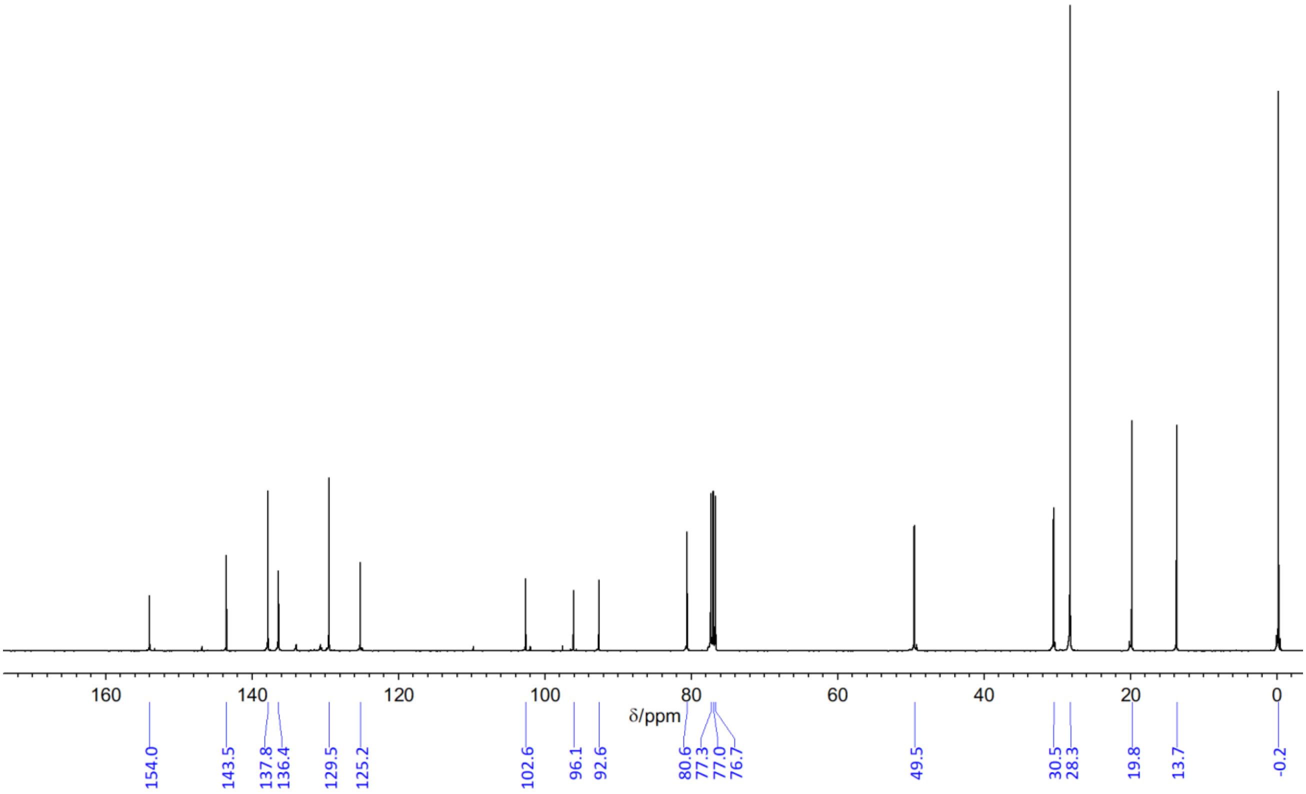

^{13}C NMR spectrum (100 MHz) of **21** (incomplete purification), measured in chloroform-*d* at room temperature.

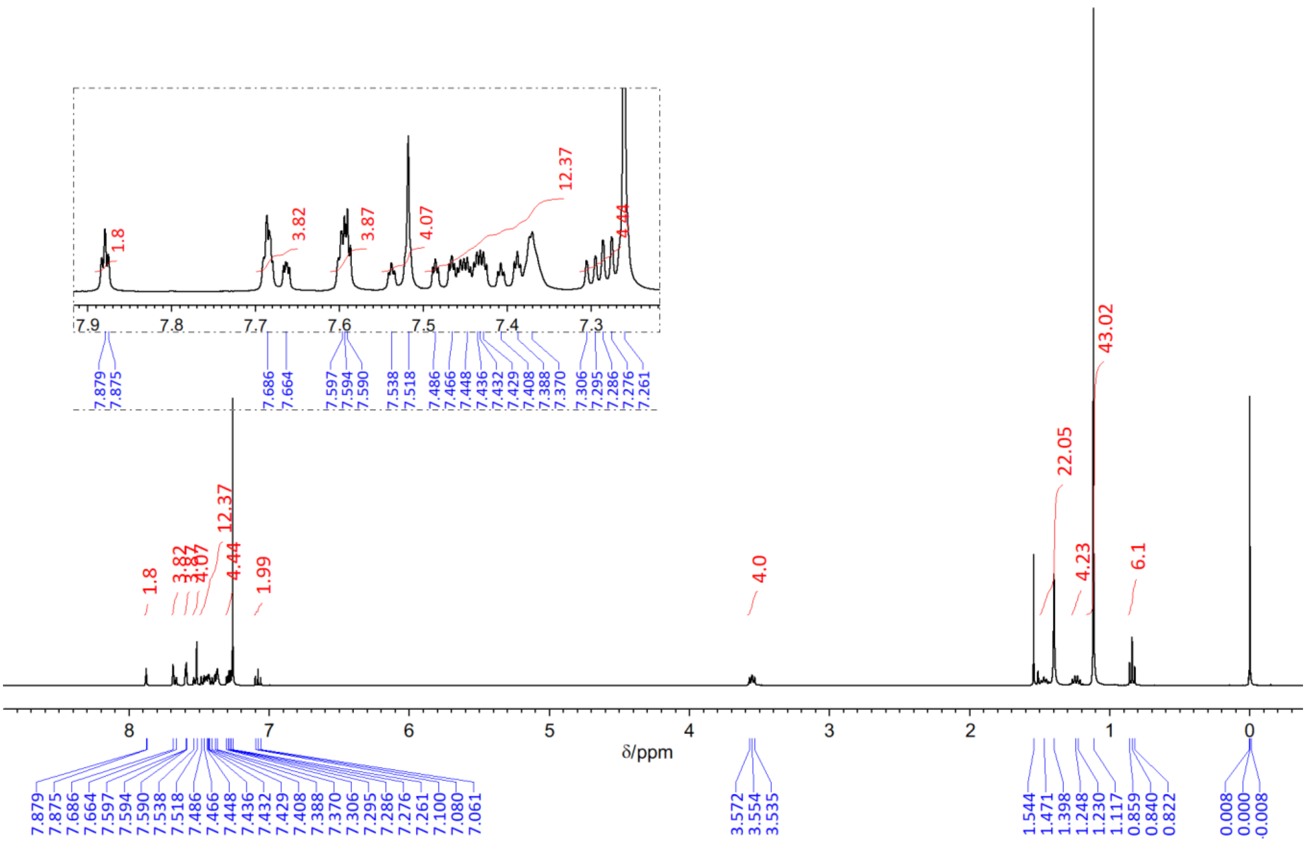

^{1}H NMR spectrum (400 MHz) of **24**, measured in chloroform-*d* at room temperature.

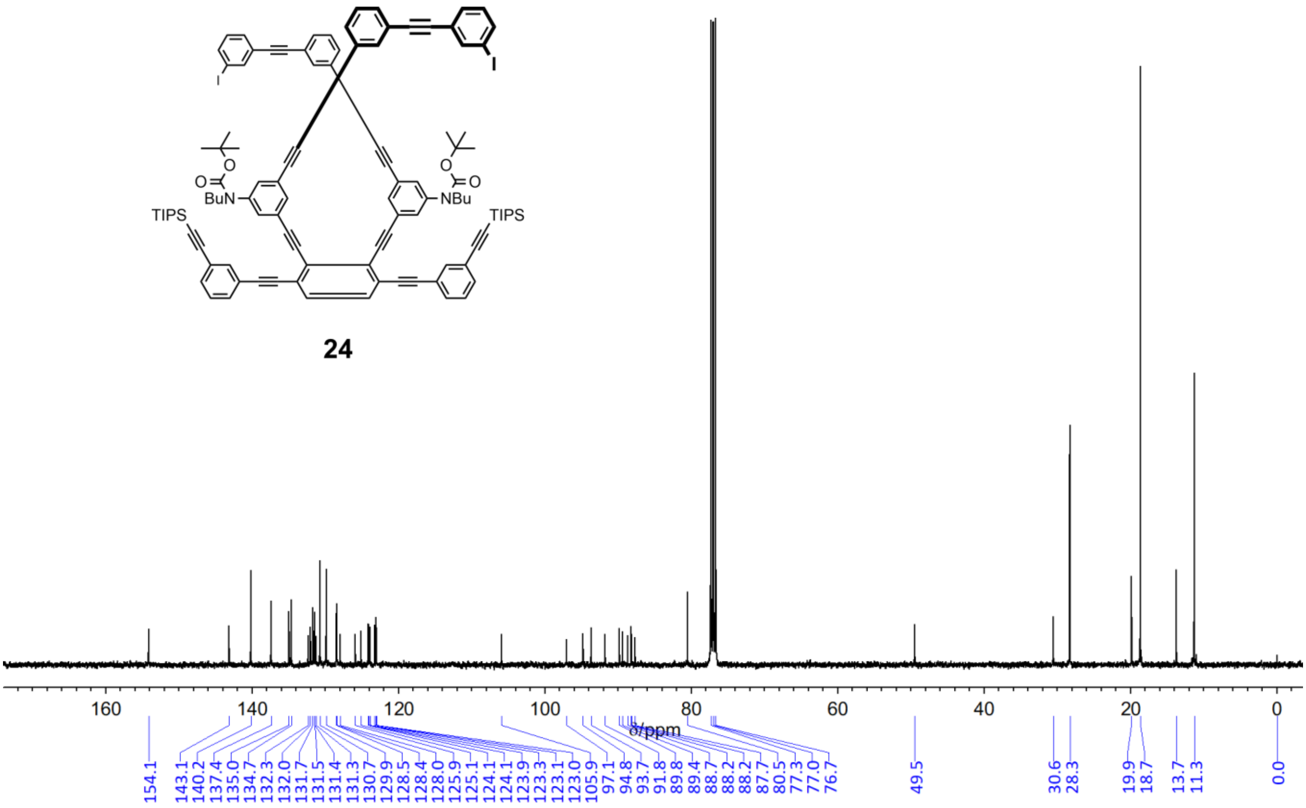

24

^{13}C NMR spectrum (100 MHz) of **24**, measured in chloroform-*d* at room temperature.

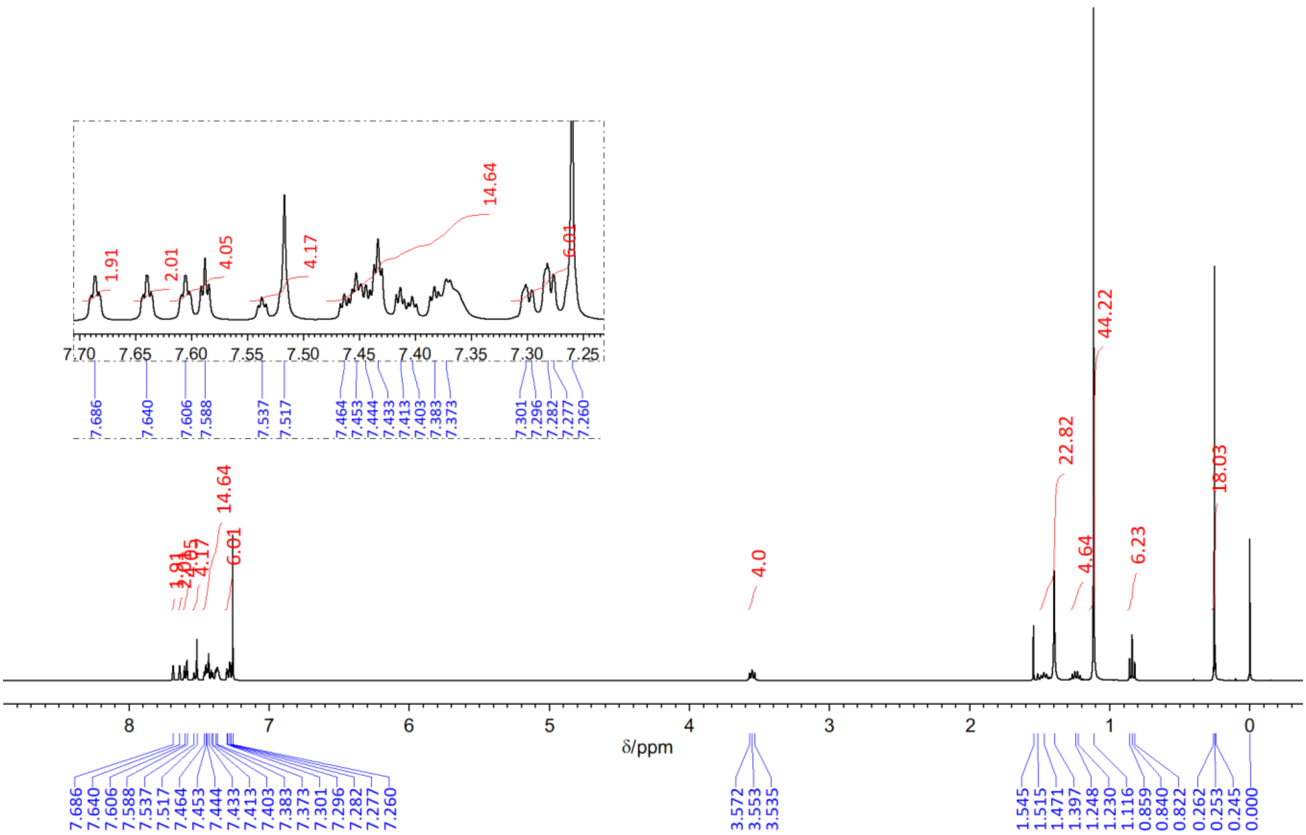

^{1}H NMR spectrum (400 MHz) of **25**, measured in chloroform-*d* at room temperature.

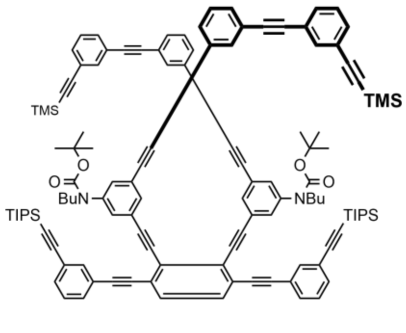

25

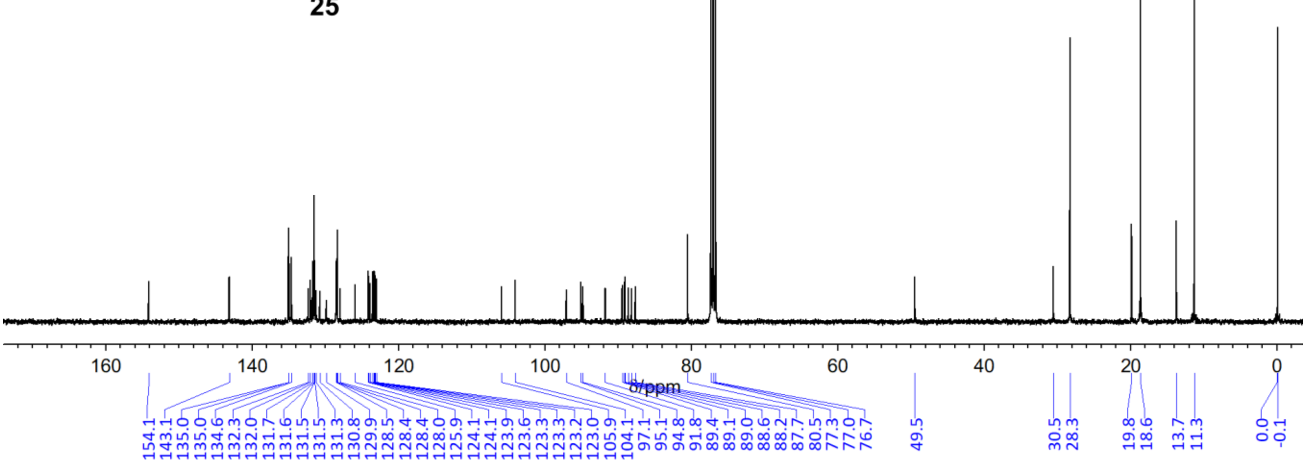

^{13}C NMR spectrum (100 MHz) of **25**, measured in chloroform-*d* at room temperature.

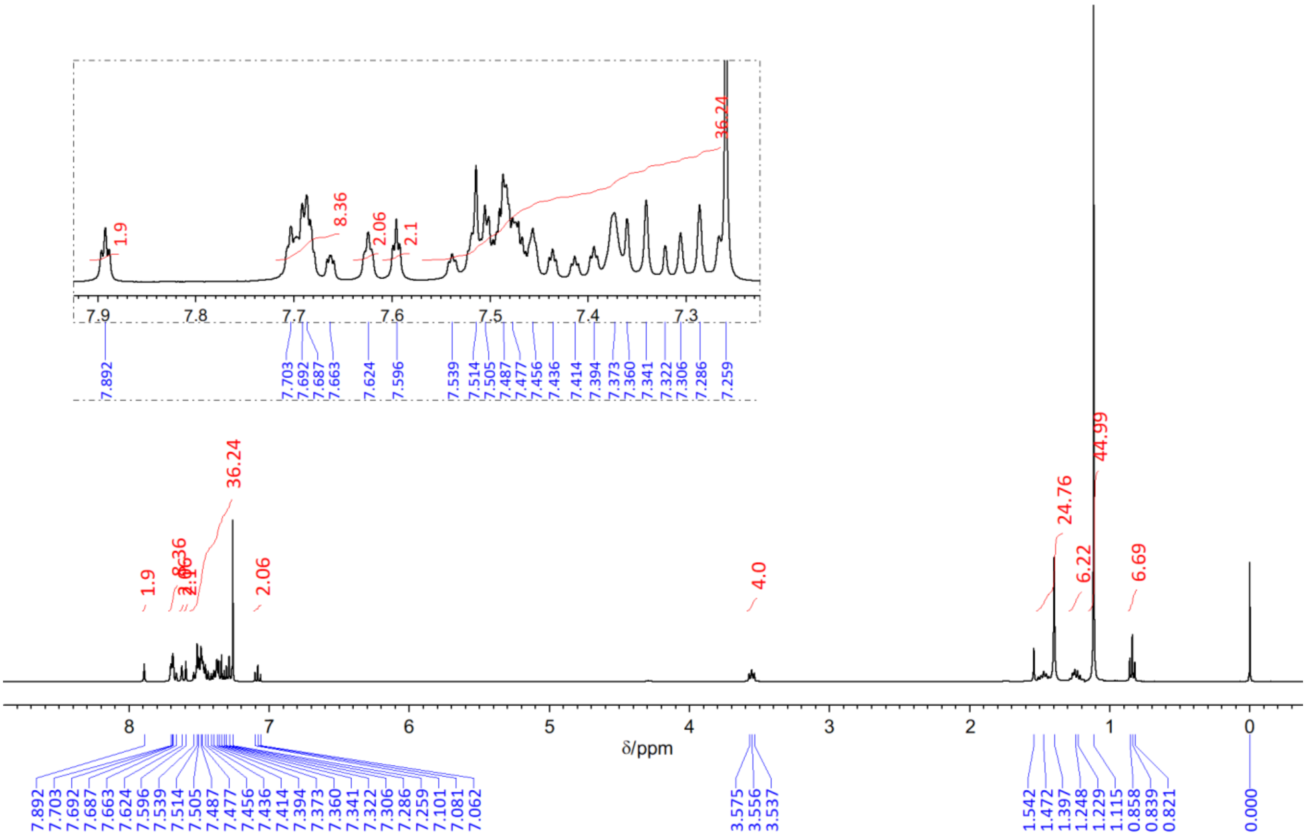

^{1}H NMR spectrum (400 MHz) of **26**, measured in chloroform-*d* at room temperature.

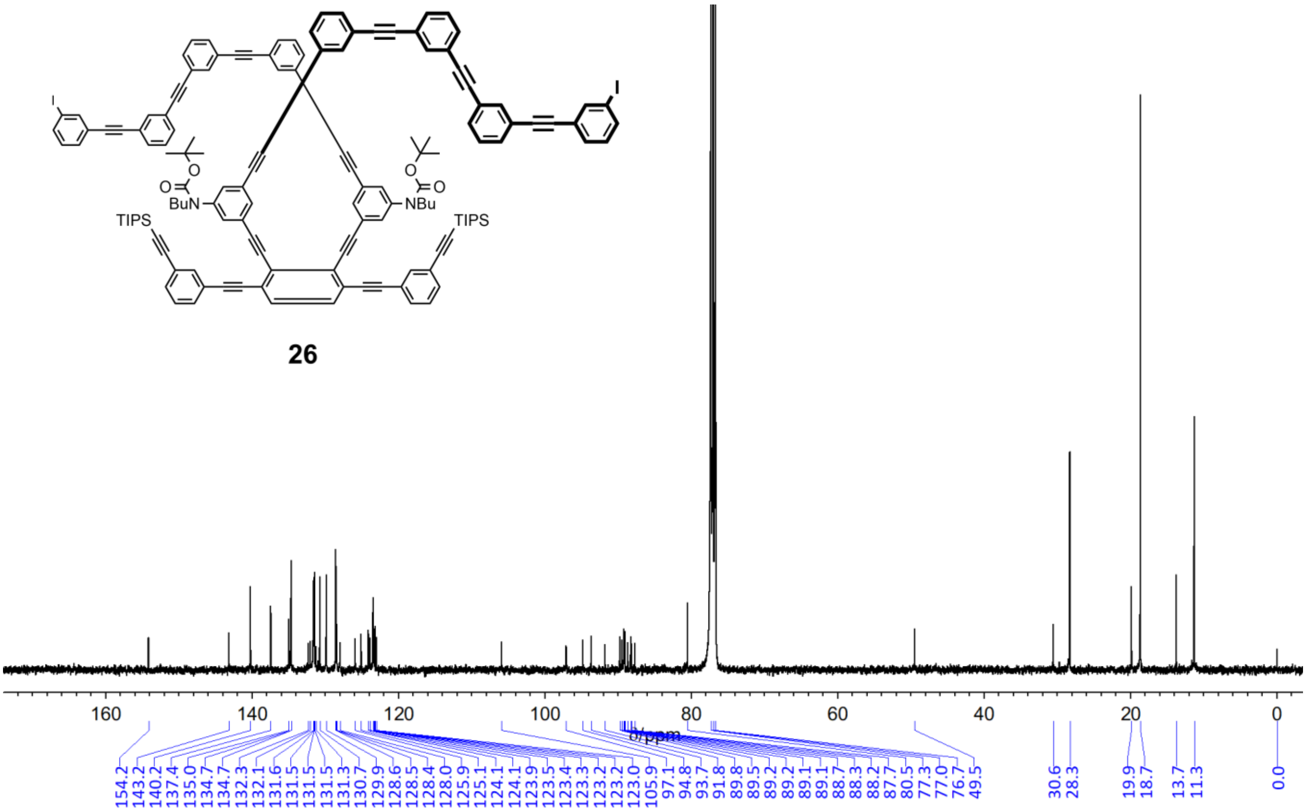

^{13}C NMR spectrum (100 MHz) of **26**, measured in chloroform-*d* at room temperature.

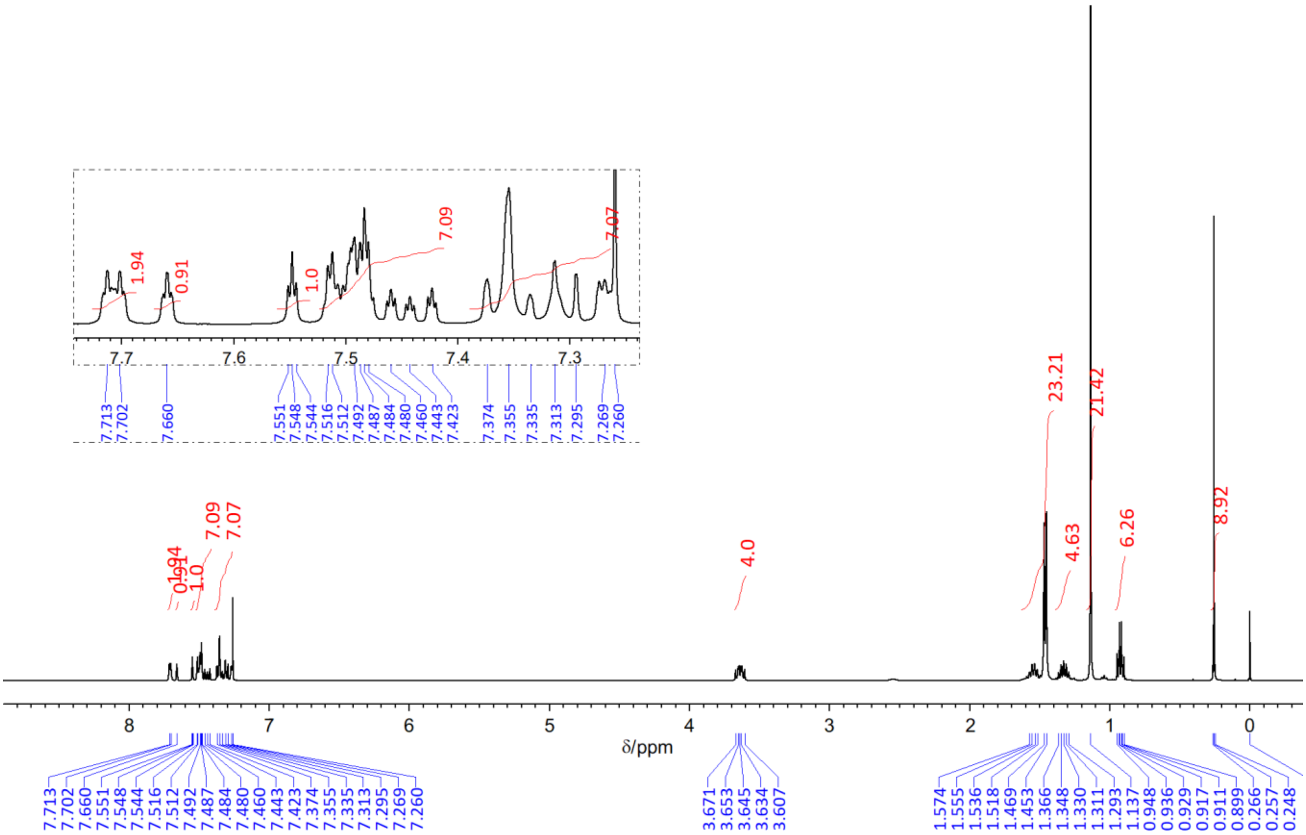

^{1}H NMR spectrum (400 MHz) of **29**, measured in chloroform-*d* at room temperature.

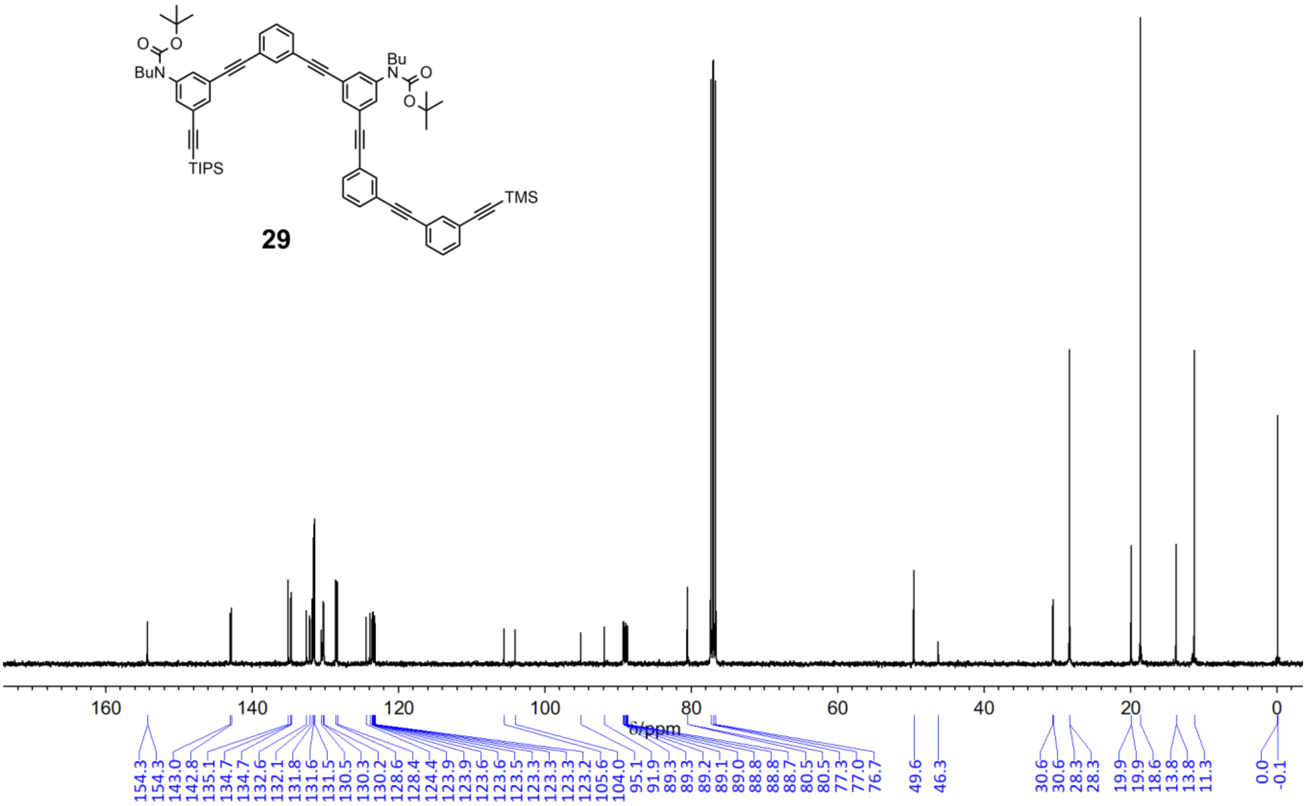

^{13}C NMR spectrum (100 MHz) of **29**, measured in chloroform-*d* at room temperature.

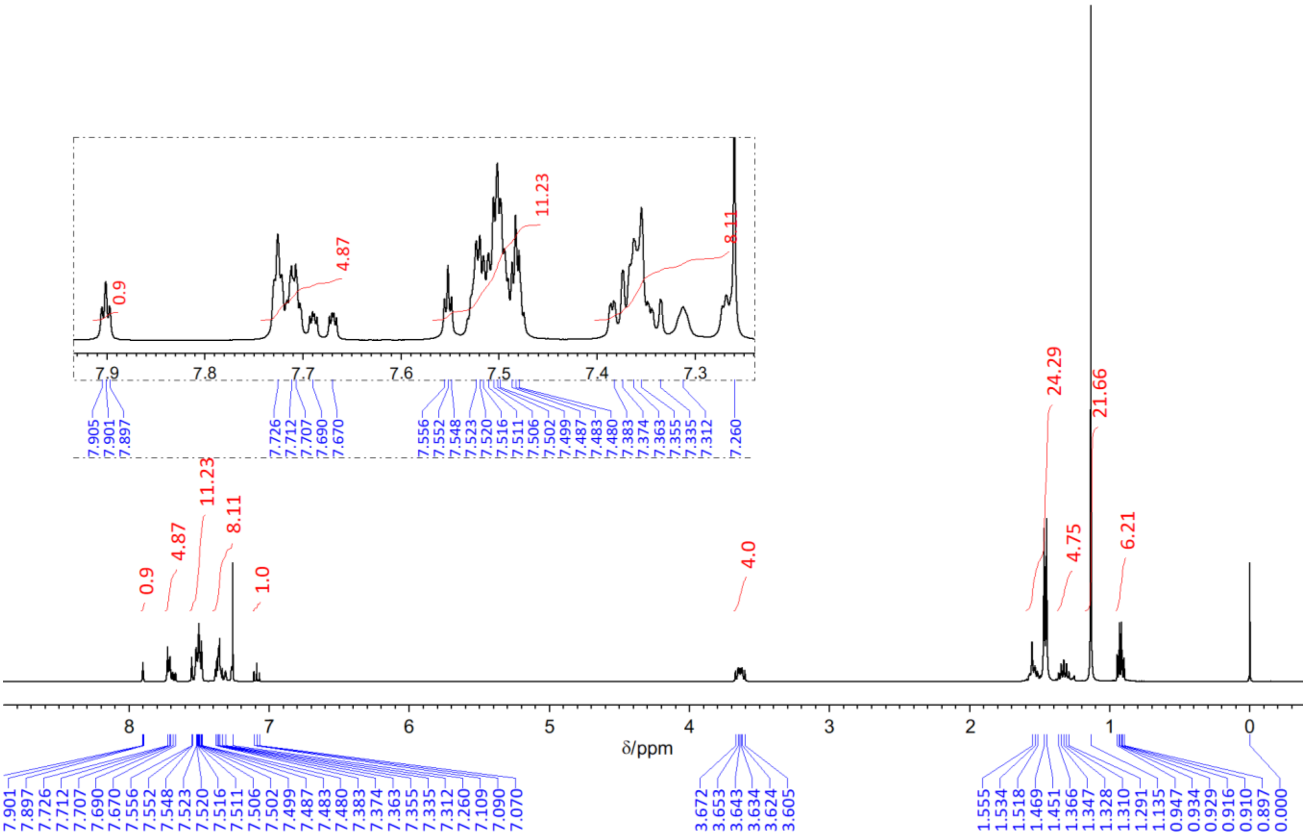

^{1}H NMR spectrum (400 MHz) of **30**, measured in chloroform-*d* at room temperature.

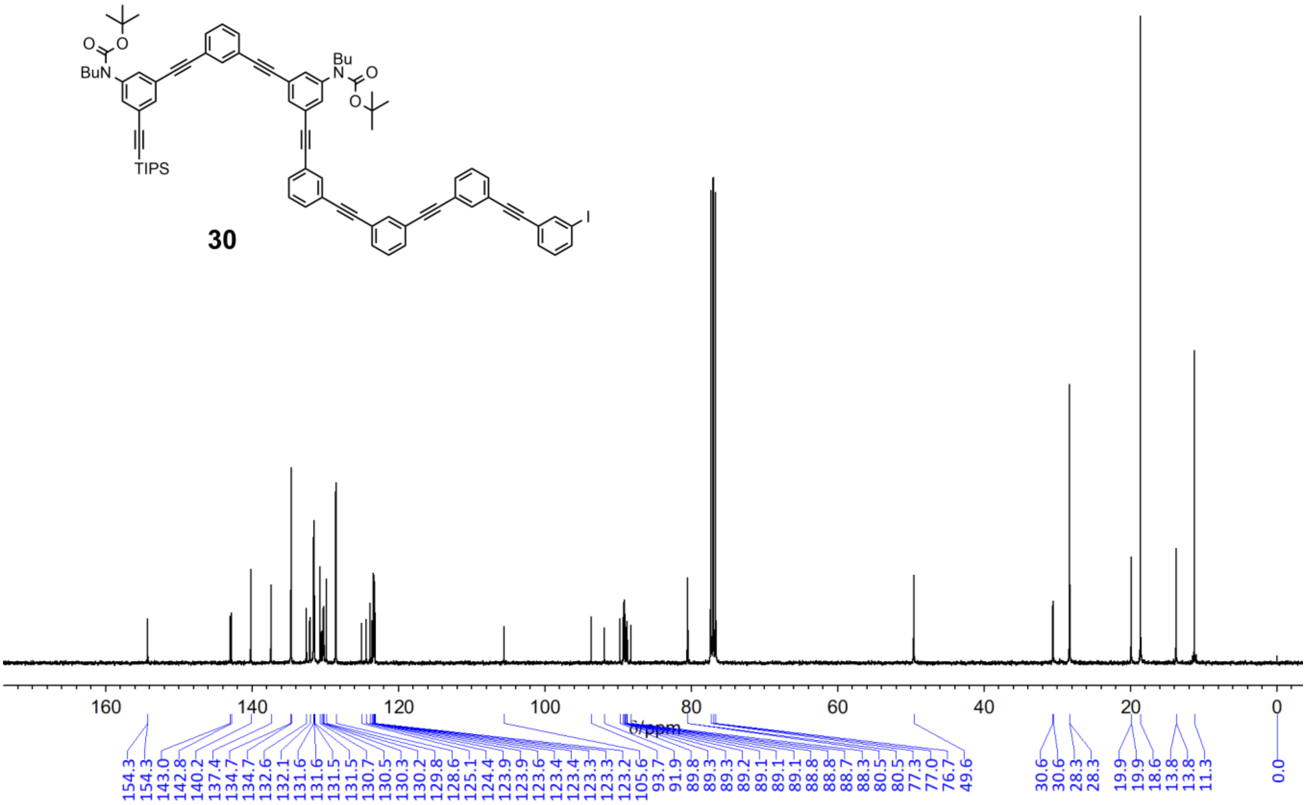

^{13}C NMR spectrum (100 MHz) of **30**, measured in chloroform-*d* at room temperature.

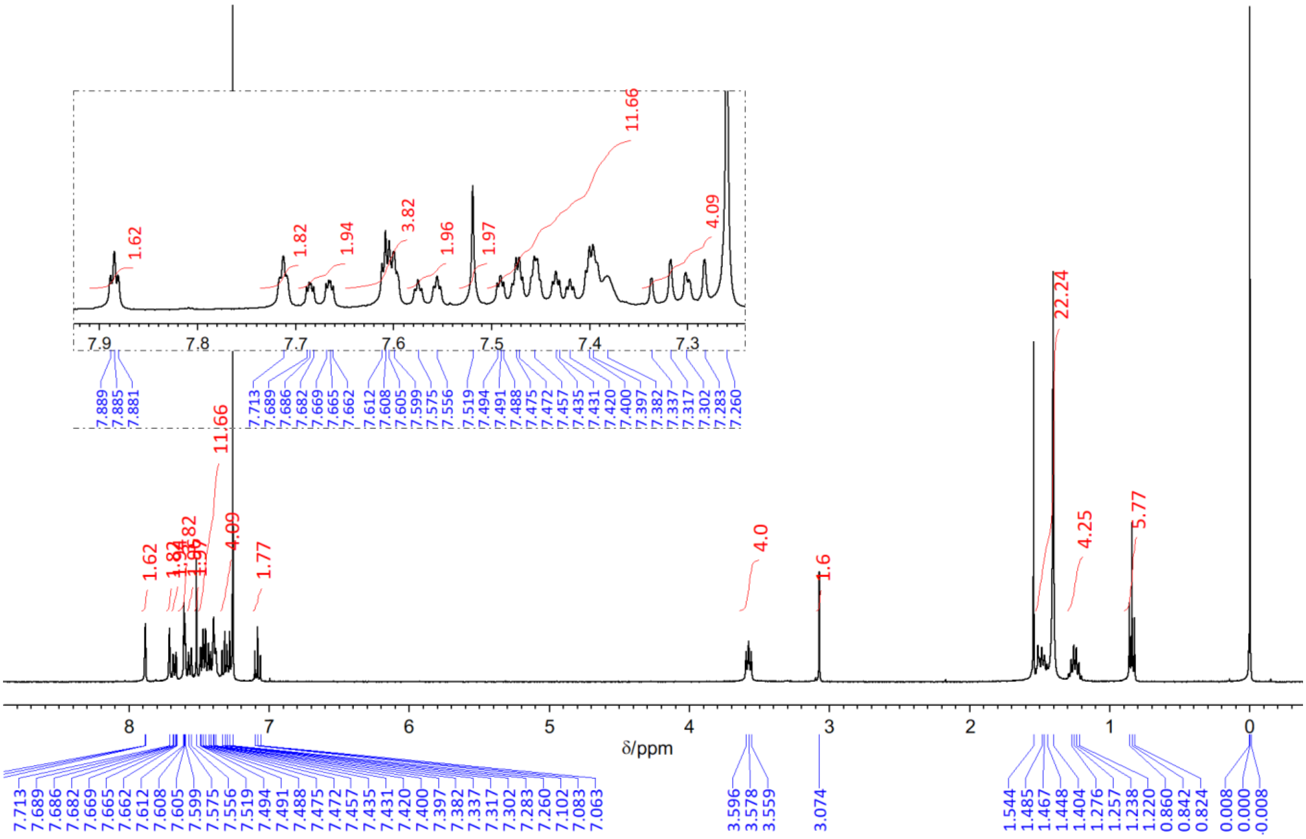

^{1}H NMR spectrum (400 MHz) of **6**, measured in chloroform-*d* at room temperature.

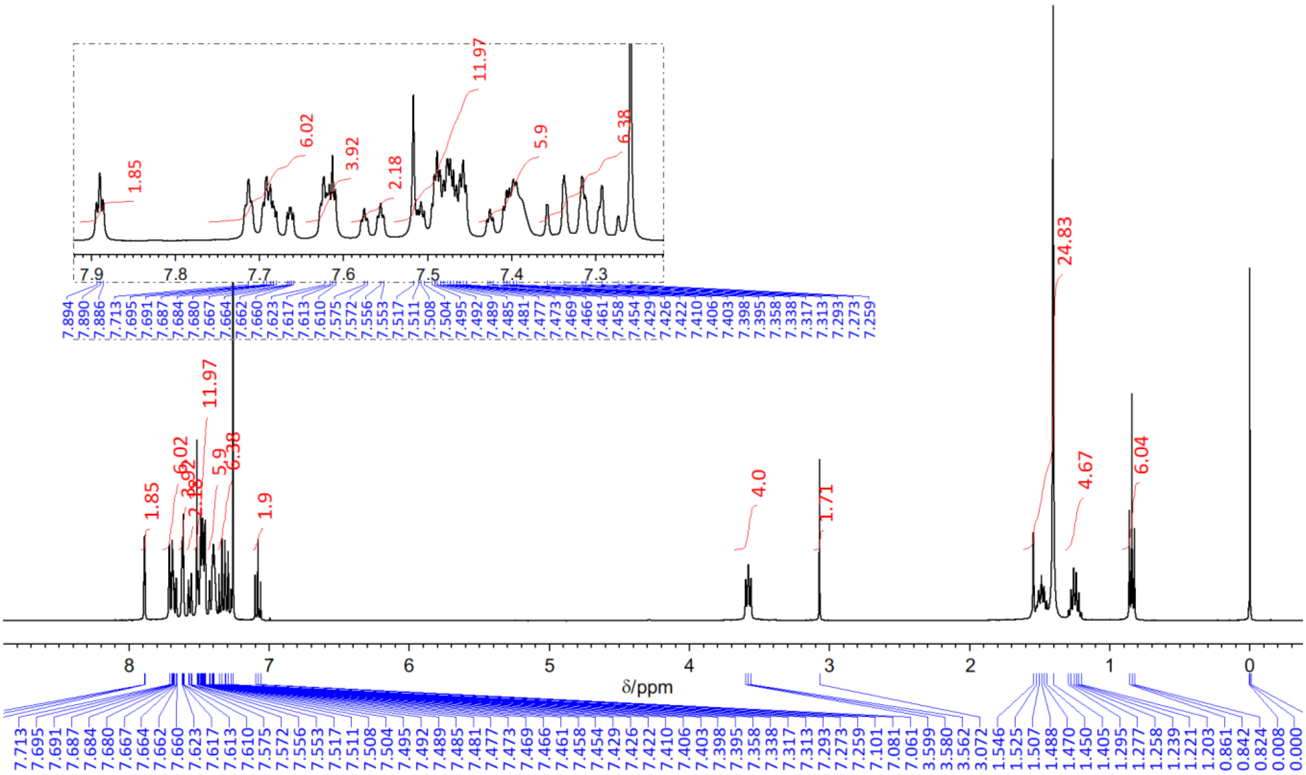

^{1}H NMR spectrum (400 MHz) of **7**, measured in chloroform-*d* at room temperature.

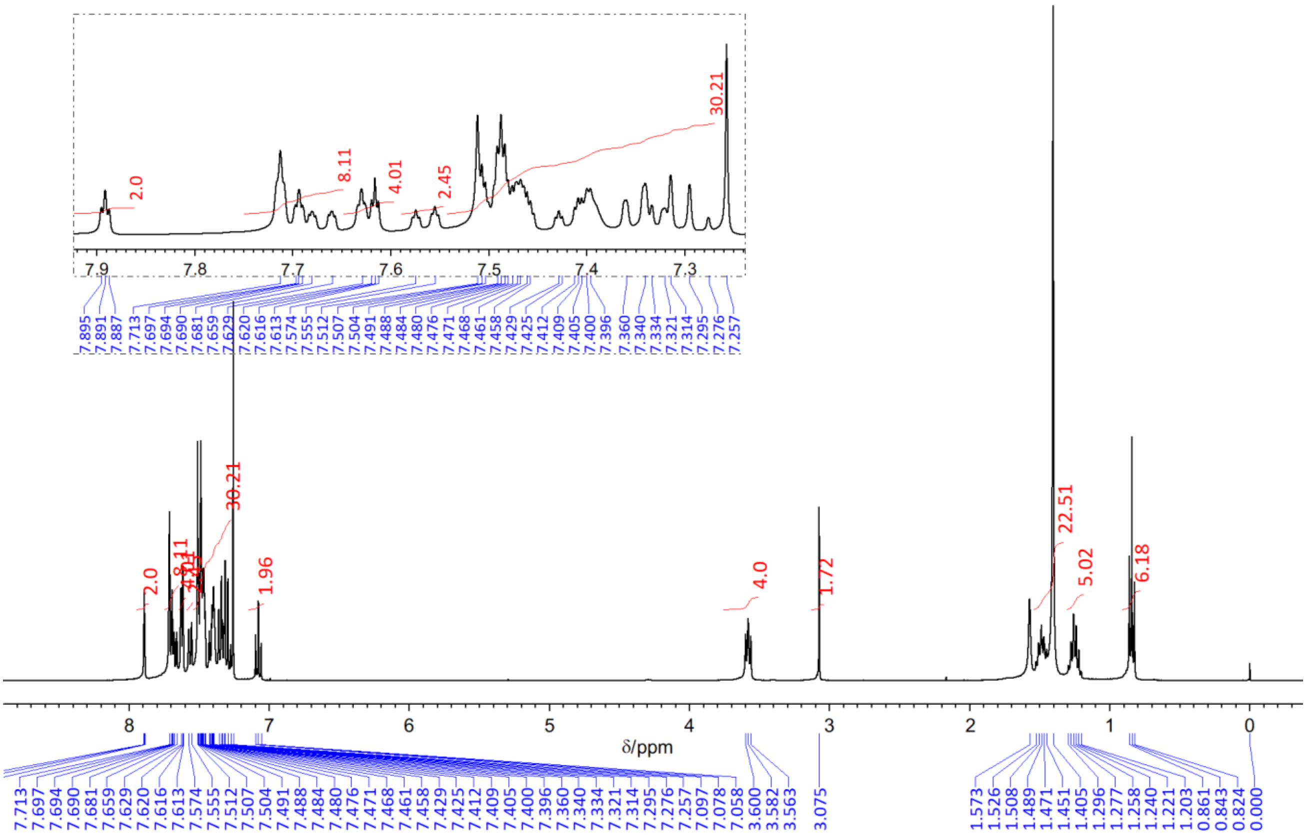

^{1}H NMR spectrum (400 MHz) of **8**, measured in chloroform-*d* at room temperature.

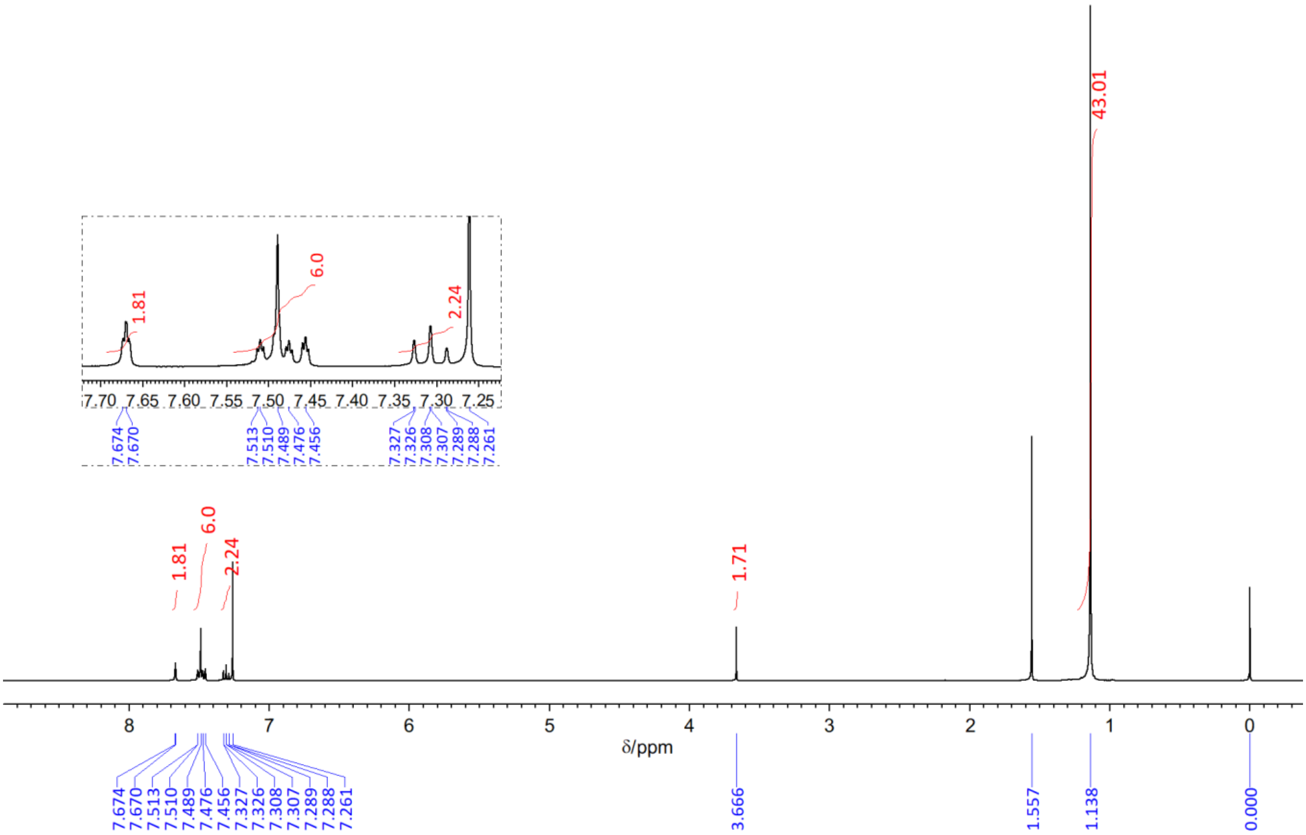

^{1}H NMR spectrum (400 MHz) of **17'**, measured in chloroform-*d* at room temperature.

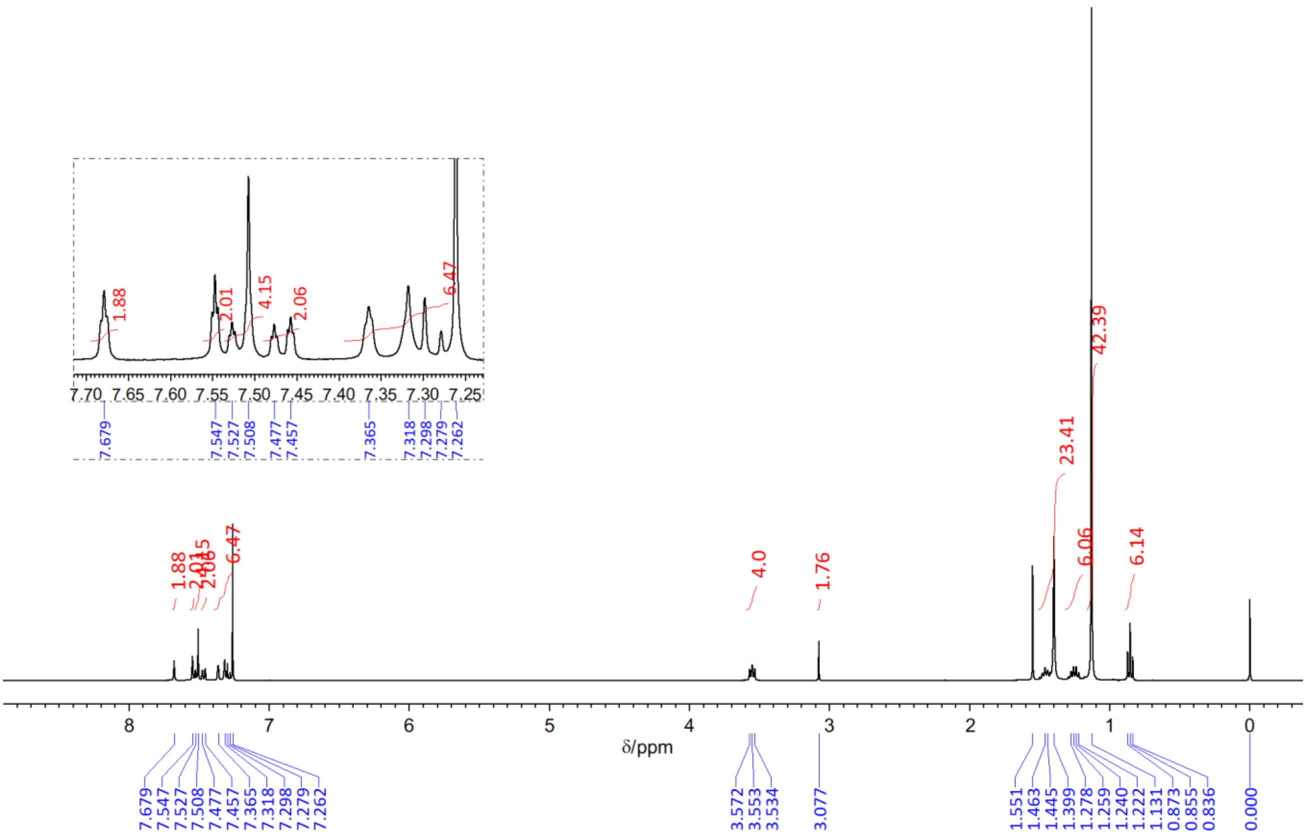

^{1}H NMR spectrum (400 MHz) of **18'**, measured in chloroform-*d* at room temperature.

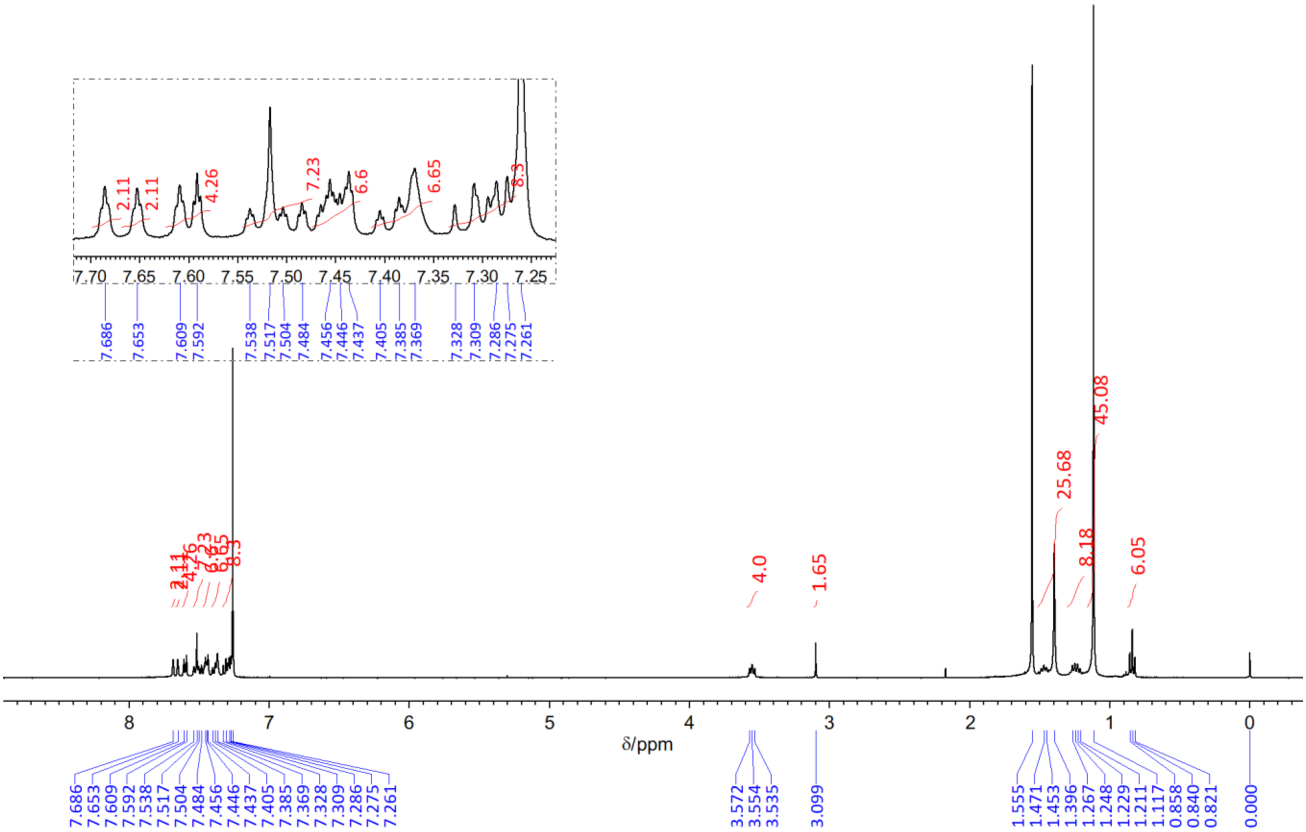

^{1}H NMR spectrum (400 MHz) of **25'**, measured in chloroform-*d* at room temperature.

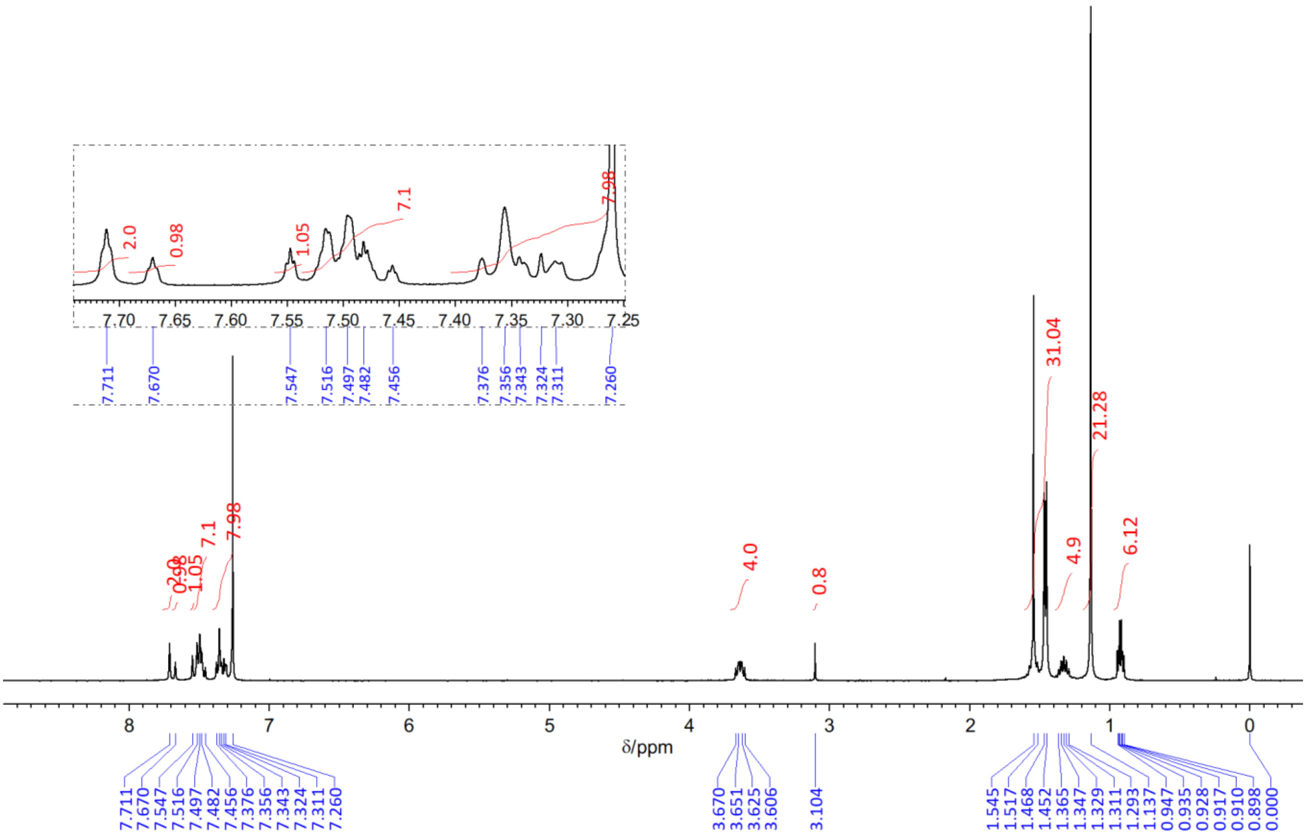

^{1}H NMR spectrum (400 MHz) of **29'**, measured in chloroform-*d* at room temperature.

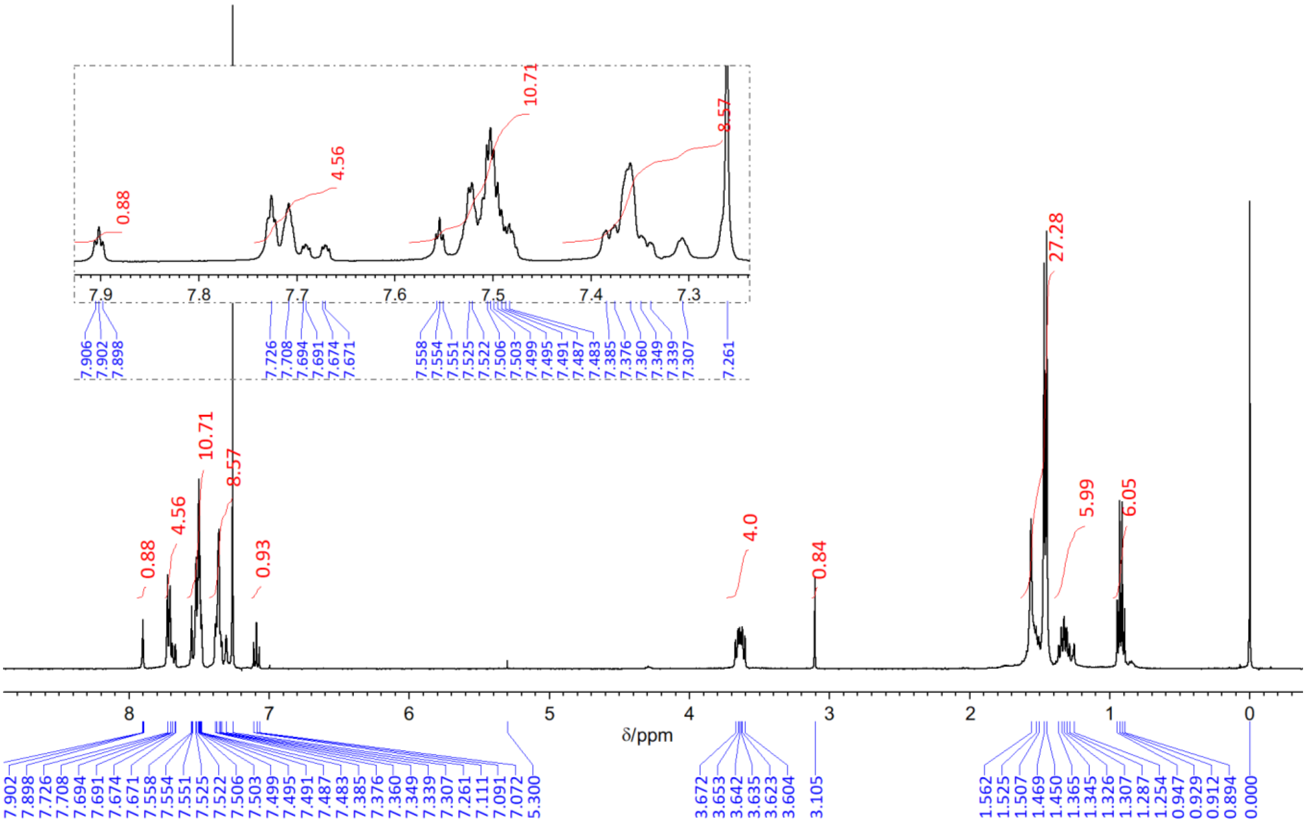

^{1}H NMR spectrum (400 MHz) of **30'**, measured in chloroform-*d* at room temperature.

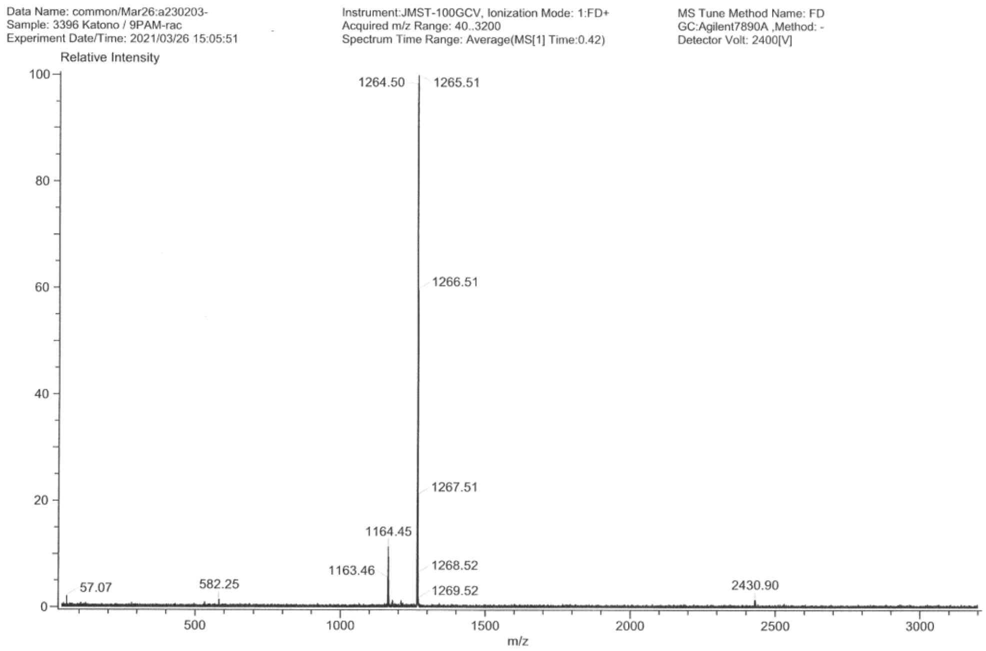

LR-MS (FD) spectrum of *rac*-**3** (9PAM).

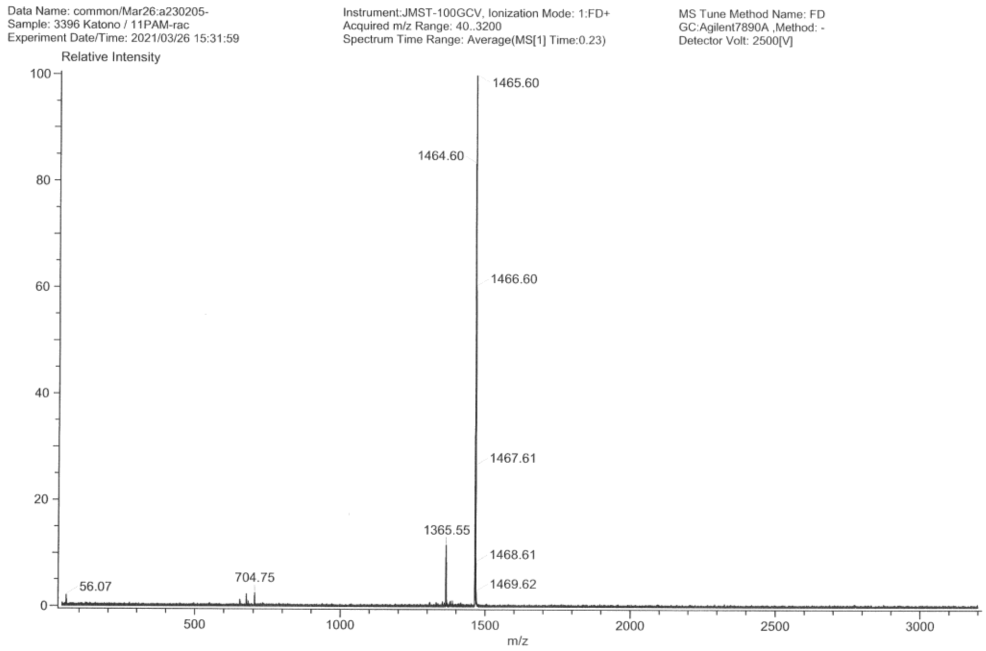

LR-MS (FD) spectrum of *rac*-**4** (11PAM).

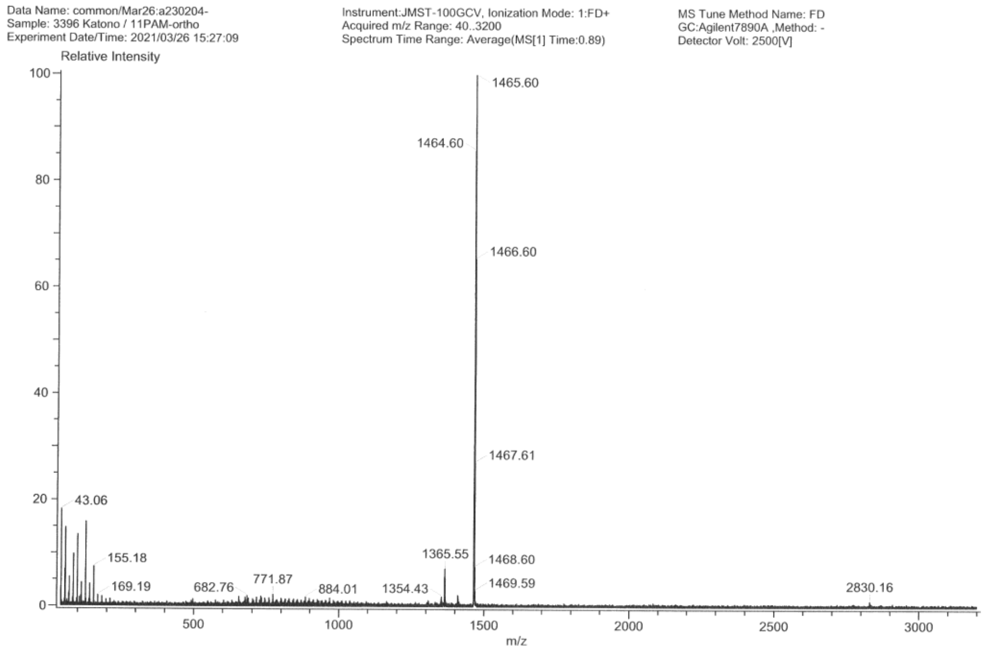

LR-MS (FD) spectrum of **10** (11PAM).

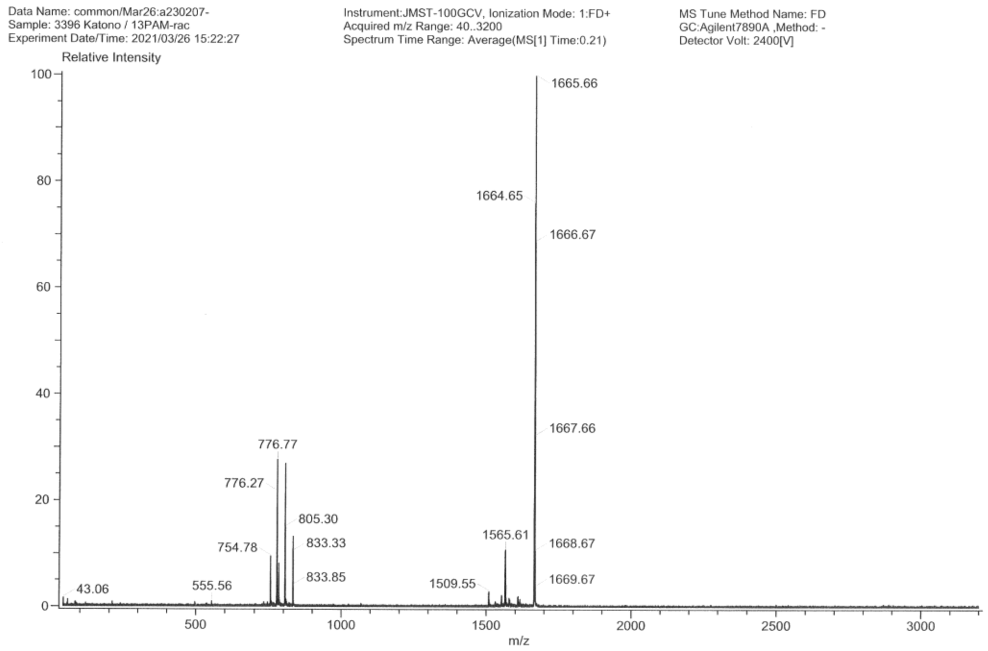

LR-MS (FD) spectrum of *rac*-**5** (13PAM).

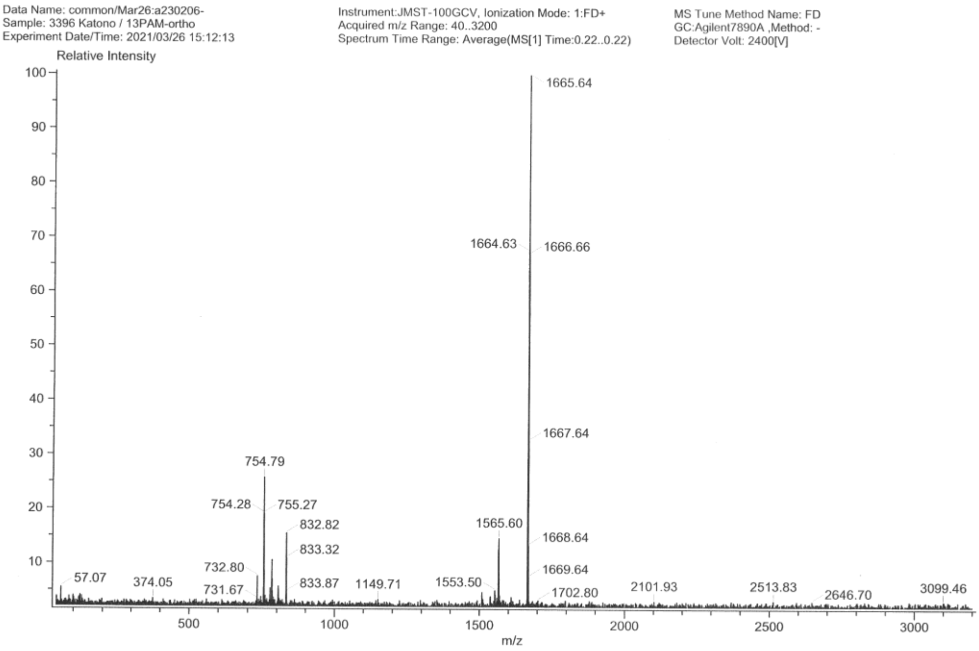

LR-MS (FD) spectrum of **11** (13PAM).

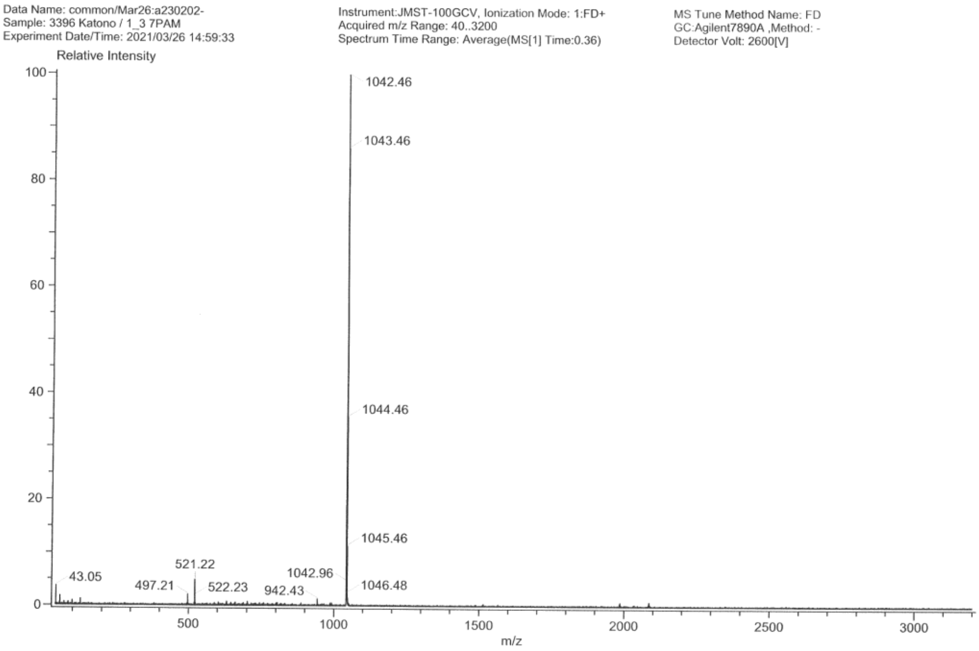

LR-MS (FD) spectrum of **14** (7PAM).

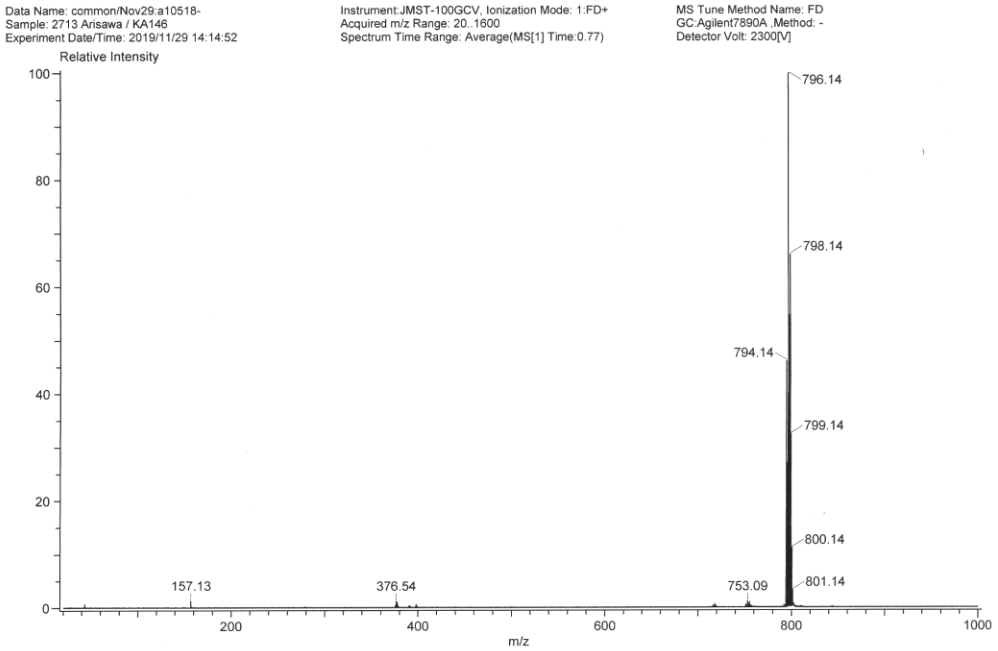

LR-MS (FD) spectrum of **16**.

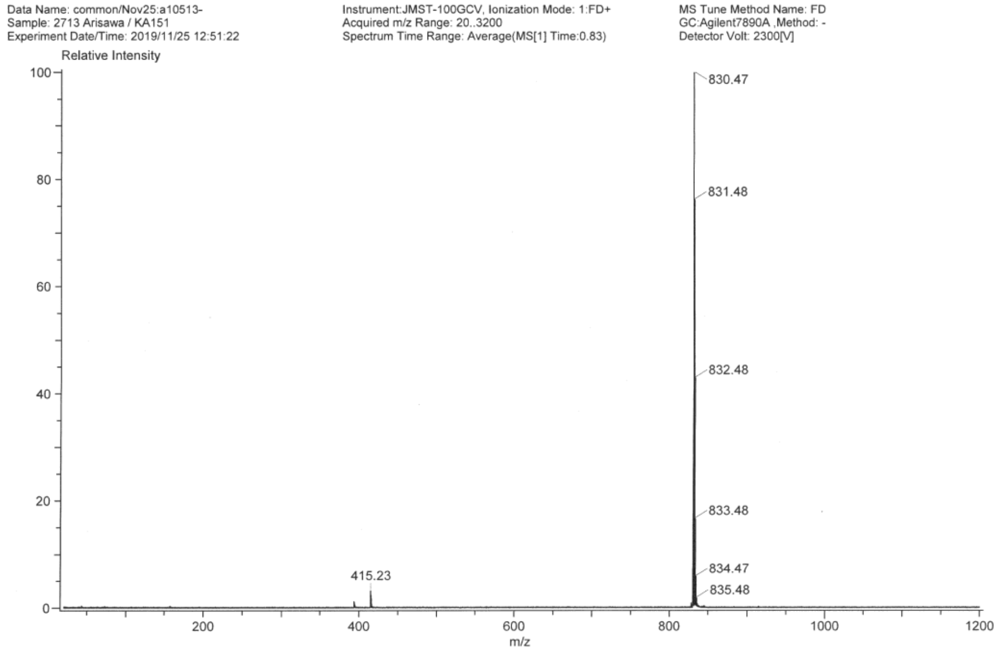

LR-MS (FD) spectrum of **17**.

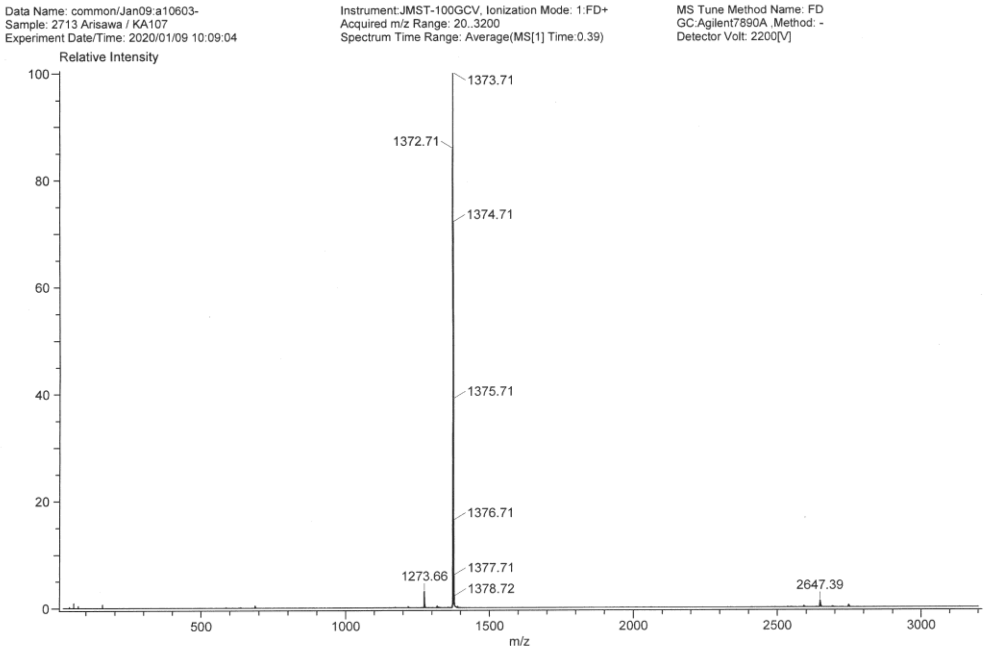

LR-MS (FD) spectrum of **18**.

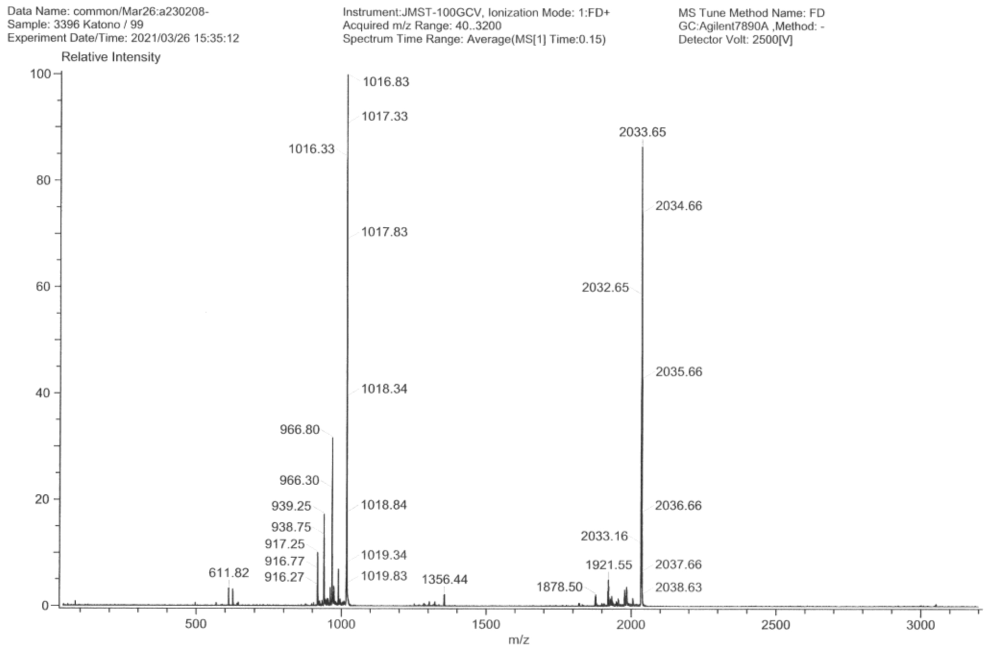

LR-MS (FD) spectrum of **19**.

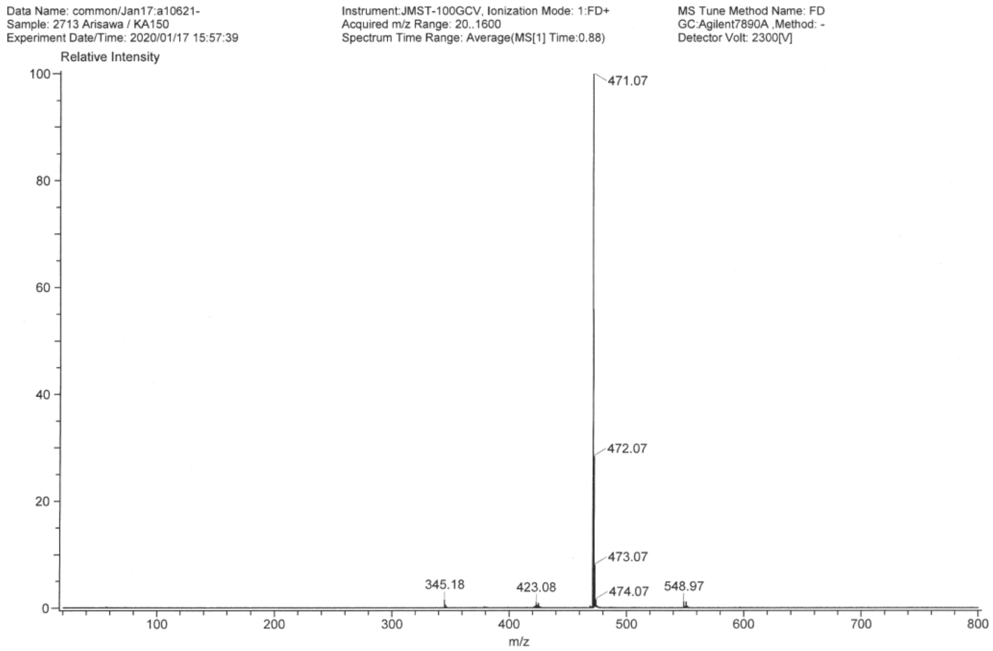

LR-MS (FD) spectrum of **21**.

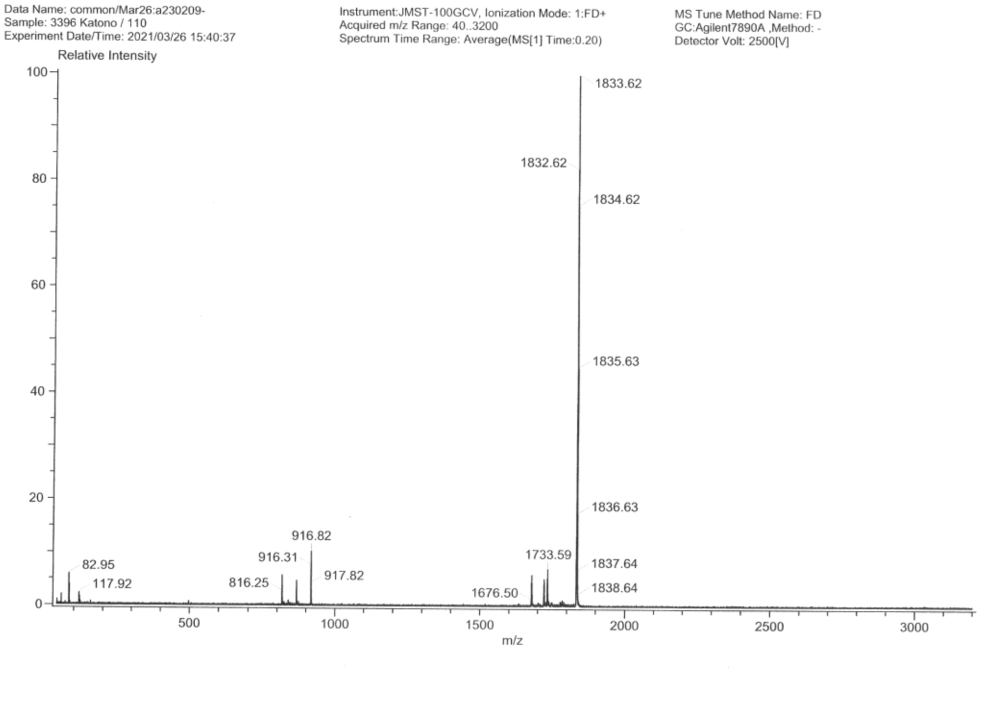

LR-MS (FD) spectrum of **24**.

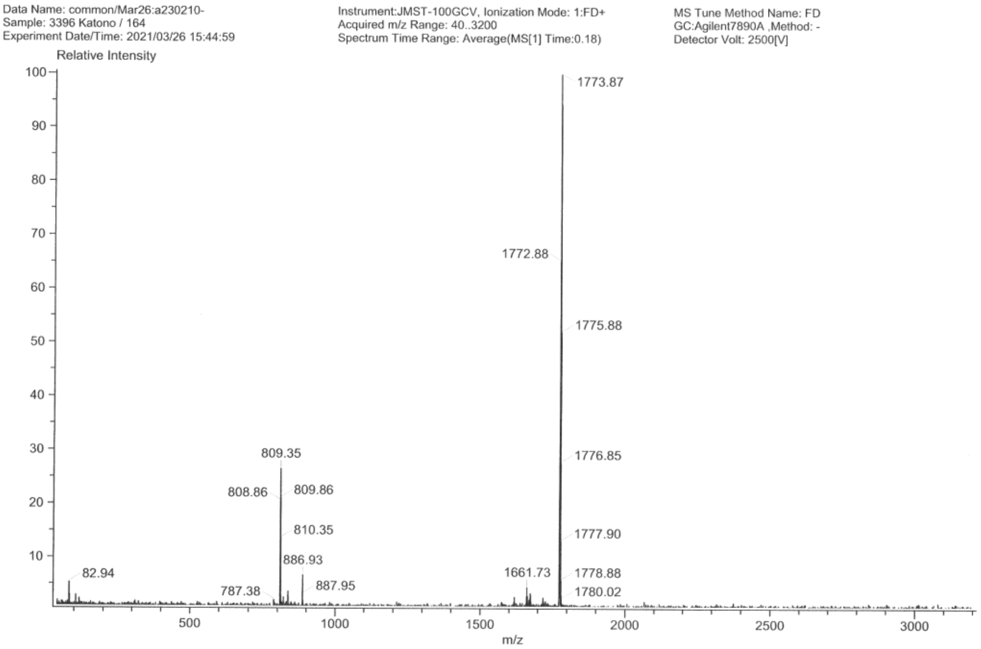

LR-MS (FD) spectrum of **25**.

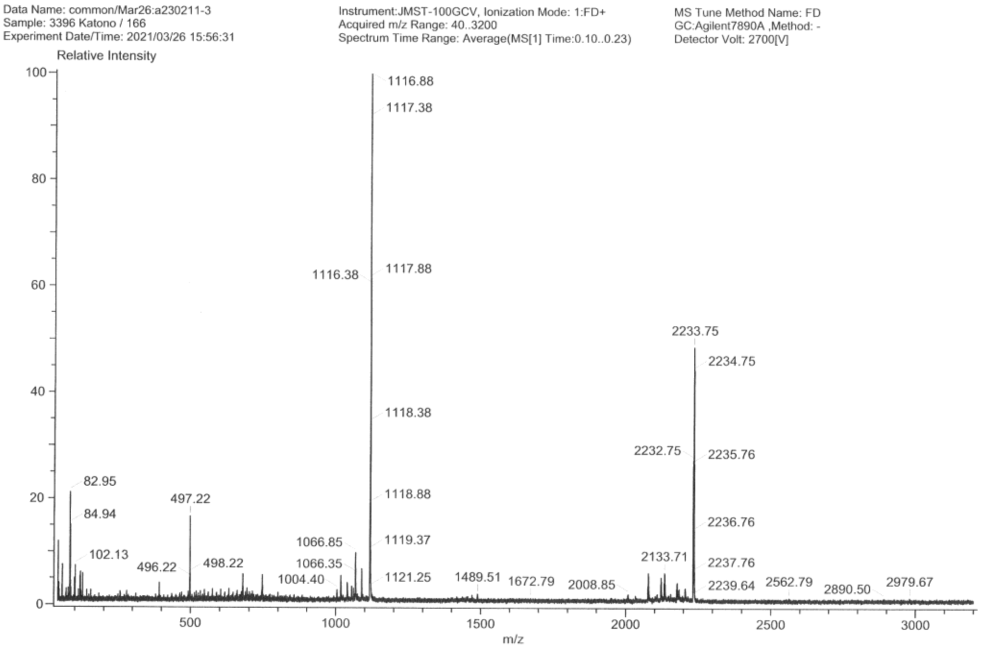

LR-MS (FD) spectrum of **26**.

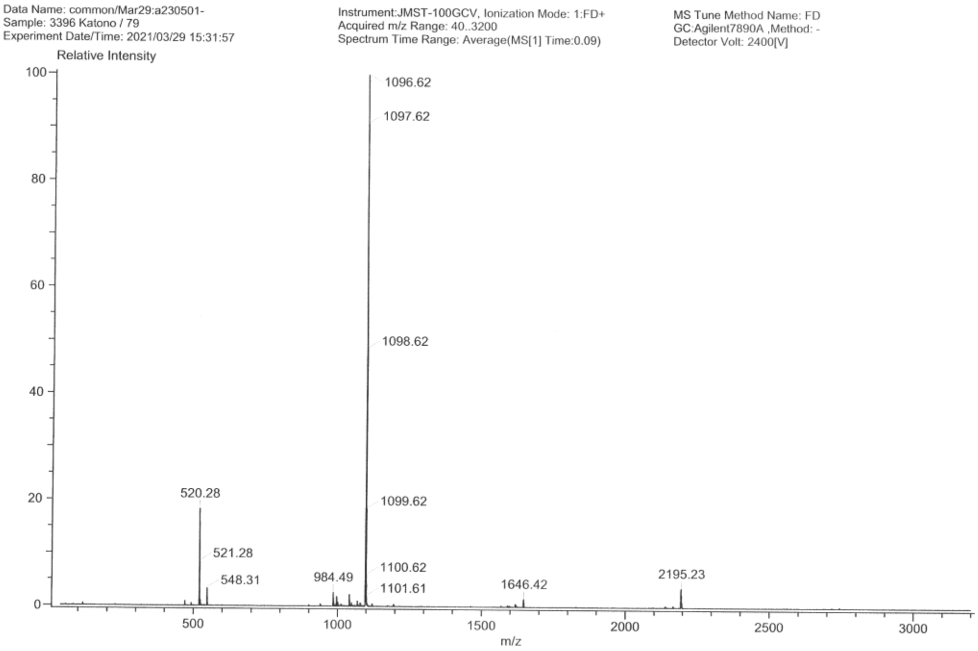

LR-MS (FD) spectrum of **29**.

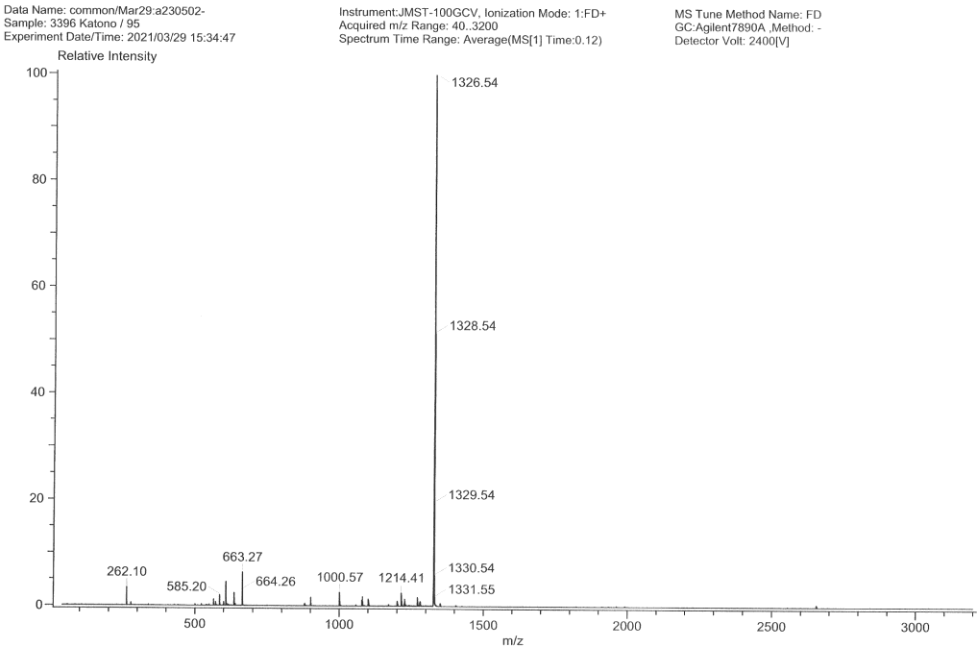

LR-MS (FD) spectrum of **30**.
